# Uncovering the Mechanism of Azepino‐Indole Skeleton Formation via Pictet–Spengler Reaction by Strictosidine Synthase: A Quantum Chemical Investigation

**DOI:** 10.1002/open.202300043

**Published:** 2023-05-30

**Authors:** Mingqi Mou, Chenghua Zhang, Shiqing Zhang, Fuqiang Chen, Hao Su, Xiang Sheng

**Affiliations:** ^1^ Tianjin Institute of Industrial Biotechnology Chinese Academy of Sciences Tianjin 300308 P.R. China; ^2^ University of Chinese Academy of Sciences 19 A Yuquan Road Beijing 100049 P.R. China; ^3^ School of Pharmacy North Sichuan Medical College Nanchong 637100 P.R. China; ^4^ National Center of Technology Innovation for Synthetic Biology National Engineering Research Center of Industrial Enzymes and Key Laboratory of Engineering Biology for Low-Carbon Manufacturing Tianjin 300308 P.R. China

**Keywords:** reaction mechanisms, density functional theory calculations, Pictet−Spengler reaction, quantum chemistry, strictosidine synthase

## Abstract

Strictosidine synthase (STR) catalyzes the Pictet–Spengler (PS) reaction of tryptamine and secologanin to produce strictosidine. Recent studies demonstrated that the enzyme can also catalyze the reaction of non‐natural substrates to form new alkaloid skeletons. For example, the PS condensation of 1*H*‐indole‐4‐ethanamine with secologanin could be promoted by the STR from *Rauvolfia serpentina* (*Rs*STR) to generate a rare class of skeletons with a seven‐membered ring, namely azepino‐[3,4,5‐cd]‐indoles, which are precursors for the synthesis of new compounds displaying antimalarial activity. In the present study, the detailed reaction mechanism of *Rs*STR‐catalyzed formation of the rare seven‐membered azepino‐indole skeleton through the PS reaction was revealed at the atomic level by quantum chemical calculations. The structures of the transition states and intermediates involved in the reaction pathway were optimized, and the energetics of the complete reaction were analyzed. Based on our calculation results, the most likely pathway of the enzyme‐catalyzed reaction was determined, and the rate‐determining step of the reaction was clarified. The mechanistic details obtained in the present study are important in understanding the promiscuous activity of *Rs*STR in the formation of the rare azepino‐indole skeleton molecule and are also helpful in designing STR enzymes for the synthesis of other new alkaloid skeleton molecules.

## Graphical abstract

The detailed reaction mechanism of the Pictet–Spengler condensation of 1*H*‐indol‐4‐ethanamine with secologanin by strictosidine synthase was revealed by quantum chemical cluster calculations.

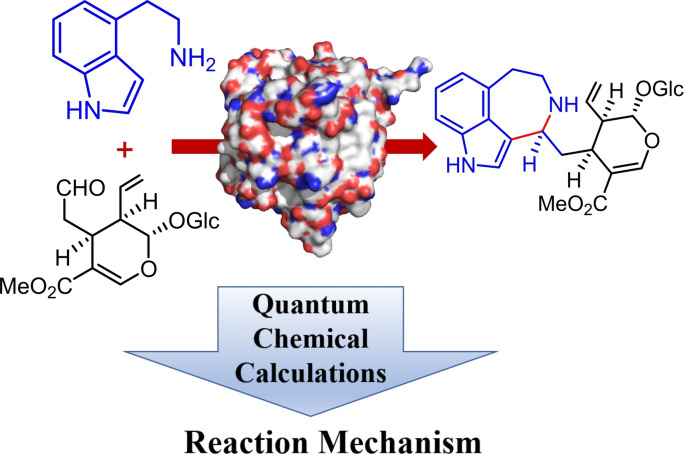

## Introduction

The Pictet–Spengler (PS) reaction is an important method for the synthesis of alkaloid skeletons.[^1^, ^2^] The typical PS reaction is a condensation reaction of β‐arylethylamine with carbonyl compounds under acidic conditions to form an iminium intermediate, which then reacts with the electron‐rich aromatic ring to form a new C−C single bond to obtain nitrogen heterocyclic skeletons. These skeletons can generate different alkaloids after structural modification, such as tetrahydroisoquinolines and tetrahydro‐β‐carboline alkaloids.

The enzymes catalyzing the PS reactions in nature are known as Pictet–Spenglerases (PSases).^[3]^ Since the enzymatic PS reaction proceeds under mild conditions and displays high stereoselectivity, it is a promising alternative to the traditional organic synthesis methods.[^2^, ^3^] At present, a number of PSases have been identified from different alkaloid synthesis pathways.^[3]^ The norcoclaurine synthase (NCS) and strictosidine synthase (STR) involved in the synthesis of tetrahydroisoquinoline and tetrahydro‐β‐carboline alkaloids, respectively, have attracted much attention.[^2^, ^3^]

**Figure 1:**
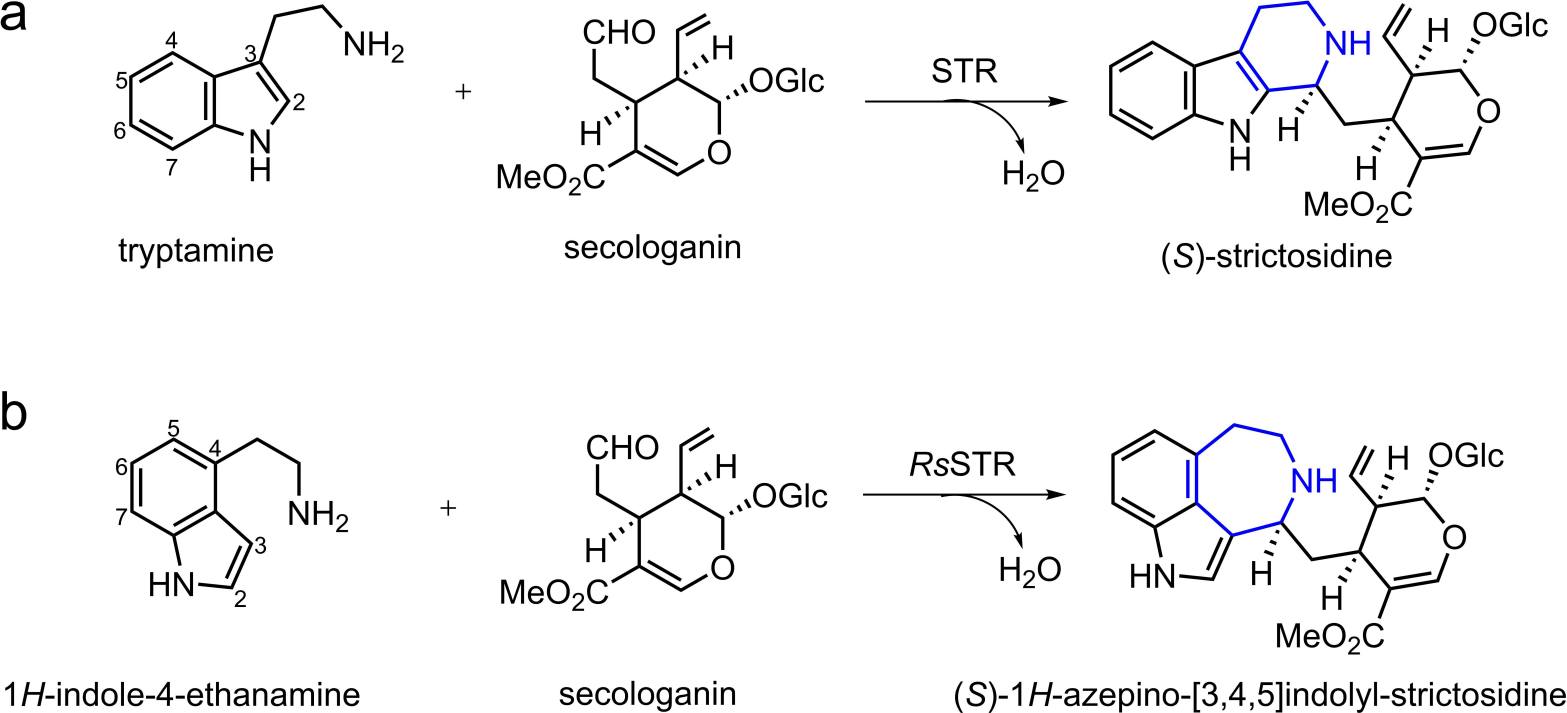
STR‐catalyzed reaction of secologanin with (a) tryptamine and (b) 1*H*‐indole‐4‐ethanamine.

**Figure 2:**
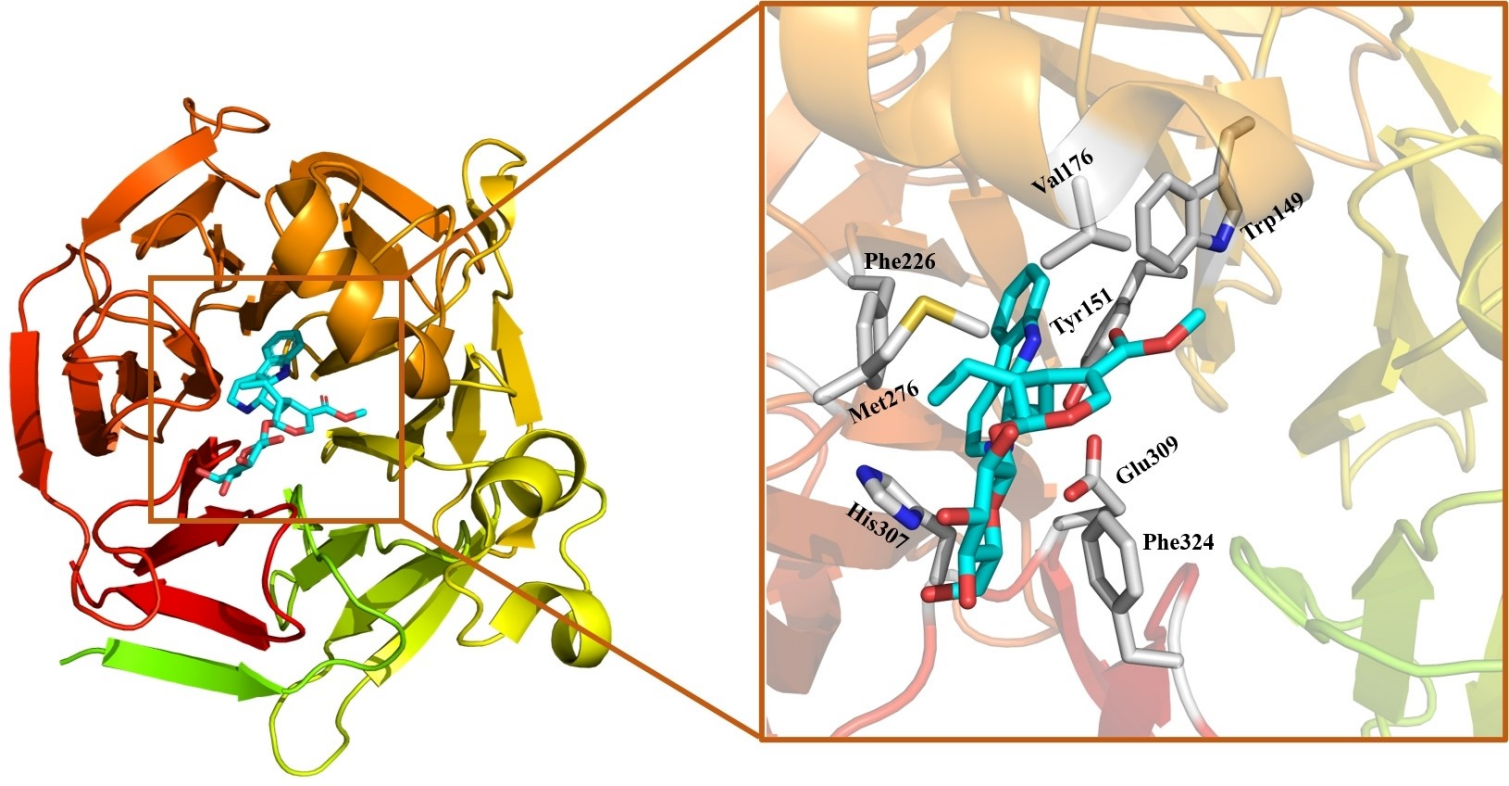
Crystal structure and active site of the STR from *Rauvolfia serpentina* in complex with the strictosidine product (PDB ID: 2V91).

In nature, STR catalyzes the PS reaction of secologanin with tryptamine to produce α‐(*S*)‐strictosidine (Figure 1a), which is a common biosynthetic precursor of many plant‐derived monoterpene indole alkaloids of pharmaceutical interest.^[4]^ The first STR was isolated from the plant cell suspensions of *Catharanthus roseus* (*Cr*STR).^[5]^ More STR enzymes were then identified and characterized from other organisms such as *Rauvolfia serpentina* (*Rs*STR) and *Ophiopogon pumila* (*Op*STR).^[6]^ A number of crystal structures of these STR enzymes have been solved in various forms.[^6c^, ^7^, ^8^] Analysis of the structures of *Rs*STR in complex with either tryptamine (PDB ID: 2FPB) or secologanin (PDB ID: 2FPC) showed that tryptamine is buried in the active site and secologanin is bound in the binding entrance.^[6c]^ Interestingly, the structure of *Rs*STR in complex with the product was solved (PDB ID: 2V91, Figure 2) and used to guide the re‐design of the enzyme.^[7a]^

It has been demonstrated that the STR enzymes exhibit broad substrate scope and have high enantioselectivity.[^3^, ^9^] Interestingly, they display opposite enantiopreferences toward different aldehyde substrates. Namely, the (*S*)‐product is formed exclusively in the STR reaction of tryptamine with the natural secologanin substrate, while the (*R*)‐product is preferred when tryptamine reacts with short‐chain aliphatic aldehydes, for example, isovaleraldehyde and butyraldehyde.[^7e^, ^10^]

A recent study showed that *Rs*STR displays promiscuous activity in the formation of a rare azepino‐[3,4,5‐cd]‐indole alkaloid skeleton by the PS reaction of 1*H*‐indole‐4‐ethanamine (4‐IEA) with secologanin.^[8]^ Compared with the reaction of the natural substrates, in which a six‐membered ring is formed in the product, the newly generated nitrogen heterocycle in this reaction is a seven‐membered ring. As many known azepino‐indole‐derived compounds are related to antimalarial and psychotropic drugs,^[8]^ *Rs*STR is a promising enzyme to be utilized for the biocatalytic synthesis of the precursors of new alkaloids with pharmacological activities.

The reaction mechanisms and selectivities of *Rs*STR toward both natural and non‐natural short‐chain aldehyde substrates were recently studied by using quantum chemical calculations.^[11]^ It was shown that it follows the general reaction mechanism of the PS reaction. First, the protonated amino group of tryptamine transfers a proton to a glutamate residue (Glu309), followed by a concerted step consisting of a proton transfer from Glu309 to the secologanin substrate and a C−N bond formation. Then, an iminium intermediate is formed by dehydration. After a rotation of the indole ring of the tryptamine moiety, the iminium intermediate undergoes a ring closure reaction and a successive deprotonation to obtain the product.

Moreover, it was shown that the natural and non‐natural substrates bind to the active site in different manners.^[11]^ For the natural substrates, due to the large size of secologanin, tryptamine would bind to the active site first,^[11]^ and the binding of tryptamine was recently proposed to induce conformational changes that are beneficial in recognizing secologanin.^[12]^ For the non‐natural aldehyde substrates, since the short‐chain aldehydes are similar in size to the other substrate tryptamine, two substrates can bind to the active site in a random order.^[11]^

At present, the mechanism of STR‐catalyzed PS reaction of the non‐natural substrate 1*H*‐indole‐4‐ethanamine with secologanin, producing the azepino‐indole skeleton molecule, remains unsolved. Therefore, quantum chemical calculations are performed in the present study to investigate this promiscuous activity of *Rs*STR. According to a previous study on the reaction of the natural substrates, the natural reaction follows the tryptamine‐first model,^[11]^ it can be envisioned that the substrates focused in the present study follows the same binding mode. On the basis of the obtained enzyme‐substrates complex, the structures of the intermediates and transition states involved in the reaction pathway were optimized, and the corresponding energetics were analyzed. A reaction pathway with feasible energies was determined and the influence of some key residues on the enzyme activity was revealed.

## Computational Details

All the calculations in this study were performed using the B3LYP−D3(BJ) hybrid density functional method^[13]^ with the Gaussian16 program.^[14]^ Geometry optimizations were performed on the 6‐31G(d,p) basis set. At the same theoretical level, the SMD solvation^[15]^ (ϵ=4) was used to calculate the single‐point energy to estimate the influence of the surrounding environment on the energies. According to the literature, the choice of the dielectric constant value will not have a great influence on the system when a large active site model is used for the calculations with the cluster approach.^[16]^ In order to obtain more accurate electron energies, the single point energies of the optimized structures were calculated with a larger basis set, namely 6‐311+G (2d,2p). Frequencies were calculated at the same level of geometry optimization to obtain the zero‐point energies (ZPEs). The energy values reported in this paper are dispersion‐included large basis set energies after solvation effect and ZPE corrections. The chosen level of theory in the present study has widely used in the computational studies of enzymes, and has been shown to provide reliable results.^[17]^

**Figure 3:**
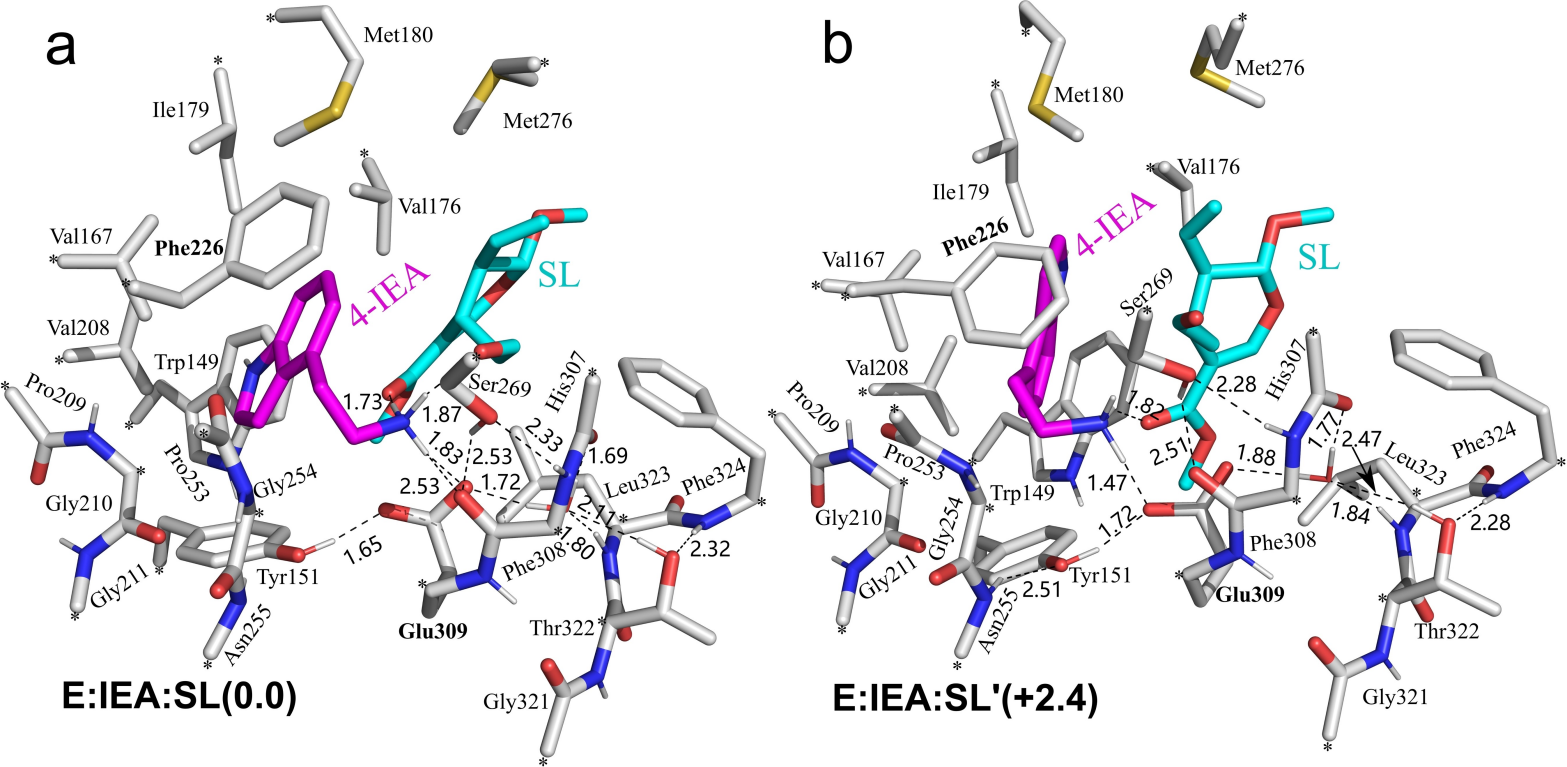
Two lowest‐energy structures for the active site model of *Rs*STR in complex with the 1*H*‐indole‐4‐ethanamine (4‐IEA) and secologanin (SL) substrates. The fixed atom is marked with “*”, and the distances are given in Å.

The quantum chemical cluster approach was used in the present study. This method has been established to be powerful in investigating the mechanistic details of enzymatic reactions, and recently also in rationalizing various selectivities and in predicting mutations.^[17]^ The active site model was designed on basis of the crystal structure of *Rs*STR in complex with the strictosidine product (PDB ID: 2V91).^[7a]^ To construct the enzyme‐substrates complex for the current study, strictosidine was replaced by the two substrates, 1*H*‐indole‐4‐ethanamine and secologanin. By analyzing the interaction between the substrates and surrounding residues and the residues themselves, an active site model containing 341 atoms was obtained. In addition to the two substrates and one water molecule, a large number of residues assembling the binding pocket are included in the model, consisting of Trp149, Tyr151, Val167, Val176, Ile179, Met180, Val208, Pro209, Gly210, Gly211, Phe226, Pro253, Gly254, Asn255, Ser269, Met276, His307, Phe308, Glu309, Gly321, Thr322, Leu323 and Phe324 (Figure 3).

Both the amino acids and the secologanin substrate were truncated, and hydrogen atoms were added to the truncated positions to saturate the truncated bonds. The truncation of secologanin was made at the glycosidic bond. During the geometry optimization, the truncated carbon atoms were fixed (marked with “*” in Figure 3). Although constrains were introduced to the edge of the active site model, the size of the model is sufficient to ensure the flexibility of the active site, and necessary conformational changes of the side chains can occur during the reaction. In the employed model, the amino group of 1*H*‐indole‐4‐ethanamine is protonated, and Glu309 is in the deprotonated state. The total charge of the model is 0.

## Results and Discussion

### Substrate Binding Mode

Due to the difference in the chemical structures, the non‐natural substrate 1*H*‐indole‐4‐ethanamine could bind to the active site in a different manner compared to the natural substrate tryptamine. A variety of substrate binding modes with different conformations, including the orientation of substrates in the active site and the hydrogen bond patterns, were optimized and the corresponding energies were evaluated (see the Supporting Information). The structure of the enzyme‐substrates complex with the lowest energy (called **E:IEA:SL**) is shown in Figure 3a. However, it was proved by the calculations that the pathway starting from this enzyme‐substrates complex is energetically infeasible because the barrier for the first step is already very high (26.0 kcal/mol, see Supporting Information). Instead, a binding mode with slightly higher energy by 2.4 kcal/mol than **E:IEA:SL** (called **E:IEA:SL′**, Figure 3b) was found to have feasible barriers and was thus the productive mode adopted by the enzyme.

In the optimized structure of **E:IEA:SL**, the protonated amino group of 1*H*‐indole‐4‐ethanamine forms hydrogen bonds with the Glu309 residue and the methoxycarbonyl and aldehyde groups of secologanin. An π–π interaction is observed between the indole ring of 1*H*‐indole‐4‐ethanamine and Phe226. In the case of **E:IEA:SL′**, the hydrogen bond interaction with Glu309 and the π–π interaction with Phe226 are also present for the 1*H*‐indole‐4‐ethanamine. However, different from **E:IEA:SL**, the hydrogen bond with the aldehyde group cannot be formed because of the different orientation of the substrates.

The other optimized structures of the enzyme‐substrates complexes were calculated to have higher energies than **E:IEA:SL**, and are thus not the preferred binding modes (see the Supporting Information). However, the discussion of these structures is also helpful for the understanding of the binding pose of the non‐natural substrate. First, we noticed that the tryptamine substrate in the previous study on the natural reaction forms a hydrogen bond with Pro209.^[11]^ We here considered a similar orientation for 1*H*‐indole‐4‐ethanamine, and the calculated energy of the optimized structure is 21.1 kcal/mol higher than **E:IEA:SL**. Additionally, we also considered a structure in which the 1*H*‐indole‐4‐ethanamine substrate is orientated in a similar manner as the tryptamine moiety in the solved structure of enzyme‐product complex. The calculated energy is also much higher than **E:IEA:SL** (by 15.2 kcal/mol).

### Reaction Mechanism

**Figure 4:**
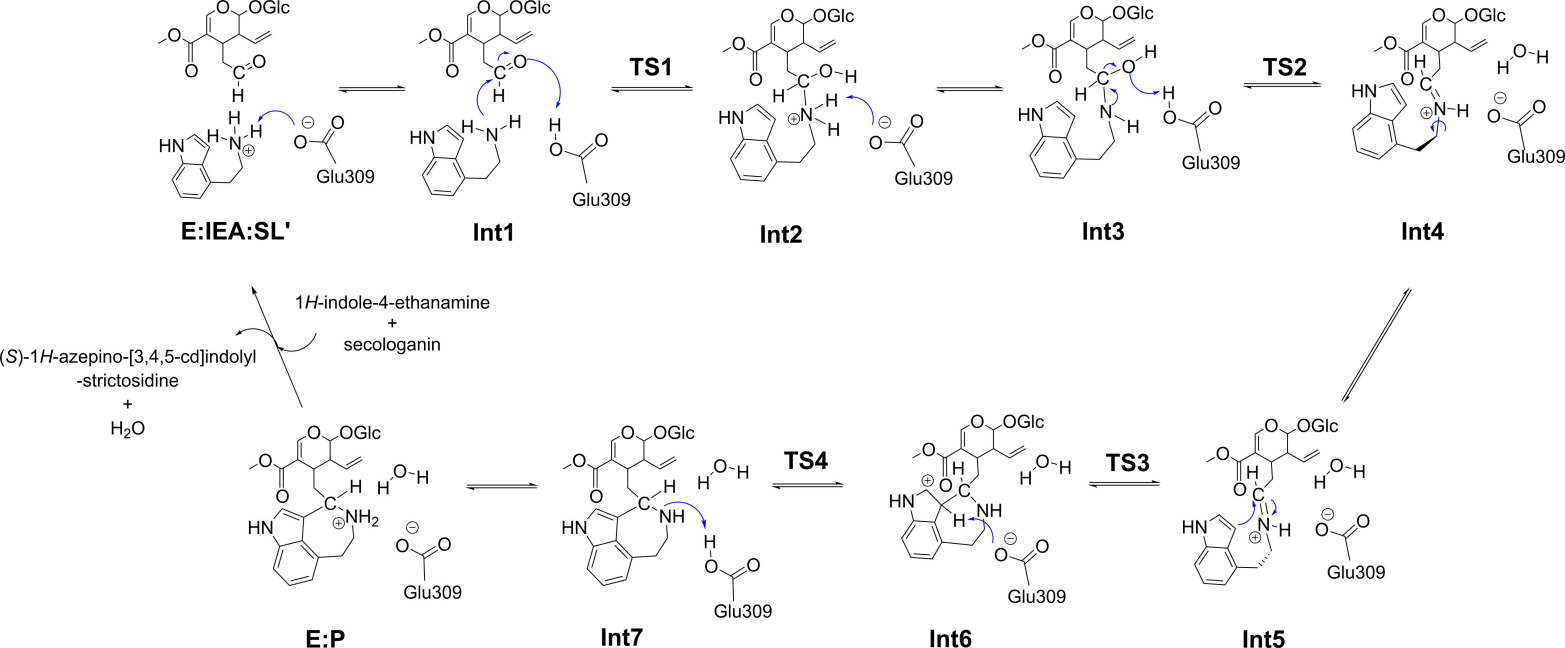
The detailed reaction mechanism of *Rs*STR‐catalyzed PS reaction of 1*H*‐indole‐4‐ethanamine with secologanin by quantum chemical calculations in this study.

**Figure 5:**
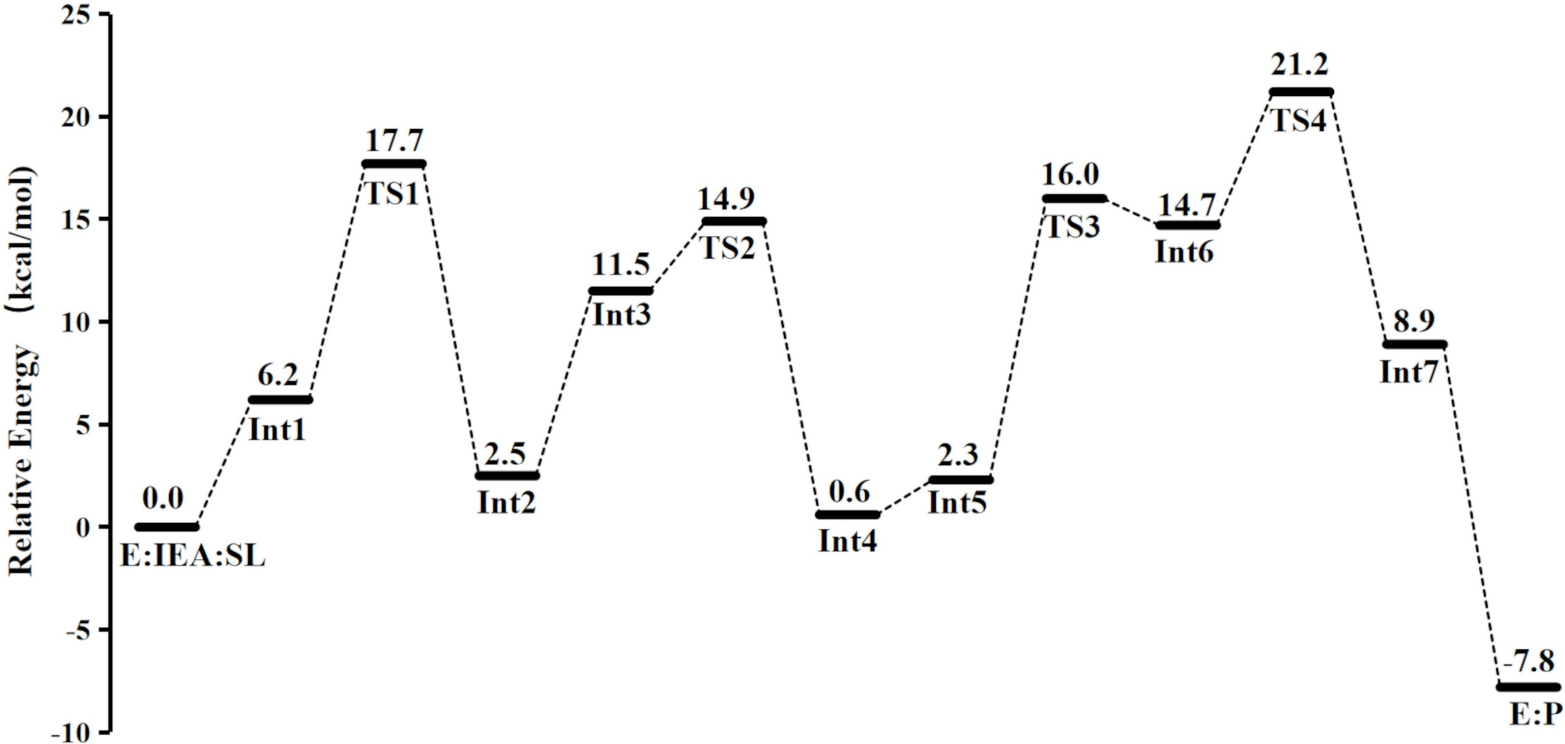
Calculated energy profile of *Rs*STR‐catalyzed PS reaction of 1*H*‐indole‐4‐ethanamine with secologanin.

**Figure 6:**
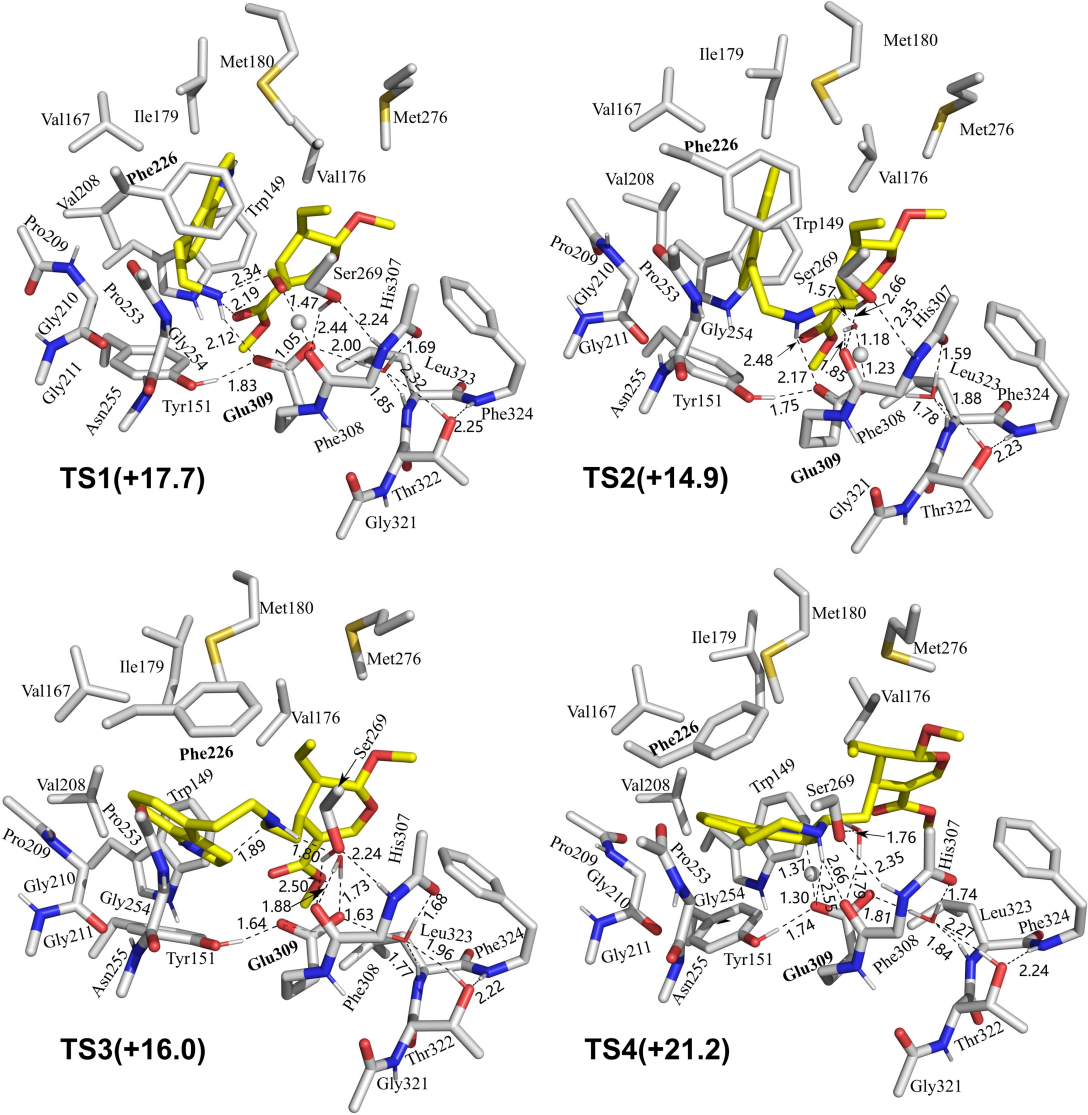
Optimized structures of the transition states involved in the *Rs*STR reaction of 1*H*‐indole‐4‐ethanamine with secologanin.

On the basis of the optimized structures of the enzyme‐substrates complexes with low energies, the reaction mechanism of STR‐catalyzed condensation of the non‐natural 1*H*‐indole‐4‐ethanamine substrate with secologanin was studied in detail. According to the calculations, the reaction mechanism starting from **E:IEA:SL′** has feasible energy barriers. The detailed reaction mechanism is shown in Figure 4, and the calculated energy profile of the complete reaction pathway is shown in Figure 5. The optimized structures of transition states are shown in Figure 6, and structures of the intermediates are given in the Supporting Information.

According to the calculations, the reaction of 1*H*‐indole‐4‐ethanamine with secologanin catalyzed by STR essentially follows the general mechanism of the Pictet–Spengler reaction and is the same in sequence of the elementary steps as reaction of the natural substrates.^[11]^ First, the protonated amino group of 4‐IEA transfers a proton to Glu309 to initiate the condensation reaction between the two substrates (Figure 4), and the energy of the formed intermediate **Int1** is 6.2 kcal/mol higher than **E:IEA:SL** (Figure 5).

Then, **Int1** undergoes a concerted elementary reaction to form **Int2**, which involves the formation of a C−N bond and the proton transfer from Glu309 to the carbonyl group of the secologanin substrate. The calculated energy of **Int2** is almost the same as **E:IEA:SL′**, which is 2.5 kcal/mol higher than **E:IEA:SL** (Figure 5). In the transition state of this step (**TS1**), the C−N bond distance is 2.34 Å (Figure 6). The reaction is then followed by the proton transfer to Glu309 (**Int2**→**Int3**) and the subsequent dehydration reaction, forming the key iminium intermediate (**Int4**). The energy barrier of the dehydration step (**TS2**) is 14.9 kcal/mol relative to **E:IEA:SL**, and the distance between oxygen of Glu309 and hydrogen is 1.23 Å (Figure 6).

Next, a conformational change of **Int4** takes place by the rotation of the indole ring. The resulted **Int5** has an increased energy compared to **Int4** (by 1.7 kcal/mol). After that, the cyclization occurs by the nucleophilic attack of C3 of the indole ring on the carbon atom of the iminium intermediate (**Int5**→[**TS3**]→**Int6**), leading to the formation of a seven‐membered ring. In the reaction of the natural substrates, the cyclization takes place at the C2 position of the indole ring to form a six‐membered cyclic amine, and the cyclization at the C3 position would form a spiroindolenine intermediate which has been proved to be infeasible under STR catalysis.^[11]^ The C−C distance in **TS3** is 1.89 Å, and is then further shortened to 1.68 Å in **Int6**. The energy barrier of this step is calculated to be 16.0 kcal/mol relative to **E:IEA:SL**. Finally, the azepino‐[3,4,5‐cd]‐indole product is generated by the deprotonation of C3–H with Glu309 acting as the general base group (**Int6**→[**TS4**]→**Int7**). The energy barrier of this step is 21.2 kcal/mol relative to **E:IEA:SL**. Interestingly, a proton transfer from Glu309 to the amine of the azepino‐[3,4,5‐cd]‐indole product greatly reduces the energy by 16.7 kcal/mol (**Int7**→**E : P**), indicating that amine group of the product prefers to be in the protonated form.

As shown in Figure 5, the proton transfer from C3–H of the cyclized intermediate to Glu309 is the rate‐determining step (RDS) of the reaction, and the calculated barrier is 21.2 kcal/mol. In the *Rs*STR‐catalyzed reaction of the natural substrates, the corresponding proton transfer step is also the RDS, with the energy barrier of 18.5 kcal/mol.^[11]^ Compared to the reaction of the natural substrates, the activation barrier is higher by 2.7 kcal/mol. This is in fact not surprising because the focused reaction in the present study is on the non‐natural substrate. Interestingly, the RDS of the NCS‐catalyzed PS reaction was demonstrated to be also the deprotonation step of the cyclized intermediate by a glutamate amino acid.^[18]^ It can thus be speculated that the deprotonation of the cyclized intermediate being the RDS is a common feature of PSases.

## Conclusions

In this study, the mechanism of the Pictet–Spengler reaction between 1*H*‐indole‐4‐ethanamine and secologanin by strictosidine synthase from *Rauvolfia serpentina* (*Rs*STR) was investigated in detail using the quantum chemical cluster approach. The calculations first showed that 1*H*‐indole‐4‐ethanamine binds to the active site in a different manner compared to the natural substrate. This indicates that the binding pocket of *Rs*STR could adjust to accommodate various substrates, providing a structural basis for the wide substrate scope of this enzyme. It has been demonstrated that the reaction follows the general PS reaction mechanism. First, after the proton transfer from the amino group of 1*H*‐indole‐4‐ethanamine to Glu309, the reaction undergoes a concerted step of C−N bond formation and proton transfer from Glu309 to the secologanin carbonyl group. Following the subsequent dehydration and the conformation change of the resulted iminium intermediate, cyclization takes place to develop a seven‐membered ring. The final product is generated by the deprotonation of the cyclized intermediate, which is the rate‐determining step according to the current calculations. We believe that the present study provides a complete picture of the reaction mechanism of *Rs*STR‐catalyzed PS condensation of 1*H*‐indole‐4‐ethanamine with secologanin, and the obtained information is valuable for better understanding of the STR enzymes and other PSases.

## Supporting Information Summary

Optimized structures of the enzyme‐substrate complexes and the transition state starting from **E:IEA:SL**, the structures of the intermediates and product involved in the lowest‐energy pathway, absolute energies and energy corrections, and Cartesian coordinates of the intermediates and transition states are contained in the Supporting Information.

## Conflict of interest

The authors declare no conflict of interest.

1

## Supporting information

As a service to our authors and readers, this journal provides supporting information supplied by the authors. Such materials are peer reviewed and may be re‐organized for online delivery, but are not copy‐edited or typeset. Technical support issues arising from supporting information (other than missing files) should be addressed to the authors.

Supporting Information

## Data Availability

The data that support the findings of this study are available in the supplementary material of this article.
