# Supplementary material for: Uncovering the Mechanism of Azepino‐Indole Skeleton Formation via Pictet–Spengler Reaction by Strictosidine Synthase: A Quantum Chemical Investigation
Source: ChemistryOpen. 2023 May 30;12(6):e202300043. doi: 10.1002/open.202300043 (PMC10233217; doi:10.1002/open.202300043)
Supplement: Supplementary file 1 — Supporting Information [file OPEN-12-e202300043-s001.pdf]

ChemistryOpen

Supporting Information

Uncovering the Mechanism of Azepino-Indole Skeleton Formation via Pictet–Spengler Reaction by Strictosidine Synthase: A Quantum Chemical Investigation

Mingqi Mou[+], Chenghua Zhang[+], Shiqing Zhang, Fuqiang Chen, Hao Su,* and Xiang Sheng*

Table of contents

1. Structures and energies of the optimized enzyme-substrate complexes S2
2. Structure of the transition state starting from E:IEA:SL S5
3. Optimized structure of the intermediates and product S5
4. Absolute energies and corrections for stationary points S7
5. Cartesian coordinates S8

1. Structures and energies of the optimized enzyme-substrate complexes

Optimized structures of the ES complexes of STR in complex with 1*H*-indole-4-ethylamine (4-IEA) and secologanin (SL) are shown in the figures below. Energies relative to the lowest-energy one (**E:IEA:SL**) are given in kcal/mol.

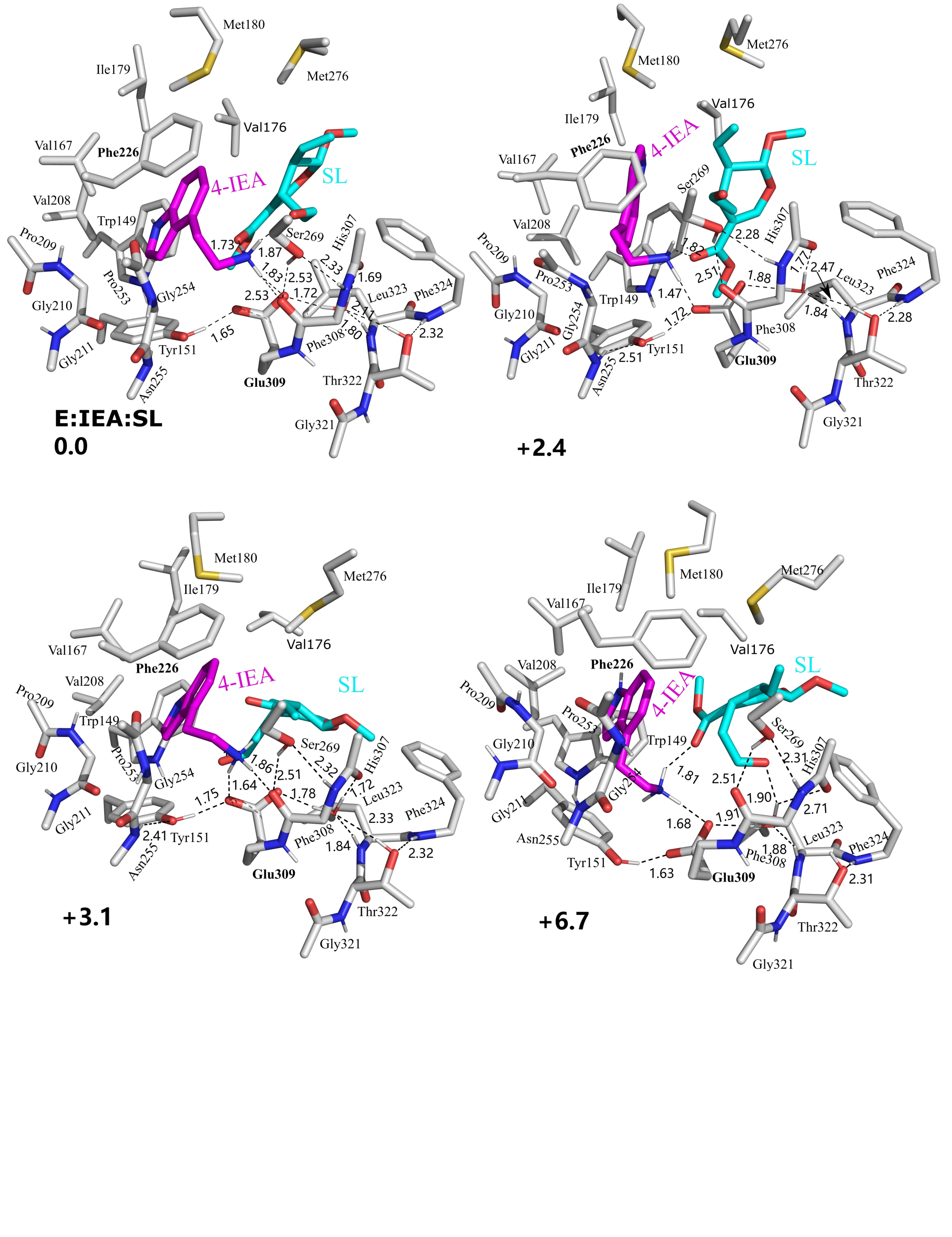

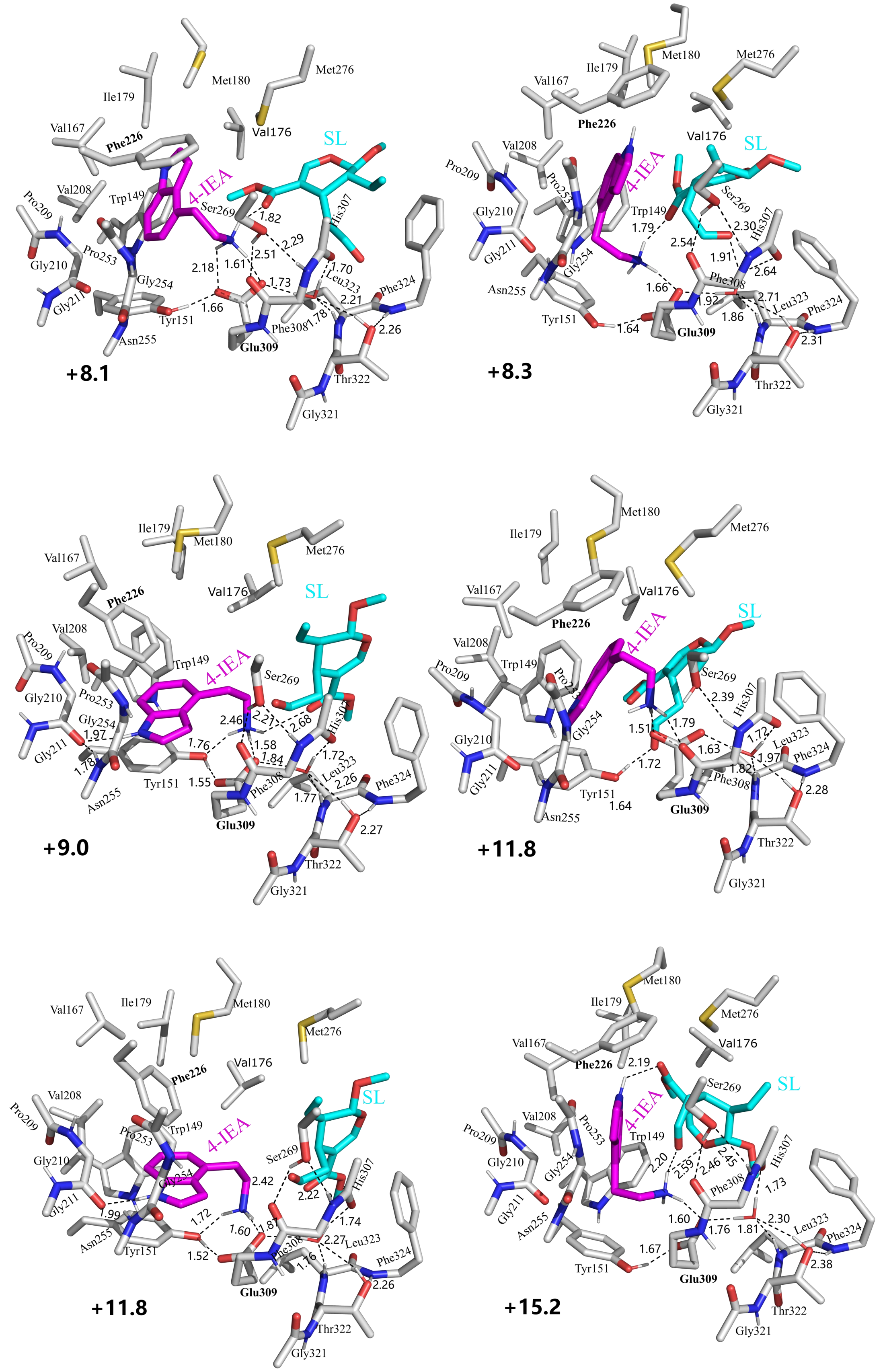
4-IEA
SL
Met180
Met276
Ile179
Val167
Val176
Phe226
Val208
Pro209
Trp149
Ser269
His307
Gly210
Pro253
Gly254
Gly211
Tyr151
Asn255
Phe308
Glu309
Leu323
Phe324
Thr322
Gly321
+8.1
+8.3
+9.0
+11.8
+11.8
+15.2

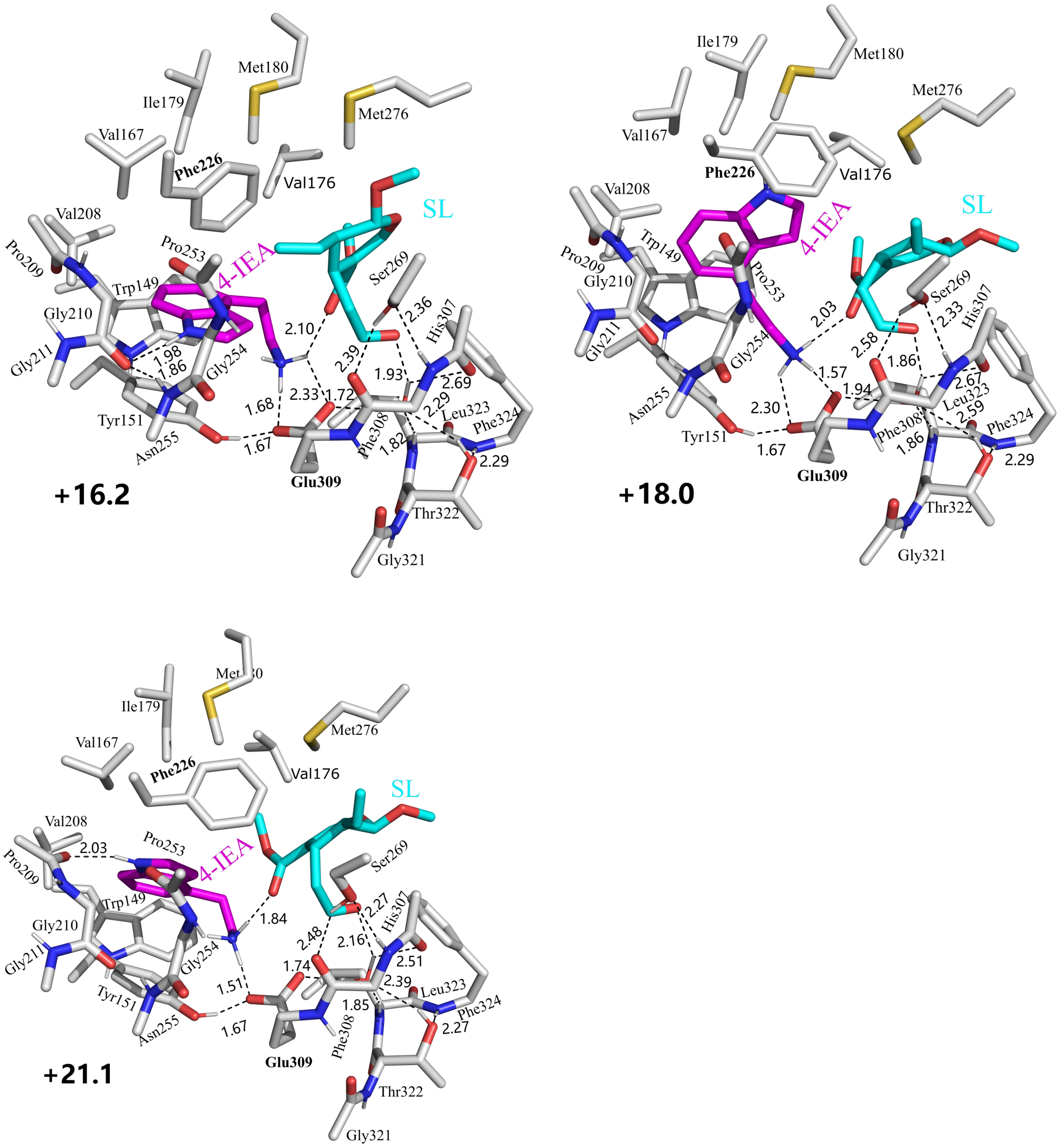
+16.2
+18.0
+21.1
4-IEA
SL
Phe226
Glu309
Met180
Met276
Ile179
Val167
Val176
Val208
Pro209
Gly210
Gly211
Trp149
Tyr151
Pro253
Gly254
Asn255
Ser269
His307
Phe308
Leu323
Phe324
Thr322
Gly321

2. Structure of the transition state starting from E:IEA:SL

Optimized structure of the transition state starting from the **E:IEA:SL**. The energy of this step is much higher than that of TS1 reported in the main text, which indicates that the pathway starting from **E:IEA:SL** is not favorable. Energies relative to **E:IEA:SL** are given in kcal/mol.

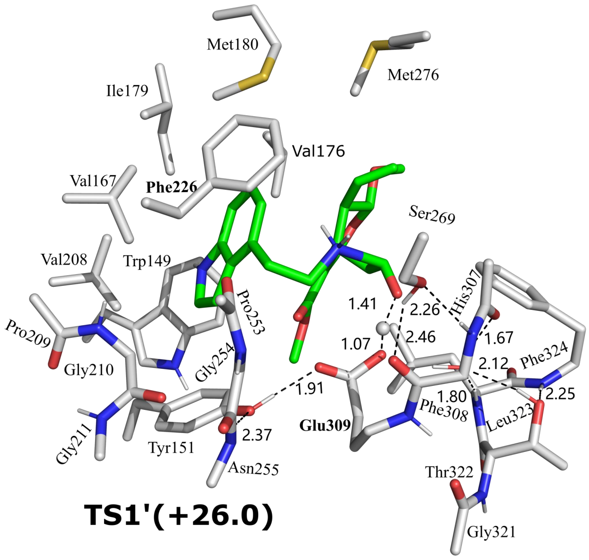

3. Optimized structure of the intermediates and product

Optimized structures of the intermediates and product in the lowest-energy pathway for the reaction of the 1*H*-indole-4-ethylamine (4-IEA) and secologanin (SL) are shown in the figures below. Energies relative to **E:IEA:SL** are given in kcal/mol.

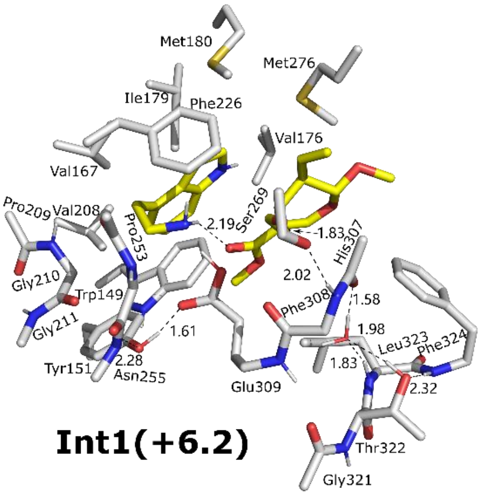

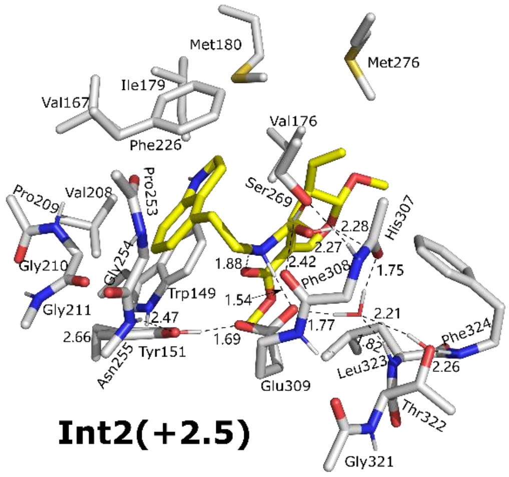

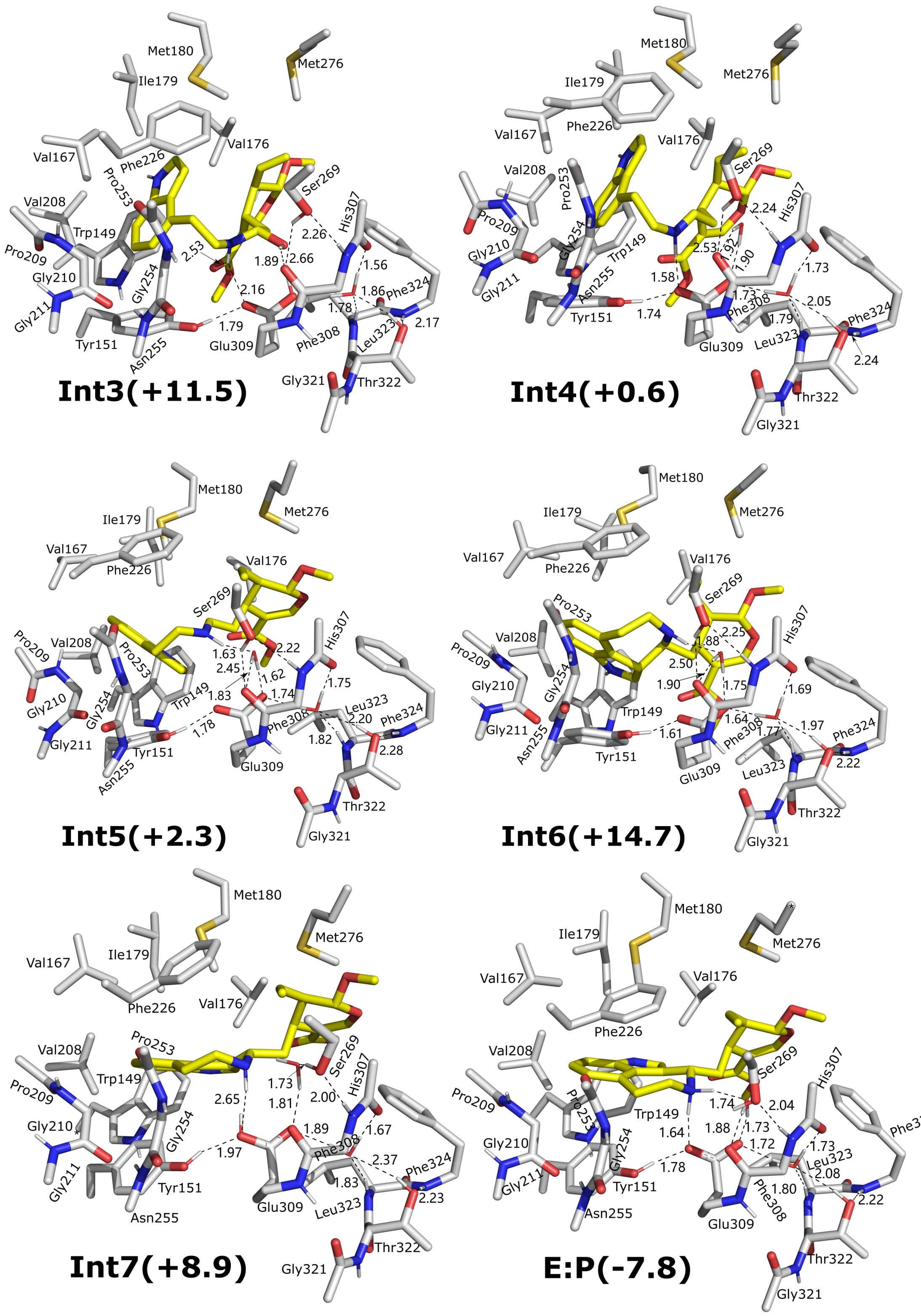
Int3(+11.5)
Int4(+0.6)
Int5(+2.3)
Int6(+14.7)
Int7(+8.9)
E:P(-7.8)

4. Absolute energies and corrections for stationary points

Table S1. Calculated absolute energies and energy corrections for the corresponding intermediates and transition states in the reaction of the 1*H*-indole-4-ethylamine (4-IEA) and secologanin (SL).

	E_{el} (a.u.)[a]	E_{solv} (a.u.)[b]	E_{bb} (a.u.)[c]	ZPE (a.u.)[d]	E_{tot} (a.u.)[e]	Δ E(kcal/mol)[f]
**E:IEA:SL**	-7382.12946	-7382.15912	-7384.01419	2.98385	-7381.06001	0.0
**E:IEA:SL'**	-7382.11287	-7382.14819	-7384.00159	2.98069	-7381.05621	2.4
**Int1**	-7382.12359	-7382.15004	-7384.00496	2.98123	-7381.05018	6.2
**TS1**	-7382.09816	-7382.12583	-7383.98551	2.98132	-7381.03188	17.7
**Int2**	-7382.12395	-7382.15326	-7384.01139	2.98462	-7381.05609	2.5
**Int3**	-7382.11174	-7382.14344	-7383.99519	2.98527	-7381.04162	11.5
**TS2**	-7382.09860	-7382.1312	-7383.98300	2.97932	-7381.03628	14.9
**Int4**	-7382.12353	-7382.15635	-7384.01069	2.98451	-7381.05900	0.6
**Int5**	-7382.12083	-7382.14960	-7384.00955	2.98200	-7381.05631	2.3
**TS3**	-7382.09474	-7382.12974	-7383.98234	2.98275	-7381.03459	16.0
**Int6**	-7382.09629	-7382.13267	-7383.98380	2.98367	-7381.03652	14.7
**TS4**	-7382.09481	-7382.12230	-7383.97877	2.98003	-7381.02623	21.2
**Int7**	-7382.11712	-7382.14498	-7384.00279	2.98484	-7381.04581	8.9
**E:P**	-7382.14227	-7382.17200	-7384.02876	2.98604	-7381.07246	-7.8

[a] Electronic energy calculated at the level of B3LYP-D3(BJ)/6-31G(d,p)

[b] Single point energy calculated using SMD solvation model with ε=4.0 at the same level of B3LYP-D3(BJ)/6-31G(d,p) level

[c] Single point energy calculated at the level of B3LYP-D3(BJ)/6-311+G(2d,2p)

[d] Zero point energy correction calculated at the level of B3LYP-D3(BJ)/6-31G(d,p)

[e] The total energy is obtained using $E_{tot} = E_{bb} + (E_{solv} - E_{el}) + ZPE$

[f] The energy relative to **E:IEA:SL**

5. Cartesian coordinates

The intermediates and transition states in the reaction of the 1-*H*-indol-4-ethanamine and secologanin substrates.

E:IEA:SL (0.0)

C	17.59400100	43.88503600	-6.44712600
C	18.91192700	44.43914500	-5.89323600
C	19.91650100	43.39072600	-5.50558800
C	19.75643000	42.02733000	-5.52464400
C	21.26082900	43.63143100	-5.03143600
C	21.85898200	42.36270500	-4.78079500
C	22.00695900	44.79133300	-4.76895800
N	20.91976500	41.40479800	-5.09720700
C	23.16070000	42.23228000	-4.28925100
C	23.30102400	44.66707400	-4.27421700
C	23.87680400	43.40007700	-4.04395800
H	17.76746000	43.27786400	-7.34176700
H	18.70072600	45.07370800	-5.02107600
H	19.36380900	45.10877700	-6.63751800
H	18.90050600	41.43636300	-5.81361200
H	21.57807300	45.77382600	-4.93958200
H	21.04260400	40.41086600	-5.00345300
H	23.59400500	41.25804800	-4.09512700
H	23.88403700	45.55611600	-4.06429400
H	24.90016700	43.33479500	-3.68866500
C	19.01603400	39.17801600	-2.73298400
C	20.00196500	40.05223700	-1.94131600
C	21.22518500	39.33617700	-1.40650100
C	22.39238100	39.19854100	-2.16968300
C	21.25628500	38.82494000	-0.10116400
C	23.56061100	38.63740300	-1.65141600
C	22.40558100	38.24671900	0.43125800
C	23.58381300	38.18220100	-0.32428700
O	24.69691800	37.69427300	0.27898600
H	19.50596200	38.71373800	-3.59772800
H	19.47269100	40.51795800	-1.10232300
H	20.32897300	40.87470000	-2.58271200
H	22.39963000	39.57011800	-3.19055400
H	20.36604000	38.90254600	0.51947400
H	24.46382700	38.57303200	-2.24941600
H	22.42587100	37.88764200	1.45163400
H	25.50574600	37.84867800	-0.28711100
C	18.62694300	45.31702100	-1.30413000
C	20.01062000	45.04649200	-1.90080100
C	21.06546900	45.97520200	-1.29520100
C	20.39857400	43.57859600	-1.71021100
H	18.61773700	45.08305600	-0.23264700
H	19.96299400	45.24551700	-2.97840100
H	22.04775600	45.80198100	-1.74884400
H	20.80954600	47.03133000	-1.43873800
H	21.15419100	45.79492400	-0.21972300
H	21.37760300	43.36719700	-2.15057000
H	20.42242900	43.31138800	-0.64697300
H	19.67544900	42.92338500	-2.20254600
C	27.28151700	48.52806500	-5.54729600
C	27.70098300	47.12906800	-5.06378600
C	27.67416100	47.02766800	-3.53260100
C	26.82472500	46.03876500	-5.69326400
H	26.24870400	48.74546500	-5.25165300
H	28.73689100	46.95444600	-5.38837900
H	26.66749100	47.22906500	-3.14565700
H	27.95627600	46.02371500	-3.19572600
H	28.35880000	47.74141600	-3.06405100
H	25.77246100	46.17140300	-5.41807600
H	26.88324000	46.05873300	-6.78644300
H	27.12830200	45.04124300	-5.35694800
C	24.77591200	51.75311100	-2.65200700
C	24.55616700	50.23695100	-2.59486100
C	23.76782300	49.76927700	-3.83081500
C	23.86851700	49.83689300	-1.28493900
C	23.55209800	48.25762200	-3.92019600
H	23.81358900	52.27897000	-2.69145900
H	25.54243000	49.74846800	-2.62449900
H	24.29447400	50.11351100	-4.73058000
H	22.79346800	50.27749700	-3.83765600
H	22.84933100	50.24046400	-1.24346000
H	23.80176900	48.75143000	-1.16807900
H	24.41305300	50.22673700	-0.41990900
H	23.06663100	47.98231700	-4.86193300
H	24.50724900	47.72394800	-3.87861700
H	22.92632000	47.88261400	-3.10511800
C	27.87202900	52.02402400	-0.48601900
C	29.25370700	51.39408400	-0.67631900
C	29.27258100	49.94210000	-1.16741400
S	28.90634900	48.61248400	0.05447000
C	27.08412900	48.48732200	0.00409500

H	27.31434500	51.53193500	0.31736700	H	25.28916900	44.25706400	6.81588400
H	29.82912900	51.45650600	0.25350400	H	24.48204800	45.13357500	3.98975700
H	29.80989900	51.98148000	-1.41920200	H	24.97140700	43.51004300	4.48051000
H	30.28872100	49.70803000	-1.49051000	H	27.34126400	43.16205400	5.33405400
H	28.60960600	49.80367700	-2.02898900	H	26.12638900	46.76679800	3.36715100
H	26.60184700	49.35374700	0.45736500	H	29.72556600	43.72945400	4.90322200
H	26.73557400	48.36993900	-1.02385700	H	28.49646300	47.33973000	2.91927700
H	26.81500800	47.59243700	0.57000000	H	30.30636400	45.82065600	3.68513900
C	21.29402600	46.95205600	2.73906400	C	26.40698900	40.65016000	8.06093600
C	22.81900100	47.10558200	2.61491200	C	26.07564100	40.57073500	6.56350300
C	23.42110600	46.05058400	1.67810500	O	25.73495200	41.56046100	5.91374900
C	23.19731300	48.51732500	2.15311100	H	27.00916100	39.80613300	8.40448700
H	20.80518300	47.08999900	1.76726500	N	26.18402500	39.32531800	6.01926700
H	23.24897100	46.95594400	3.61587200	C	25.51922500	38.92340500	4.78156500
H	24.51431800	46.07900900	1.68446500	C	24.77707500	37.61162800	5.12148900
H	23.12155600	45.03462700	1.95688800	O	24.66467200	37.25689200	6.29663800
H	23.09351300	46.21578200	0.64761400	H	26.23697700	38.74822600	3.97310800
H	24.28438600	48.63089300	2.07181100	H	24.81013600	39.69080500	4.46133000
H	22.76919500	48.72754100	1.16652400	H	26.17274000	38.56169400	6.68985200
H	22.82968600	49.28021400	2.84753800	N	24.31213900	36.88086200	4.08478200
C	19.41405500	43.82089900	3.87591800	C	23.31807100	35.84509700	4.34293000
C	19.93528900	42.71997100	2.99397900	H	22.30820200	36.25962700	4.28021500
O	19.45292300	42.44887900	1.89917100	H	24.25540200	37.34648800	3.18702400
H	19.94660900	43.91533400	4.82549200	C	32.73987800	40.47098800	6.36302000
N	20.97179700	42.02511100	3.54308200	C	31.85038900	40.39778700	5.11809700
C	21.66276200	40.99195600	2.82118000	O	31.96074400	39.10588100	4.49878100
C	21.53237700	39.61756600	3.45193300	H	33.78710600	40.29322500	6.10165900
O	22.49335600	38.85193700	3.52506900	H	32.13064000	41.18601200	4.40316200
H	22.72662900	41.21653400	2.73393200	H	30.80638900	40.58153700	5.40939200
H	21.24493900	40.96082600	1.81050000	H	31.19853400	38.98391000	3.90981000
H	21.37084100	42.34603100	4.41193700	C	34.70901200	47.89972800	1.13603300
N	20.30061000	39.27643100	3.89011200	C	33.60955500	48.39159900	0.18350900
C	20.04511400	37.96014900	4.44692600	C	34.19600700	48.91223800	-1.12994800
H	20.78918000	37.72817500	5.21278000	S	32.99842100	49.67858500	-2.29201700
H	20.09468800	37.18314100	3.67629600	C	32.07270000	48.22066800	-2.88385500
H	19.54634100	39.93594200	3.77415700	H	34.27880700	47.51265400	2.06738200
C	24.72096800	44.88215800	6.12209900	H	33.01617200	49.18831500	0.64297600
C	25.12967700	44.57600100	4.67106900	H	32.92426200	47.56493600	-0.03034800
C	26.57271300	44.92381000	4.38233100	H	34.72987300	48.11613900	-1.65861500
C	27.60436800	44.07844200	4.81462300	H	34.92114500	49.70917300	-0.93033600
C	26.91562100	46.10004300	3.70491700	H	31.43958500	48.56453300	-3.70491300
C	28.93856500	44.40021900	4.56805700	H	32.74038000	47.44386800	-3.25719700
C	28.24983200	46.42407600	3.45003600	H	31.43643300	47.80014300	-2.10404300
C	29.26718500	45.57214400	3.88122400	C	34.00191300	39.69602200	1.51297700

C	33.31335900	38.51027600	0.88355600
O	33.32712200	38.36154100	-0.34846900
H	34.05606400	39.63182600	2.60139700
N	32.72187400	37.65065400	1.74063900
C	31.89709400	36.54098300	1.30814900
C	30.40820800	36.83981100	1.45218700
O	29.99638200	37.85092300	2.04013900
H	32.11466400	36.32750800	0.26252600
H	32.63993700	37.95358300	2.70524600
N	29.63503300	35.88196600	0.91884200
C	28.19146200	35.92373500	0.98319300
C	27.50701400	35.43115500	-0.29192900
C	27.93588900	36.19955400	-1.55372200
C	27.96904500	37.69500600	-1.26309300
O	26.88586300	38.29930300	-1.02639800
O	29.10656900	38.26387200	-1.19542300
H	27.90363700	36.95642700	1.18860000
H	26.42926700	35.54822200	-0.13889200
H	27.70772500	34.36384600	-0.43035600
H	27.22432400	36.00017400	-2.35982000
H	28.92472400	35.87585600	-1.88321500
H	30.05823600	35.06210100	0.48484900
C	29.29895900	31.23689000	-1.09701000
C	30.11742600	32.50647200	-1.22857700
O	30.25160500	33.27226900	-0.26480400
H	28.25567300	31.50692500	-0.91020900
H	29.34977500	30.59661700	-1.98076800
N	30.69151800	32.74172300	-2.43193200
C	31.41898700	33.96301400	-2.68112900
C	31.17722900	34.43926000	-4.12196000
O	30.88845300	33.63852000	-5.01305700
C	32.94259000	33.81412400	-2.39510500
C	33.63803200	32.82409900	-3.31499600
O	33.60698300	35.06991500	-2.53868600
H	31.04764900	34.71802300	-1.98421200
H	33.01749300	33.46651200	-1.35367700
H	30.46478300	32.19533100	-3.25466400
H	34.69946200	32.76649400	-3.05999900
H	33.19932000	31.82873800	-3.20627500
H	33.54409800	33.13095200	-4.36012200
H	33.07142700	35.75008600	-2.09872200
N	31.38541100	35.76587800	-4.28698600
C	31.38114100	36.46303500	-5.55957900
C	32.71178900	36.43320000	-6.33338800
O	32.74103200	36.90254800	-7.46855300
C	30.95912400	37.93474300	-5.36388700
C	29.47043800	38.22276800	-5.61403400
C	28.54609100	37.33457900	-4.77322100
C	29.18269000	39.70604900	-5.35653700
H	30.67277100	35.95805300	-6.22517200
H	31.54704000	38.54901500	-6.04653200
H	31.23179200	38.24490300	-4.34906800
H	29.27697100	38.01736800	-6.67604700
H	27.49326000	37.56767500	-4.96840500
H	28.69824500	36.27272700	-4.98618300
H	28.73891800	37.49518300	-3.71001400
H	31.50064300	36.33999500	-3.44867500
H	28.14013800	39.95068700	-5.58045200
H	29.36188200	39.95959400	-4.30481300
H	29.82336600	40.34838000	-5.97103800
N	33.80472100	35.92055200	-5.70466500
C	35.12984000	36.23789400	-6.22531600
C	35.82617700	37.27095200	-5.30757300
C	34.92997600	38.44592600	-4.96910900
C	34.55328400	39.37009000	-5.95339800
C	34.41032400	38.60191400	-3.67770400
C	33.67492000	40.41126800	-5.65712900
C	33.53487600	39.64601800	-3.37215100
C	33.15843600	40.54814100	-4.36672900
H	34.97482600	36.63770300	-7.22809200
H	36.74616200	37.61218900	-5.79840200
H	36.12753100	36.77541700	-4.37773700
H	34.92648000	39.25435400	-6.96669100
H	34.67998300	37.89421400	-2.89885800
H	33.37725800	41.10384900	-6.43926000
H	33.14964900	39.72662700	-2.36093400
H	32.43752500	41.33203300	-4.15649500
H	33.71551700	35.66982200	-4.72326400
C	31.68437200	44.17758400	-3.61290000
O	32.49065900	45.14463000	-4.16792300
C	31.00404500	44.68140500	-2.34255900
C	30.10155500	43.54760800	-1.77591300
C	29.27305000	42.97254200	-2.90105300
C	29.59559100	43.17356000	-4.19470000
O	30.68945800	43.82535200	-4.61673200
C	31.94031200	45.20223900	-1.28164100
C	33.25364800	45.00190600	-1.19480100
C	30.88859600	42.44171200	-1.01173600

C	30.99724000	42.70694400	0.46867800
C	28.11408400	42.16915800	-2.51064600
O	27.91406700	41.86321900	-1.34327400
O	27.30511300	41.77966900	-3.50426600
C	26.26718900	40.84909900	-3.10233700
O	30.89670500	41.85480000	1.33561500
C	33.28431300	44.67846500	-5.26243100
H	30.33470600	45.49759300	-2.64730100
H	29.39984100	44.00362800	-1.07002700
H	32.25488700	43.25403400	-3.42655600
H	28.98426900	42.81352200	-5.01392400
H	31.42970400	45.76206600	-0.49674900
H	33.82197300	45.38880600	-0.35659200
H	33.81466400	44.47547800	-1.95899700
H	31.91995200	42.38853900	-1.38766200
H	30.44517200	41.45695400	-1.16458500
H	31.19159100	43.75600600	0.75867800
H	25.55297500	41.33715300	-2.43703000
H	25.79502800	40.54860300	-4.03616200
H	26.69915600	39.99591500	-2.57701100
H	32.66067500	44.45141000	-6.13146200
C	27.54240400	40.01533900	1.73767200
N	28.59651400	40.19555000	0.70019300
C	27.25551200	41.34000500	2.46511400
N	24.08128300	43.08367500	-0.68570000
C	26.82914900	42.45930000	1.53853700
C	27.62798400	43.58247600	1.34745300
C	27.26913600	44.61418200	0.45613800
C	26.09913000	44.54603600	-0.29125700
C	25.28426900	43.42569000	-0.09980000
C	23.65663000	41.86635200	-0.19143100
C	24.57010900	41.40280200	0.72436500
C	25.62460300	42.37721900	0.80579500
H	26.66558300	39.63612400	1.21251000
H	28.30157800	40.92570700	0.03118500
H	29.49714500	40.47611600	1.10508100
H	28.14692000	41.65005200	3.02007300
H	26.47910900	41.15118400	3.21520900
H	23.61492800	43.59772600	-1.41896300
H	28.53662100	43.68594200	1.92922300
H	27.92027300	45.47785900	0.35833100
H	25.82129900	45.33678900	-0.98270500
H	22.74381900	41.41413400	-0.54742500
H	24.49059700	40.46432600	1.25066100
H	27.90523100	39.24778500	2.42246600
H	16.91286000	44.69684200	-6.71615900
H	17.08998500	43.25604200	-5.70570300
H	18.33317100	46.36569200	-1.42602200
H	17.86284300	44.69424900	-1.78200200
H	18.61974200	38.36637400	-2.11446300
H	18.17408900	39.77463400	-3.09895200
H	27.33988300	48.60822100	-6.63707600
H	27.92090400	49.30675200	-5.11648500
H	27.95875900	53.08215800	-0.22290600
H	27.27283800	51.95524200	-1.39676800
H	25.34492700	52.04078000	-3.54295800
H	25.31336000	52.11814900	-1.77515600
H	35.39829700	48.70933800	1.39985500
H	35.29896100	47.09866800	0.68083300
H	32.43560300	39.71829200	7.09576300
H	32.65825000	41.46230200	6.81822800
H	23.65627600	44.67387300	6.27900500
H	24.90292100	45.93324900	6.36995000
H	29.65571700	30.68264400	-0.22586300
H	35.74230200	35.33302300	-6.30344800
H	19.05107900	37.94919200	4.89631600
H	33.43364300	40.59324300	1.24867100
H	35.00286400	39.79159500	1.08751200
H	27.83376900	35.32653800	1.83334800
H	32.14070000	35.64856800	1.89506200
H	33.97651400	45.48455200	-5.50591200
H	33.85044400	43.77998600	-4.98109900
H	20.87174300	47.68419300	3.43516200
H	21.04259700	45.95280900	3.10059700
H	19.48240400	44.76922900	3.34060100
H	18.35431300	43.64321300	4.07976700
H	26.93343600	41.58540300	8.25769000
H	25.46776200	40.66412100	8.62281300
H	23.42136200	35.04161900	3.60911300
H	23.49126200	35.45162400	5.34317400
H	28.76749400	39.35107700	0.10167200
O	31.47382700	37.09337200	-1.81551000
H	32.13159800	37.67049100	-1.37095800
H	30.58399100	37.47564500	-1.59993500

E:IEA:SL' (2.4)

C	7.48112700	1.51148700	-8.18580700
C	6.95653300	0.40335000	-7.26339400

C	5.50762400	0.52192000	-6.87967000	H	5.61746700	2.68501800	-3.15075700
C	4.67169300	1.59835400	-7.03706400	H	6.37429700	2.44202400	-4.73719900
C	4.72928100	-0.49873200	-6.21424000	H	5.44116700	1.14243900	-3.99512700
C	3.42061000	0.02460900	-6.00711700	C	4.31769600	-6.65110300	-1.89628100
C	5.00965500	-1.80581400	-5.78196400	C	2.86463400	-6.20027500	-1.73089000
N	3.42405700	1.31689900	-6.49450400	C	2.58781200	-5.76198700	-0.28876000
C	2.39734600	-0.72733700	-5.42015800	C	2.51155000	-5.09243200	-2.73103700
C	3.99670300	-2.55821500	-5.19667600	H	5.00889900	-5.82970500	-1.67416100
C	2.70248500	-2.02569600	-5.02428800	H	2.21806700	-7.06209700	-1.94926900
H	6.91302600	1.54632300	-9.12082400	H	3.18799700	-4.88191300	-0.02556600
H	7.56553300	0.37878800	-6.34811000	H	1.53812000	-5.49742900	-0.15057400
H	7.11359300	-0.57083800	-7.74512900	H	2.83621200	-6.55467600	0.42469500
H	4.86377600	2.56446800	-7.47938100	H	3.08474100	-4.17890300	-2.52507700
H	6.00189400	-2.22704300	-5.91454300	H	2.74001800	-5.39662900	-3.75861000
H	2.61601000	1.91148500	-6.57300600	H	1.44952300	-4.84042900	-2.68438400
H	1.40388400	-0.31796700	-5.26580300	C	8.69676800	-5.78471500	0.36828300
H	4.20027600	-3.57187200	-4.86707700	C	7.66146700	-4.74890600	-0.10108200
H	1.93509200	-2.63446600	-4.55694100	C	7.94361200	-4.33935200	-1.55958700
C	3.55997700	5.54690500	-5.67401200	C	7.63605500	-3.54088600	0.84373800
C	3.84789600	5.38229500	-4.17090900	C	6.90645400	-3.40882900	-2.19622600
C	2.61135700	5.45683100	-3.30072800	H	9.70760300	-5.36100900	0.33069400
C	1.71450600	4.38423400	-3.23366800	H	6.66948100	-5.22503300	-0.06962100
C	2.31247900	6.58969900	-2.53074000	H	8.02404100	-5.24877800	-2.16887200
C	0.57416400	4.41920500	-2.43132000	H	8.93057700	-3.85774000	-1.60053700
C	1.17632300	6.64960500	-1.72530400	H	8.60994500	-3.03788000	0.85386700
C	0.30039700	5.55971800	-1.66274300	H	6.88677800	-2.79929900	0.55389800
O	-0.77539200	5.63950400	-0.83049300	H	7.40312800	-3.84659200	1.86824500
H	2.87018400	4.77040900	-6.02423400	H	7.16317300	-3.17843600	-3.23498100
H	4.56156100	6.14951000	-3.85120500	H	5.91548000	-3.88005200	-2.21667400
H	4.34371300	4.41737600	-4.01303000	H	6.82713500	-2.45536200	-1.66631800
H	1.92851200	3.48057400	-3.80080400	C	7.03608000	-6.67526100	3.65314100
H	2.99086400	7.43850500	-2.54817400	C	5.87993900	-6.75956200	4.65372700
H	-0.07821700	3.55714900	-2.36970100	C	4.58643900	-6.08953400	4.18438700
H	0.96468500	7.52294000	-1.12025900	S	4.79558000	-4.27467000	3.94972600
H	-1.34632600	4.83793100	-0.93003300	C	3.03756300	-3.77989400	3.94381600
C	8.41940500	2.58416600	-2.93816100	H	7.34600700	-5.63855200	3.49124200
C	7.40230300	1.45006300	-3.09154300	H	6.18345600	-6.32068900	5.61124300
C	7.07558900	0.81189700	-1.73610500	H	5.64722000	-7.81361200	4.85148000
C	6.13572800	1.95703800	-3.78575200	H	3.80200600	-6.22717900	4.93587200
H	8.00586500	3.39106000	-2.32024700	H	4.23387000	-6.53092800	3.24694000
H	7.85149900	0.67467100	-3.73014900	H	2.48654400	-4.28961000	3.15174100
H	6.36954600	-0.01593800	-1.84325200	H	2.57076400	-4.00316800	4.90732000
H	7.97911900	0.43209500	-1.24558800	H	3.00463800	-2.70310600	3.77410600
H	6.61072600	1.54369500	-1.06454000	C	8.25713200	2.01909300	2.13965200

C	7.97659400	0.61322900	2.68451100	C	1.01248700	5.63027200	5.69481500
C	6.64816800	0.06659900	2.15172000	O	2.12531600	5.58295200	5.17329100
C	9.13119200	-0.34091200	2.35840300	H	-0.17413200	5.88314500	7.53343900
H	8.34395900	2.00010300	1.04645900	N	-0.15189500	5.51466300	4.97996800
H	7.89847300	0.68753300	3.77927100	C	-0.17207300	5.68954100	3.53904700
H	6.41925700	-0.91498600	2.57748000	C	-0.63822000	7.12214600	3.17903800
H	5.81165900	0.73177800	2.38612600	O	-0.76279000	7.97162900	4.05673000
H	6.67647400	-0.04718200	1.06253900	H	-0.81938500	4.94276900	3.06437700
H	8.95079100	-1.33798200	2.77247900	H	0.84672000	5.57716200	3.16225900
H	9.24533700	-0.44857600	1.27280800	H	-0.97837000	5.85908500	5.45002800
H	10.08193400	0.02667400	2.75938200	N	-0.89894300	7.34758000	1.86471500
C	7.42018200	5.68340300	1.43080600	C	-0.93789200	8.71670300	1.35480400
C	6.11752900	6.07117100	0.78489800	H	0.06657400	9.14874000	1.30933800
O	6.05109200	6.75212000	-0.24171200	H	-0.57822900	6.65397600	1.19760500
H	7.30886800	5.40133800	2.48082600	C	-3.80010000	1.28267400	8.48240500
N	5.01096900	5.56390800	1.38133900	C	-3.52782400	1.93258200	7.12370000
C	3.71966800	5.64001400	0.73773600	O	-4.34968500	1.34095800	6.10891200
C	2.93539300	6.93114400	0.95709300	H	-3.57535400	0.21274600	8.44873400
O	1.82647700	6.91122400	1.48985300	H	-2.46604700	1.80721400	6.86556300
H	3.09418100	4.83743200	1.12246000	H	-3.72265200	3.01400100	7.18807800
H	3.85607100	5.49500600	-0.33919400	H	-4.07356000	1.69026400	5.24395700
H	5.08275100	5.09880400	2.27311900	C	-0.41355900	-7.22762500	6.90830000
N	3.51671600	8.05649600	0.49338100	C	-0.10187000	-6.63684500	5.52745000
C	2.83292700	9.33421200	0.58904100	C	0.72783300	-7.58902100	4.66327200
H	2.39998700	9.44717000	1.58517900	S	1.17097100	-6.94123100	3.00421300
H	2.02393300	9.41811800	-0.14616100	C	-0.42911100	-7.09298200	2.14038700
H	4.41165100	7.96124100	0.02108100	H	-1.00339700	-6.53022600	7.51050600
C	4.82747900	3.03684900	5.97043700	H	0.44330000	-5.69199200	5.63550800
C	4.54223700	2.17637900	4.72947600	H	-1.03903800	-6.39506400	5.01259100
C	3.12970200	1.63287300	4.68244700	H	0.21773700	-8.55080000	4.53726400
C	2.04240800	2.50595600	4.53604300	H	1.68747700	-7.80035100	5.14729400
C	2.87261500	0.26021700	4.77676600	H	-0.29707400	-6.64506100	1.15396800
C	0.73716500	2.01765900	4.46506500	H	-0.71768200	-8.14172200	2.03287800
C	1.56703700	-0.23206300	4.68911500	H	-1.21577700	-6.54480000	2.66322000
C	0.49159600	0.64262700	4.52632900	C	-5.79756900	-1.40153200	4.67170800
H	4.15368900	3.89856700	6.00931700	C	-6.13913600	-0.52996900	3.47965500
H	5.25413100	1.34572400	4.67616100	O	-6.24986200	-0.99972700	2.33676200
H	4.71828300	2.78203200	3.82786600	H	-5.30817100	-0.83016800	5.46396900
H	2.21952500	3.57444900	4.47552100	N	-6.38170100	0.77372400	3.75496700
H	3.70344800	-0.42958500	4.90718000	C	-6.65796100	1.73416800	2.70851000
H	-0.08855900	2.71674600	4.36635800	C	-5.41288100	2.50901100	2.27437400
H	1.38727300	-1.30137800	4.74705300	O	-4.39719400	2.55285800	2.97346200
H	-0.51558600	0.25493100	4.42076400	H	-7.08196700	1.20692700	1.85540800
C	0.85660600	5.72301700	7.20856100	H	-6.10255900	1.12391500	4.66388400

N	-5.57561900	3.15237400	1.09946800
C	-4.61006200	4.12924700	0.63665200
C	-4.65947100	4.38833900	-0.86565700
C	-4.23554200	3.16613000	-1.69399400
C	-3.04664500	2.51209600	-1.00736500
O	-2.00528100	3.25946400	-0.84347800
O	-3.15549000	1.35147000	-0.56583900
H	-3.62134200	3.78901200	0.94317400
H	-3.97437400	5.21792900	-1.06604800
H	-5.66413500	4.71447100	-1.15239800
H	-3.96197900	3.47441200	-2.70903800
H	-5.04353800	2.43649500	-1.76748900
H	-6.49621000	3.17503500	0.66611600
C	-8.99185600	5.41937200	-1.94315000
C	-8.65294100	4.06384200	-1.35658200
O	-8.05665100	3.97737100	-0.27581900
H	-8.06068900	5.93715200	-2.19079800
H	-9.61632400	5.36060400	-2.83779100
N	-9.03153700	2.97130100	-2.06435200
C	-8.66370000	1.64791000	-1.62217800
C	-8.35687200	0.73811500	-2.81861200
O	-8.90390200	0.91524600	-3.90772100
C	-9.74692300	1.00381000	-0.70623500
C	-11.06558900	0.74925900	-1.41642900
O	-9.29690600	-0.25412000	-0.19518200
H	-7.76170300	1.74600700	-1.01076300
H	-9.89963500	1.71131200	0.12171200
H	-9.40888400	3.03490400	-3.00203500
H	-11.77951700	0.30129000	-0.72069100
H	-11.48363300	1.68822400	-1.78831600
H	-10.92650600	0.07399800	-2.26464100
H	-8.41201300	-0.14439700	0.18420500
N	-7.51215000	-0.27676500	-2.51615300
C	-7.17869500	-1.37055600	-3.41783100
C	-8.22627100	-2.49105100	-3.52723800
O	-8.07534400	-3.34612900	-4.39653400
C	-5.84154100	-2.02698300	-2.97784900
C	-4.57807000	-1.54271200	-3.71306400
C	-4.60373900	-1.92274900	-5.19836900
C	-4.30648800	-0.04757200	-3.52238200
H	-7.09778100	-0.97009200	-4.43239000
H	-5.92965500	-3.10399500	-3.13266600
H	-5.72021800	-1.87512500	-1.90040800
H	-3.74149700	-2.08881100	-3.25014100
H	-3.64592500	-1.68764200	-5.67628800
H	-4.80760000	-2.98922100	-5.33396000
H	-5.38186700	-1.37286900	-5.73849000
H	-6.98895300	-0.22188900	-1.64069700
H	-3.38691400	0.24226700	-4.04189000
H	-5.12517500	0.55765200	-3.92817000
H	-4.18328200	0.20444900	-2.46724300
N	-9.22956500	-2.51707200	-2.61094700
C	-9.98060100	-3.75197400	-2.40501400
C	-9.69865400	-4.32577800	-1.00231300
C	-8.21956700	-4.41759500	-0.69515700
C	-7.40584700	-5.35283500	-1.34795200
C	-7.62544400	-3.54265200	0.22187000
C	-6.04063100	-5.42527000	-1.07289200
C	-6.26010800	-3.61018300	0.50503000
C	-5.46760900	-4.55870000	-0.13987500
H	-9.65592700	-4.43658600	-3.19005300
H	-10.17063100	-5.31335100	-0.92943000
H	-10.18012000	-3.68908700	-0.25146400
H	-7.84449900	-6.02574600	-2.07940500
H	-8.23701200	-2.80003400	0.72734700
H	-5.42786700	-6.16277900	-1.58417400
H	-5.83926000	-2.92348000	1.23075900
H	-4.40889800	-4.62738300	0.09476300
H	-9.20650300	-1.83055800	-1.86207200
C	-0.83130800	-3.60714200	-1.30710600
O	-0.74213100	-4.96257500	-1.11045600
C	0.27847800	-2.82211700	-0.62533900
C	-0.09004200	-1.32276500	-0.81257100
C	-0.41405000	-1.08288600	-2.27356800
C	-0.68859600	-2.10017100	-3.11812900
O	-0.77608200	-3.38288300	-2.74650700
C	0.51510800	-3.15809400	0.82263000
C	-0.33342500	-3.75574900	1.65913800
C	-1.23803900	-0.89572900	0.11802000
C	-0.84505700	-0.69025100	1.55823500
C	-0.39863000	0.28487000	-2.77409300
O	-0.12528100	1.26226600	-2.08946600
O	-0.72452600	0.40670600	-4.08277000
C	-0.81734800	1.75167600	-4.57669100
O	-1.65035600	-0.54815200	2.45857300
C	-1.88809800	-5.63324500	-1.62211500
H	1.20371300	-3.01233300	-1.17898000
H	0.79998600	-0.73176600	-0.57111600

H	-1.82331000	-3.24373800	-0.99967600
H	-0.85532300	-1.96001000	-4.17924800
H	1.47536600	-2.81502000	1.19895800
H	-0.06757400	-3.91876500	2.69787700
H	-1.30515700	-4.12062800	1.34445600
H	-2.07734200	-1.59733300	0.09807100
H	-1.69371100	0.04441200	-0.22291100
H	0.24467400	-0.58635500	1.76205100
H	-1.50555200	2.33696000	-3.96420600
H	0.16183600	2.23527000	-4.56800400
H	-1.19226700	1.66090200	-5.59533600
H	-1.87695100	-5.65840400	-2.71516600
C	1.09274000	2.95665700	0.93422300
N	-0.10546700	2.17290900	0.52013400
C	2.27594600	2.03243400	1.23768400
N	4.12341100	-2.08464200	-0.38795000
C	2.75830300	1.20582900	0.06363500
C	2.75089400	1.69888600	-1.23760300
C	3.16516800	0.92144100	-2.33395300
C	3.63426400	-0.37340000	-2.16906400
C	3.68321900	-0.86416400	-0.86265700
C	4.00307200	-2.11609400	0.98872200
C	3.45555700	-0.93287000	1.42271700
C	3.24807600	-0.10603200	0.26320800
H	1.30582200	3.65717500	0.12654200
H	0.13979400	1.52023000	-0.24087500
H	-0.93166000	2.72421600	0.03728300
H	2.00027100	1.37411700	2.06511500
H	3.09280000	2.65153800	1.63031600
H	4.55997400	-2.79947800	-0.94702400
H	2.39389700	2.70162500	-1.43277000
H	3.11977500	1.34297900	-3.33130900
H	3.95557900	-0.96894500	-3.01399000
H	4.33907700	-2.97766100	1.54827400
H	3.25661100	-0.66364900	2.44727300
H	0.82448900	3.53623400	1.81792900
H	8.53348100	1.34573800	-8.43128300
H	7.40147500	2.49370200	-7.70735800
H	9.34178200	2.23721700	-2.45920600
H	8.68482200	3.01525500	-3.90942600
H	3.09590600	6.51573000	-5.88284900
H	4.48150600	5.47216500	-6.26068200
H	4.52321000	-6.98730000	-2.91844400
H	4.56039000	-7.47366100	-1.21494200
H	7.90702200	-7.23241100	4.01083500
H	6.74333500	-7.08941300	2.68243300
H	8.68482400	-6.67542200	-0.26891000
H	8.50714000	-6.10046700	1.39674900
H	0.50640500	-7.45111300	7.45950000
H	-0.98312400	-8.15925300	6.82132000
H	-4.84917300	1.40663500	8.76680200
H	-3.17324900	1.74913000	9.24790700
H	5.85828200	3.40593600	5.96015200
H	4.68524400	2.45189900	6.88456100
H	-9.50352300	6.01058700	-1.18065800
H	-11.05565300	-3.57692400	-2.52774400
H	3.55087400	10.13737500	0.41375400
H	-5.14485400	-2.20763900	4.33463000
H	-6.71777100	-1.84770900	5.06302500
H	-4.77579800	5.08432600	1.15748600
H	-7.40344200	2.45454500	3.06449800
H	-1.85939400	-6.65093200	-1.22989700
H	-2.81019600	-5.14093700	-1.28958900
H	9.18769400	2.43104100	2.54452500
H	7.44334200	2.70716200	2.39487700
H	7.83919200	4.82714900	0.89242800
H	8.12830000	6.50945900	1.34855900
H	1.22927100	4.79355700	7.64886800
H	1.48359700	6.54015800	7.57054400
H	-1.37361400	8.70366500	0.35360300
H	-1.55418400	9.32769600	2.01519000
H	-0.49499600	1.65614600	1.30882200
O	-5.91060700	0.20640700	-0.14678800
H	-5.88814400	-0.27085700	0.70735200
H	-5.02551900	0.60723400	-0.26479200

Int1 (6.2)

C	7.48101900	1.51141900	-8.18577700
C	6.80066100	0.47206500	-7.29033700
C	5.46332300	0.89617100	-6.76223100
C	4.86011600	2.12158400	-6.89815100
C	4.55081200	0.07992400	-5.99691300
C	3.40756700	0.87887500	-5.70681200
C	4.59007500	-1.24105900	-5.52134700
N	3.62365900	2.11595500	-6.27168400
C	2.32366200	0.39108500	-4.97295500
C	3.51416400	-1.72963100	-4.78824500
C	2.38980000	-0.92302900	-4.52125100

H	6.85892000	1.75015700	-9.05449200	H	4.31919800	-5.09032900	0.41528800
H	7.45866900	0.24413400	-6.44553400	H	2.66403200	-5.46601400	0.94541600
H	6.68634400	-0.46809700	-7.84814300	H	3.87377000	-6.73728600	0.85081900
H	5.20862900	3.01167400	-7.39914000	H	3.33109700	-4.04282600	-1.80568800
H	5.44986300	-1.87222400	-5.72490200	H	2.26992200	-5.02845400	-2.81394800
H	3.00736900	2.90878100	-6.20587100	H	1.68636000	-4.44790900	-1.25415900
H	1.46206800	1.01545600	-4.76689800	C	8.69704400	-5.78479100	0.36799100
H	3.53434300	-2.75011400	-4.41767400	C	7.68550000	-4.64055500	0.54657100
H	1.56445900	-1.33211800	-3.94729100	C	7.39379300	-3.94479000	-0.79605200
C	3.55991700	5.54694900	-5.67399900	C	8.16943400	-3.64488000	1.60955800
C	3.96560700	5.02152600	-4.27939900	C	6.31001200	-2.86365500	-0.74220200
C	2.88007800	5.19224000	-3.24648200	H	9.66180000	-5.39820400	0.01816600
C	2.24230000	4.08908100	-2.66628800	H	6.74085100	-5.07957000	0.90054400
C	2.45363700	6.46947300	-2.85734800	H	7.09979000	-4.70752200	-1.53008200
C	1.19470000	4.24031300	-1.75354500	H	8.32868100	-3.50818400	-1.17493100
C	1.40738200	6.64160900	-1.96145100	H	9.05441500	-3.10147100	1.25522600
C	0.75246800	5.52790700	-1.41688300	H	7.39781000	-2.91204500	1.85816500
O	-0.29804200	5.76353400	-0.59545300	H	8.44229100	-4.15257000	2.54130600
H	2.63956800	5.06397500	-6.02296100	H	6.10319500	-2.45898200	-1.73821900
H	4.87979100	5.53563900	-3.95191400	H	5.37057600	-3.27274300	-0.34869100
H	4.21552600	3.95607900	-4.34773500	H	6.58458800	-2.02238600	-0.10371600
H	2.56594300	3.08643200	-2.93590100	C	7.03588900	-6.67527400	3.65338400
H	2.94895100	7.34583000	-3.26758700	C	6.69624400	-6.39768700	5.11826300
H	0.72234800	3.37176600	-1.30397800	C	5.26819700	-5.89209200	5.33436800
H	1.07207500	7.63143800	-1.67068700	S	5.01935800	-4.23243500	4.58053500
H	-0.60759100	4.93065400	-0.15437100	C	3.41266700	-3.82885200	5.33841400
C	8.41937400	2.58415400	-2.93803800	H	6.92566200	-5.77963100	3.03757200
C	7.37408400	1.72632100	-3.67430500	H	7.40247600	-5.66916800	5.53327000
C	7.69013900	0.23387200	-3.54970100	H	6.81413400	-7.31706400	5.70639800
C	5.95541900	2.03048700	-3.17819300	H	5.06047600	-5.80587900	6.40613100
H	8.42815000	2.35134600	-1.86823200	H	4.54330000	-6.58691100	4.89423900
H	7.42264800	1.98410900	-4.74365200	H	2.62912200	-4.50822700	4.99306600
H	6.94505600	-0.36946000	-4.07783900	H	3.47914100	-3.85629800	6.43051400
H	8.67869900	-0.00422000	-3.95965500	H	3.16282700	-2.81469900	5.03033300
H	7.67943300	-0.07863300	-2.49892400	C	8.25708200	2.01914800	2.13972400
H	5.80386800	1.70800600	-2.14638400	C	6.84747100	1.52381800	1.81642100
H	5.78457500	3.11459700	-3.22832000	C	6.65576900	1.47861700	0.29761500
H	5.20888100	1.52811600	-3.79982800	C	6.56532100	0.15179000	2.45560400
C	4.31765600	-6.65103300	-1.89629900	H	9.00637900	1.31164100	1.76284100
C	3.12547500	-6.09798400	-1.10771400	H	6.12847700	2.24797100	2.22725200
C	3.51558300	-5.83062500	0.35317300	H	5.65625800	1.15025800	0.01999600
C	2.56939800	-4.83315800	-1.77905600	H	6.80857000	2.47103800	-0.13712600
H	5.12952400	-5.90151600	-1.90621300	H	7.37856900	0.79558400	-0.16923000
H	2.33148900	-6.85829000	-1.11491800	H	5.55391100	-0.17769600	2.19858900

H	7.27582800	-0.58724800	2.06800000
H	6.66988000	0.16509400	3.54504000
C	7.42020100	5.68340400	1.43081200
C	6.11612100	6.03342700	0.76924500
O	6.04810700	6.72747600	-0.24757300
H	7.30117600	5.12221300	2.35978100
N	5.01669200	5.49970100	1.35399900
C	3.71965700	5.64006300	0.73767600
C	2.99971200	6.95399900	1.03864000
O	2.00727700	6.98554900	1.76565900
H	3.06896400	4.84580500	1.09737600
H	3.83809700	5.53384800	-0.34499100
H	5.09020400	5.02477900	2.24000800
N	3.50635100	8.05559600	0.44644800
C	2.83289300	9.33419000	0.58906200
H	2.52459700	9.47441700	1.62702200
H	1.93636700	9.38655900	-0.04026200
H	4.33517600	7.94257300	-0.12841400
C	4.82750200	3.03684600	5.97045200
C	4.60493000	1.52420500	5.86124600
C	3.17746700	1.03673000	6.04036000
C	2.07786100	1.90150900	5.99232800
C	2.93406800	-0.33247300	6.23182900
C	0.77269300	1.40906200	6.10902800
C	1.63424600	-0.82681600	6.34385400
C	0.54185400	0.04290100	6.27628800
H	4.25133900	3.59341900	5.22675900
H	5.24018000	1.00381100	6.58974500
H	4.96145900	1.18700600	4.87805600
H	2.22480200	2.96611600	5.85320700
H	3.77747800	-1.01550500	6.29061100
H	-0.06386900	2.10061100	6.05647100
H	1.47272700	-1.89034700	6.49377100
H	-0.47221600	-0.33681800	6.35907300
C	0.85661300	5.72302400	7.20859100
C	0.94002100	5.40072500	5.71728700
O	1.96662600	4.91196400	5.22946500
H	-0.08497700	6.19543800	7.49675800
N	-0.17938100	5.64480900	4.99462500
C	-0.17205100	5.68954800	3.53904500
C	-0.78575800	7.05214500	3.14030900
O	-1.26752400	7.79557400	4.00057100
H	-0.73560200	4.86408100	3.09353600
H	0.85468100	5.64676200	3.17240600
H	-0.85363100	6.29998300	5.38213600
N	-0.75610800	7.35081100	1.82482500
C	-0.93783700	8.71671200	1.35481200
H	-0.04285000	9.32316500	1.53607900
H	-0.19126300	6.76079800	1.22377800
C	-3.80104200	1.28242000	8.48055000
C	-3.38774800	1.57655700	7.04303200
O	-4.49319600	1.39190300	6.15430600
H	-4.13812300	0.24659900	8.57989200
H	-2.57220400	0.90006000	6.76034700
H	-3.00844000	2.60694800	6.95674700
H	-4.12709200	1.38411300	5.25550600
C	-0.41351200	-7.22759700	6.90828900
C	0.09255300	-6.64960200	5.57989600
C	1.37090300	-7.34379500	5.10914000
S	2.05374800	-6.75620000	3.51115500
C	0.79009400	-7.39709100	2.35631700
H	-1.32282600	-6.71534400	7.23781900
H	0.28757700	-5.57431900	5.68019700
H	-0.68382200	-6.74060000	4.81228200
H	1.22245000	-8.42739300	5.03367700
H	2.17862400	-7.18248100	5.83153300
H	1.21805600	-7.32581600	1.35325100
H	0.58023300	-8.44940000	2.56746200
H	-0.12307600	-6.80303300	2.39656500
C	-5.79719600	-1.40157200	4.67200100
C	-5.94704300	-0.48870200	3.48438000
O	-5.75505100	-0.91534700	2.33080100
H	-5.45221500	-0.84920700	5.54985800
N	-6.38943000	0.75939500	3.74752000
C	-6.65727900	1.73472700	2.71062500
C	-5.41683600	2.50056500	2.26874100
O	-4.39347200	2.52771100	2.94102600
H	-7.13623300	1.24984800	1.86475300
H	-6.30237600	1.08969900	4.69981500
N	-5.58322000	3.14125400	1.07910200
C	-4.60999900	4.12882500	0.63623400
C	-3.63906800	3.57588000	-0.41475200
C	-2.81461100	2.39785700	0.10718100
C	-1.72132000	2.77977200	1.08396500
O	-0.93162100	3.70998700	0.86013100
O	-1.62868900	2.02738200	2.13618200
H	-4.05195700	4.47871200	1.50927900
H	-2.96776700	4.37743700	-0.74175100

H	-4.21023100	3.25157400	-1.29229900
H	-2.29244900	1.91092200	-0.72201700
H	-3.45556700	1.64371900	0.56697900
H	-6.52451200	3.24421500	0.69807400
C	-8.99196100	5.41946800	-1.94327500
C	-8.62601600	4.06849700	-1.36209300
O	-8.03551600	3.98206000	-0.27797000
H	-8.08824000	6.02972800	-2.01116300
H	-9.46180600	5.35337600	-2.92723200
N	-9.00545100	2.97700600	-2.07053000
C	-8.66376300	1.64793000	-1.62236100
C	-8.31406600	0.75997900	-2.82419800
O	-8.73983100	1.02173800	-3.94968100
C	-9.79207800	1.00804700	-0.75913800
C	-11.07286500	0.75590000	-1.53722200
O	-9.36423100	-0.23940700	-0.21580300
H	-7.79450900	1.74608100	-0.97260600
H	-9.98781000	1.72567300	0.05198700
H	-9.34497200	3.04337400	-3.02268700
H	-11.82041600	0.30610700	-0.87897000
H	-11.47406500	1.69398100	-1.92952300
H	-10.89292400	0.07894200	-2.37695500
H	-8.45736800	-0.12508300	0.12037800
N	-7.55942800	-0.31594300	-2.49958900
C	-7.17873400	-1.37068700	-3.41773400
C	-8.14746800	-2.56516500	-3.46603800
O	-7.91392400	-3.49072200	-4.23922500
C	-5.76221000	-1.89647100	-3.12304200
C	-4.57419900	-0.96602200	-3.45160900
C	-4.64735200	-0.40941500	-4.87821100
C	-4.35038300	0.16364400	-2.43658600
H	-7.21567500	-0.95047700	-4.42738100
H	-5.66142900	-2.80963700	-3.71443200
H	-5.71379100	-2.20194200	-2.07202700
H	-3.68429300	-1.60955700	-3.39338300
H	-3.71690700	0.10382700	-5.14393100
H	-4.81475200	-1.20639100	-5.61020900
H	-5.46171200	0.31607000	-4.98163900
H	-7.18260900	-0.37039600	-1.55620200
H	-3.42075000	0.69708900	-2.66108400
H	-5.16593600	0.89305700	-2.46530200
H	-4.27892200	-0.22832500	-1.41816200
N	-9.22857600	-2.52658100	-2.64098400
C	-9.98049500	-3.75188100	-2.40511500
C	-9.63154800	-4.34687400	-1.02125800
C	-8.13836100	-4.39241400	-0.77341000
C	-7.31221800	-5.24710000	-1.51533800
C	-7.54112000	-3.53223200	0.15575300
C	-5.93040700	-5.23654700	-1.33654900
C	-6.15719900	-3.52234300	0.34738200
C	-5.34718300	-4.37230200	-0.40668000
H	-9.70644400	-4.43576500	-3.20918100
H	-10.07008300	-5.35007900	-0.95096600
H	-10.10406500	-3.73876200	-0.24173300
H	-7.75270500	-5.90221200	-2.26065800
H	-8.16637200	-2.85255500	0.72918800
H	-5.30641500	-5.89338300	-1.93552600
H	-5.72713500	-2.84233200	1.07528300
H	-4.26842500	-4.34737000	-0.28784100
H	-9.25659300	-1.80157500	-1.92966500
C	-1.95399400	-4.08781300	1.68237600
O	-1.89282400	-5.39315500	2.12809500
C	-0.85854000	-3.23858600	2.32075800
C	-0.96527400	-1.79761600	1.75170000
C	-0.98909100	-1.86615100	0.24607100
C	-1.37852800	-2.99229500	-0.38388000
O	-1.78524900	-4.11900800	0.23273000
C	-0.84098500	-3.20710400	3.82211200
C	-1.76238800	-3.67742300	4.66025400
C	-2.17962500	-1.00648500	2.28321500
C	-2.00112800	-0.45010900	3.66732500
C	-0.58878400	-0.66151800	-0.49127600
O	-0.18623000	0.36086300	0.04928900
O	-0.73398700	-0.76916700	-1.83462800
C	-0.35388800	0.39638200	-2.58482700
O	-2.69192684	0.41852486	4.16123996
C	-2.98532700	-6.21293200	1.70683100
H	0.09046200	-3.64471100	1.95963900
H	-0.05271900	-1.26872300	2.04597900
H	-2.95596700	-3.66897900	1.85539200
H	-1.38950400	-3.09361800	-1.46271500
H	0.02368800	-2.68161200	4.22788300
H	-1.65926100	-3.54419000	5.73082900
H	-2.63788000	-4.21932400	4.32038200
H	-3.12206600	-1.55761100	2.22770700
H	-2.29291500	-0.11176200	1.66298600
H	-1.13722384	-0.85945200	4.22939216
H	-0.99120300	1.24494900	-2.32793100

H	0.68628400	0.65490500	-2.38244700
H	-0.49681700	0.12609700	-3.63086500
H	-2.94406400	-6.40558900	0.63127000
C	2.13432379	2.56134918	2.51750679
N	1.11696879	1.53935918	2.83392379
C	3.50950479	1.93462618	2.22724879
N	3.02969279	-2.64621182	0.89353079
C	3.44698979	0.90491518	1.12293479
C	3.52265479	1.28107018	-0.21721021
C	3.45811979	0.34115118	-1.26306221
C	3.29799779	-1.01088982	-1.00485321
C	3.19513979	-1.39671782	0.33508779
C	3.00391779	-2.54573882	2.26639479
C	3.13427279	-1.22505082	2.62909279
C	3.26569179	-0.46800682	1.40851579
H	1.77161979	3.11100318	1.64586179
H	1.17397479	0.78903618	2.13970979
H	-1.09774179	1.69057965	2.81911694
H	3.89370379	1.47436718	3.14392479
H	4.21254479	2.72710718	1.95015479
H	3.02434679	-3.50836082	0.37717379
H	3.66196379	2.33132218	-0.46199321
H	3.55581479	0.66601618	-2.29043821
H	3.26643079	-1.73013082	-1.81399921
H	2.91373979	-3.43772682	2.86710479
H	3.15052179	-0.84737182	3.63918379
H	2.19541179	3.25830018	3.35691079
H	8.44432500	1.14375100	-8.55024400
H	7.66606600	2.44138000	-7.63790100
H	9.42633000	2.40342700	-3.32894800
H	8.20153900	3.65284800	-3.04504400
H	3.35995000	6.62219000	-5.65422500
H	4.35100300	5.36812100	-6.41041100
H	4.04656200	-6.87269400	-2.93337500
H	4.70658400	-7.56821300	-1.44307800
H	8.06336300	-7.03535900	3.54464000
H	6.36465400	-7.43584600	3.23974200
H	8.34433000	-6.51604900	-0.36730800
H	8.87071500	-6.31018100	1.31164300
H	0.33672600	-7.11934000	7.69929700
H	-0.64547300	-8.29407600	6.81500100
H	-4.61927600	1.94048400	8.79323100
H	-2.95339700	1.44111000	9.15364800
H	5.88771300	3.26917600	5.83167400
H	4.52320100	3.41366700	6.95189400
H	-9.67574800	5.92142600	-1.25334900
H	-11.05638000	-3.55658700	-2.46796700
H	3.51545400	10.13537800	0.29973400
H	-5.08169200	-2.18296500	4.43245300
H	-6.77055500	-1.85700500	4.88491700
H	-5.16441000	4.97567300	0.22194600
H	-7.37273900	2.46462500	3.10824600
H	-2.89235300	-7.15229800	2.25313400
H	-3.94594500	-5.73990500	1.94571000
H	8.41472300	2.11942400	3.22073400
H	8.45998200	2.98948100	1.67760800
H	8.01124500	5.08362800	0.73346600
H	7.98448300	6.59810100	1.62952100
H	0.98137100	4.79580700	7.77546900
H	1.68912200	6.38201800	7.46616000
H	-1.14552900	8.69135300	0.28292000
H	-1.77844900	9.17248400	1.87986800
H	1.30501979	1.13409218	3.74953979
O	-6.48000300	0.09703000	0.07048600
H	-6.14906900	-0.32597400	0.91252700
H	-5.93789600	0.89068200	-0.04206400

TS1 (17.7)

C	7.48116300	1.51148300	-8.18582900
C	7.08935400	0.21452500	-7.46714800
C	5.61503300	0.04836500	-7.23329300
C	4.61874900	0.95792800	-7.48100500
C	4.96589800	-1.12244900	-6.68403300
C	3.56823700	-0.85465100	-6.63914500
C	5.42789700	-2.37048200	-6.23376300
N	3.38844100	0.43305600	-7.10538500
C	2.63783200	-1.79441300	-6.18785000
C	4.50629300	-3.31232400	-5.78641700
C	3.12551500	-3.02805800	-5.76989400
H	7.00019200	1.57946000	-9.16690300
H	7.61662100	0.16278400	-6.50451300
H	7.45458900	-0.64411100	-8.04702100
H	4.68201300	1.95713400	-7.88489600
H	6.48960700	-2.59837100	-6.24000900
H	2.49728000	0.85216000	-7.31007600
H	1.57877800	-1.56341600	-6.14570900
H	4.85288100	-4.28073100	-5.43874800
H	2.43034000	-3.77832700	-5.40827000

C	3.55994400	5.54694100	-5.67403500	C	7.76644200	-3.44109700	0.39492100
C	3.76286000	5.02011300	-4.24331700	C	7.28334200	-3.81809800	-2.71760300
C	2.49668700	5.06323000	-3.41890200	H	9.74070000	-5.45014200	0.34130000
C	1.50683300	4.08499400	-3.57756100	H	6.76849800	-5.18635200	-0.36106800
C	2.24853200	6.09248500	-2.50118100	H	8.39267700	-5.60494100	-2.26021100
C	0.32065300	4.11808400	-2.85048300	H	9.22675800	-4.12367400	-1.82569700
C	1.07239900	6.13548000	-1.75273800	H	8.77592800	-3.01811500	0.45638200
C	0.10555000	5.13715100	-1.91382400	H	7.12356300	-2.69988900	-0.08616700
O	-1.00722800	5.18054200	-1.12768000	H	7.39548600	-3.57509400	1.41639800
H	2.79587000	4.96201100	-6.19733900	H	7.69123600	-3.70135500	-3.72672500
H	4.54463700	5.60560200	-3.74615800	H	6.32433600	-4.33958400	-2.82383300
H	4.13336300	3.98914300	-4.29273300	H	7.08530800	-2.81550800	-2.32853800
H	1.66653500	3.25972600	-4.26562100	C	7.03608100	-6.67527600	3.65316400
H	2.99578800	6.86823000	-2.35350800	C	5.67463500	-6.94578700	4.30101200
H	-0.41369200	3.33208700	-2.97886000	C	4.47874000	-6.42159100	3.50307300
H	0.90307200	6.92099400	-1.02545800	S	4.48937700	-4.58264200	3.39514800
H	-1.51539600	4.35375300	-1.25132500	C	2.73442600	-4.29166400	2.98382300
C	8.41941800	2.58416400	-2.93814900	H	7.21969300	-5.60093100	3.54706400
C	7.62814200	1.34497100	-3.36552700	H	5.64766400	-6.51334700	5.30805100
C	7.14461100	0.54610400	-2.14877700	H	5.54081300	-8.02868000	4.42095100
C	6.45554800	1.74136100	-4.26687900	H	3.53971700	-6.71439100	3.98104900
H	7.78342200	3.26620300	-2.36046000	H	4.47292900	-6.83641100	2.48896900
H	8.30047200	0.69803000	-3.94901700	H	2.44764600	-4.80588800	2.06641800
H	6.61279200	-0.35926000	-2.45280900	H	2.08503700	-4.62263200	3.79777600
H	7.98210400	0.25408400	-1.50474300	H	2.62382800	-3.21729700	2.83031700
H	6.44478200	1.13767600	-1.54770300	C	8.25712100	2.01908100	2.13964700
H	5.74565400	2.36775000	-3.71502800	C	7.96042300	0.56856000	2.53674600
H	6.80076300	2.30857200	-5.13819200	C	6.64701400	0.08244300	1.91480500
H	5.91047400	0.86694200	-4.62550400	C	9.12292300	-0.35101900	2.14891000
C	4.31767500	-6.65110500	-1.89629600	H	8.37644000	2.10391100	1.05246000
C	2.81048300	-6.38309800	-1.87095000	H	7.85119700	0.53464700	3.63148300
C	2.37242500	-5.87148900	-0.49401000	H	6.42328800	-0.94706500	2.20862400
C	2.40289200	-5.40985200	-2.98420700	H	5.79957000	0.70541200	2.21803000
H	4.88251800	-5.73737700	-1.67508700	H	6.69400200	0.10379200	0.82083300
H	2.29721800	-7.33705400	-2.05685500	H	8.93477300	-1.38243400	2.46288000
H	2.85656300	-4.91536700	-0.26807200	H	9.26120700	-0.35723200	1.06078500
H	1.29407000	-5.70635900	-0.45988500	H	10.06452000	-0.02385300	2.60344800
H	2.64270300	-6.57984000	0.29735700	C	7.42015400	5.68341400	1.43079700
H	2.88785200	-4.43499100	-2.85320100	C	6.11473700	6.08837600	0.80071100
H	2.69592500	-5.79144000	-3.96881900	O	6.04558000	6.80518700	-0.20189100
H	1.32256500	-5.24164500	-2.99086200	H	7.30816100	5.34782400	2.46451500
C	8.69676800	-5.78469000	0.36826300	N	5.01009300	5.56817900	1.38749500
C	7.79069200	-4.77733400	-0.35783300	C	3.71974500	5.64001800	0.73765400
C	8.24127100	-4.61003600	-1.82171700	C	2.95771800	6.94246000	0.94332600

O	1.85912300	6.94845400	1.50269500	H	-2.50911500	0.86938200	6.79717600
H	3.08582400	4.84986200	1.13532100	H	-2.85015900	2.58826800	7.04746900
H	3.85016000	5.47446300	-0.33668800	H	-4.04516200	1.82333100	5.26346200
H	5.09889300	5.00292000	2.21812600	C	-0.41356700	-7.22762400	6.90830700
N	3.52061300	8.06104900	0.45053100	C	0.16167700	-6.82165800	5.54556700
C	2.83293600	9.33422100	0.58905200	C	0.01389400	-7.93335800	4.50431200
H	2.51292500	9.47962600	1.62370900	S	0.68544800	-7.54402900	2.84146500
H	1.94397500	9.37690500	-0.05044900	C	-0.52735900	-6.30027200	2.28538200
H	4.43186200	7.97605300	0.00584900	H	-0.29715200	-6.42341300	7.64049700
C	4.82746600	3.03685200	5.97048900	H	1.22261500	-6.56429200	5.64443800
C	4.76443500	2.60014100	4.49301300	H	-0.34501000	-5.91614500	5.19161300
C	3.52005000	1.80917200	4.14451000	H	-1.03709700	-8.22483900	4.39342600
C	2.32936500	2.45908400	3.78750500	H	0.56083600	-8.82647000	4.82438200
C	3.52323200	0.41002900	4.20418800	H	-0.25376700	-6.00333600	1.27202000
C	1.17799000	1.72851500	3.48936500	H	-1.53678000	-6.72119700	2.27700700
C	2.36937700	-0.32285800	3.91557000	H	-0.50448000	-5.40915100	2.91502800
C	1.19317400	0.33263500	3.54787400	C	-5.79759100	-1.40152400	4.67172900
H	3.99233100	3.70455300	6.19750600	C	-6.24832800	-0.55693100	3.50374800
H	5.65216300	2.00611800	4.24524000	O	-6.53005400	-1.07989900	2.41119500
H	4.80215700	3.50048700	3.86484000	H	-5.31576600	-0.80503500	5.44997800
H	2.30214300	3.54381900	3.77732300	N	-6.35981400	0.76609000	3.74279800
H	4.43663600	-0.11197800	4.47772900	C	-6.65792500	1.73409300	2.70846700
H	0.26459000	2.23779900	3.19756900	C	-5.42295400	2.53696300	2.31064500
H	2.39604500	-1.40634900	3.98072000	O	-4.37827600	2.50506900	2.96266900
H	0.29352800	-0.22503600	3.30720800	H	-7.04633200	1.20755800	1.83921500
C	0.85661000	5.72302500	7.20859100	H	-5.99804600	1.11268900	4.62323800
C	0.99291100	5.50338000	5.70530600	N	-5.62420900	3.26067100	1.19199700
O	2.08095400	5.22189000	5.20333300	C	-4.61007900	4.12933700	0.63664400
H	-0.14871500	6.02218900	7.51561100	C	-4.68938200	4.18733100	-0.88752000
N	-0.16435900	5.60191700	4.98912000	C	-4.50473000	2.79867700	-1.52651300
C	-0.17207600	5.68951300	3.53904900	C	-3.25970800	2.19417100	-0.92909500
C	-0.82691000	7.03818400	3.14316700	O	-2.14266300	2.64977100	-1.18561200
O	-1.34279200	7.75659500	3.99856800	O	-3.45921900	1.22494900	-0.06016100
H	-0.72598800	4.85695400	3.08738000	H	-3.63923700	3.76245200	0.97706300
H	0.85444800	5.66865100	3.16801400	H	-3.90826100	4.86517400	-1.24531800
H	-0.91493200	6.14043100	5.40881000	H	-5.65529200	4.60038300	-1.19184800
N	-0.79519600	7.33672500	1.81817200	H	-4.37005000	2.89052400	-2.60671900
C	-0.93792800	8.71672400	1.35480300	H	-5.36953400	2.16461600	-1.33379500
H	-0.00975900	9.28084600	1.50450200	H	-6.55342800	3.31111300	0.77823200
H	-0.13845600	6.78454600	1.28184600	C	-8.99185100	5.41937200	-1.94314700
C	-3.80009400	1.28267500	8.48241100	C	-8.64520300	4.06737100	-1.35537300
C	-3.29916100	1.58497400	7.06948300	O	-8.05848700	3.98482300	-0.26847100
O	-4.37733800	1.51397700	6.12385800	H	-8.06641600	5.97245400	-2.12612100
H	-4.23303100	0.27878600	8.53417400	H	-9.56118400	5.35471600	-2.87333800

N	-9.01298500	2.97505700	-2.06697000	H	-10.17163400	-3.67976000	-0.25194300
C	-8.66371000	1.64791200	-1.62218000	H	-7.83567300	-5.97148700	-2.14428400
C	-8.31116800	0.75752200	-2.82212500	H	-8.23650900	-2.86381100	0.79350300
O	-8.79380900	0.97856200	-3.93351600	H	-5.42101300	-6.12857500	-1.64754200
C	-9.79084500	0.99433400	-0.76643800	H	-5.84380400	-3.00991600	1.29719500
C	-11.07202300	0.74372200	-1.54349000	H	-4.40609700	-4.66402200	0.09344400
O	-9.36254300	-0.26552000	-0.24430300	H	-9.20878200	-1.83152800	-1.85627000
H	-7.79314200	1.73902400	-0.96744200	C	-0.84168100	-3.52133500	-1.67544700
H	-9.98629600	1.69519600	0.05833700	O	-0.77856700	-4.82470800	-1.22814800
H	-9.36608400	3.04035200	-3.01424600	C	0.33387700	-2.69464000	-1.16149200
H	-11.82133500	0.29755400	-0.88492400	C	0.18186700	-1.23446300	-1.68763900
H	-11.46895700	1.68306600	-1.93669100	C	-0.13499100	-1.28379300	-3.16661100
H	-10.89202800	0.06645200	-2.38235200	C	-0.56849100	-2.41422100	-3.76406900
H	-8.50869900	-0.14664400	0.19816100	O	-0.81870100	-3.56918800	-3.12995400
N	-7.50161000	-0.28202300	-2.50982500	C	0.52645300	-2.71880700	0.33095300
C	-7.17869700	-1.37055200	-3.41782100	C	-0.37601800	-3.04112500	1.25686000
C	-8.25245800	-2.46928500	-3.54867300	C	-0.88128100	-0.42341500	-0.92827700
O	-8.12782600	-3.30378800	-4.44096200	C	-0.46746700	0.16340600	0.41134600
C	-5.87474200	-2.08745900	-2.97494500	C	0.01808800	-0.05419400	-3.94235000
C	-4.55190400	-1.57582100	-3.57648100	O	0.35837100	1.02206000	-3.47501200
C	-4.48290500	-1.77477400	-5.09453000	O	-0.27893800	-0.18970400	-5.26474700
C	-4.24373300	-0.12843300	-3.19191500	C	-0.17158000	1.01645500	-6.03059700
H	-7.07410300	-0.96103300	-4.42600100	O	-1.33590700	0.66948400	1.15712800
H	-5.97539600	-3.13964100	-3.24666100	C	-1.88809400	-5.63322200	-1.62206200
H	-5.81852500	-2.05408800	-1.88149700	H	1.23419400	-3.10989300	-1.62665800
H	-3.76744100	-2.20722600	-3.13106300	H	1.14894900	-0.73765300	-1.56832300
H	-3.48689900	-1.51530500	-5.47121700	H	-1.81177000	-3.06781500	-1.41451400
H	-4.69999700	-2.81102200	-5.37075200	H	-0.75237500	-2.48451700	-4.82939000
H	-5.20664800	-1.13818900	-5.61480000	H	1.51041200	-2.37880400	0.64516600
H	-7.07098700	-0.29958000	-1.58493100	H	-0.13524800	-2.96732400	2.31273000
H	-3.25612000	0.17502500	-3.55809400	H	-1.37241400	-3.39588600	1.01442700
H	-4.98546200	0.55774200	-3.61420000	H	-1.76283800	-1.04426600	-0.72964100
H	-4.25488800	-0.01209100	-2.10720900	H	-1.23046300	0.39641900	-1.55634800
N	-9.23557000	-2.51184100	-2.61082400	H	0.48952800	-0.13259900	0.84234300
C	-9.98060300	-3.75197500	-2.40501500	H	-0.88818100	1.76353700	-5.68144400
C	-9.69533100	-4.32089200	-1.00230300	H	0.83324400	1.43826600	-5.94323000
C	-8.21599200	-4.42051600	-0.69743800	H	-0.39179100	0.73132200	-7.05936800
C	-7.39963400	-5.32875500	-1.38474500	H	-1.83947500	-5.87723900	-2.68675500
C	-7.62526800	-3.58437600	0.25707500	C	1.77169900	2.60247700	0.06434600
C	-6.03547700	-5.41252900	-1.10882300	N	0.62449900	1.89107300	-0.50765100
C	-6.26083200	-3.66555300	0.54132900	C	2.94260000	1.66069800	0.36681100
C	-5.46436000	-4.58531600	-0.13927200	N	4.35537600	-2.66656100	-1.10601200
H	-9.65434300	-4.43450100	-3.19124400	C	3.31686400	0.75364200	-0.78264400
H	-10.17186600	-5.30597500	-0.92385600	C	3.26550800	1.17234000	-2.11065800

C	3.54155300	0.29984000	-3.18032300
C	3.91165600	-1.02036000	-2.96381300
C	4.00083700	-1.43883700	-1.63337300
C	4.31192500	-2.60815800	0.27613800
C	3.89864600	-1.35754400	0.66265900
C	3.70193900	-0.58232100	-0.53599200
H	2.09019200	3.41203700	-0.60611600
H	0.79418200	1.64789000	-1.48216400
H	-0.20902200	2.47165900	-0.49817100
H	2.68709800	1.05117700	1.23595600
H	3.80654100	2.26835300	0.67120200
H	4.70592500	-3.44372200	-1.64034300
H	2.97270800	2.19174800	-2.33792700
H	3.46162600	0.66647700	-4.19732900
H	4.13231800	-1.68784100	-3.78585400
H	4.59655500	-3.45961600	0.87742400
H	3.78097400	-1.01671800	1.67925100
H	1.45016200	3.06924800	0.99939500
H	8.56326900	1.56116300	-8.33450500
H	7.18375700	2.38970300	-7.60320000
H	9.27719400	2.31689300	-2.30966900
H	8.79696500	3.13804900	-3.80439000
H	3.22459600	6.58889700	-5.66148800
H	4.48811400	5.49210000	-6.25252100
H	4.65033000	-7.01494000	-2.87405900
H	4.60492500	-7.39329800	-1.14358300
H	7.84868000	-7.09600300	4.25303800
H	7.08681500	-7.12072100	2.65429300
H	8.65201900	-6.77344700	-0.10137900
H	8.40960600	-5.89061900	1.41665200
H	0.09269700	-8.11557900	7.30232100
H	-1.48138000	-7.46114600	6.83302500
H	-4.56531400	2.00389500	8.78363000
H	-2.96680900	1.33837700	9.18897200
H	5.76428100	3.56414100	6.17797500
H	4.77144700	2.16573300	6.63109300
H	-9.56827800	5.98192500	-1.20496700
H	-11.05682300	-3.58361500	-2.52694000
H	3.51256400	10.13800100	0.30355400
H	-5.10619500	-2.16066300	4.30122500
H	-6.66402400	-1.92057300	5.09361400
H	-4.71050400	5.14660300	1.03889400
H	-7.43335200	2.42553700	3.05911300
H	-1.83094000	-6.54665700	-1.02878900
H	-2.83610600	-5.11955000	-1.41760700
H	9.17632400	2.38673600	2.60830400
H	7.43745900	2.68419500	2.43568600
H	7.85129800	4.86099800	0.85035700
H	8.11797500	6.52137500	1.39285500
H	1.12555300	4.79568900	7.72172100
H	1.57291500	6.48951900	7.51379000
H	-1.19340900	8.71191100	0.29295100
H	-1.73713400	9.19832100	1.91810500
H	-2.56998000	0.98736700	0.46655500
O	-6.18464800	0.01947800	0.00664500
H	-6.25457300	-0.47872100	0.85448300
H	-5.29798400	0.42047400	0.01472300

Int2 (2.5)

C	-7.38246900	-1.80248600	-8.27006400
C	-6.86830600	-0.80457500	-7.22143500
C	-5.54119500	-1.15354400	-6.60409300
C	-4.75449700	-2.24769600	-6.86724100
C	-4.84370100	-0.39288400	-5.58890100
C	-3.63899800	-1.09198500	-5.28301200
C	-5.13008500	0.78897600	-4.88541300
N	-3.60667100	-2.21004300	-6.08993000
C	-2.74892000	-0.65464900	-4.29692200
C	-4.23811800	1.23447800	-3.91355600
C	-3.06234900	0.51807000	-3.62211700
H	-6.68451000	-1.88697800	-9.10971900
H	-7.61689400	-0.70843700	-6.42238300
H	-6.80125100	0.19331700	-7.67668800
H	-4.91381400	-3.06284700	-7.55694900
H	-6.04180500	1.34284100	-5.08967500
H	-2.90340200	-2.92969700	-6.06841400
H	-1.86182700	-1.21101600	-4.02626900
H	-4.45440300	2.13906600	-3.35798200
H	-2.38908200	0.87003900	-2.84782300
C	-3.47991300	-5.74628500	-5.58976700
C	-3.50568700	-5.17406900	-4.16502300
C	-2.18621200	-5.17718500	-3.42238500
C	-1.25816200	-4.14030600	-3.58055100
C	-1.87674500	-6.16939400	-2.48144000
C	-0.10047600	-4.05007600	-2.81053000
C	-0.71726800	-6.11032800	-1.71071200
C	0.16430800	-5.02996700	-1.84281400
O	1.22124600	-4.95827300	-0.99238600

H	-2.74813700	-5.22338000	-6.21854400	H	-6.77725200	2.64150100	-2.89579900
H	-4.24795700	-5.72137400	-3.57311600	H	-5.67712500	3.53794200	-1.83927700
H	-3.87172000	-4.14736300	-4.21901300	H	-6.63379100	2.18883400	-1.20051900
H	-1.46815000	-3.34188400	-4.28494200	C	-7.08983500	6.75322500	3.31128800
H	-2.57566300	-6.98810200	-2.32427800	C	-5.60124000	7.09916900	3.21935900
H	0.54659000	-3.18299600	-2.88645600	C	-4.88465000	6.42586000	2.04657200
H	-0.51701400	-6.86069900	-0.95645500	S	-4.65802800	4.60550000	2.23426600
H	1.67939400	-4.08281000	-1.09842400	C	-2.98981300	4.58949200	2.98080400
C	-8.37688300	-2.71299100	-3.00224700	H	-7.23228300	5.67281600	3.41075500
C	-7.07661000	-1.92277900	-3.14445000	H	-5.09110300	6.83308000	4.15293200
C	-6.86954400	-0.96013300	-1.97310400	H	-5.48246800	8.18489700	3.10362600
C	-5.89337800	-2.87922500	-3.28161900	H	-3.89069900	6.85365400	1.90654200
H	-8.34625200	-3.34868800	-2.10882900	H	-5.44225600	6.57306500	1.11657000
H	-7.13316300	-1.32357200	-4.06229500	H	-2.24455300	4.95752100	2.27282800
H	-5.97420400	-0.35179200	-2.13562700	H	-2.95653200	5.20514000	3.88138500
H	-7.71939600	-0.27834800	-1.85414600	H	-2.76568300	3.55500300	3.24577000
H	-6.74444000	-1.50810900	-1.03351300	C	-8.27288200	-1.98870200	2.05730100
H	-5.81552500	-3.54083800	-2.41038400	C	-7.51208400	-0.78578200	2.63806400
H	-6.00628600	-3.50766000	-4.16921100	C	-6.32719700	-0.39578300	1.74734800
H	-4.95602200	-2.32878600	-3.38634400	C	-8.45011700	0.40662400	2.85563700
C	-4.30947500	6.56190500	-2.20407800	H	-8.69965000	-1.74216700	1.07846100
C	-2.80760700	6.27615900	-2.11977700	H	-7.11643000	-1.08816200	3.61916300
C	-2.36711400	6.12769200	-0.65977600	H	-5.76645800	0.44527700	2.16350400
C	-2.43265500	5.03258900	-2.93545500	H	-5.62178100	-1.22350700	1.61787500
H	-4.87310300	5.70382100	-1.82270100	H	-6.67602200	-0.10628000	0.75130000
H	-2.27193200	7.13387600	-2.55140700	H	-7.91632100	1.25928800	3.28826200
H	-2.89401200	5.29233500	-0.18149100	H	-8.88028900	0.73751700	1.90343800
H	-1.29286200	5.94630700	-0.58406400	H	-9.27758800	0.14892400	3.52539200
H	-2.60300600	7.03124300	-0.08722600	C	-7.41923700	-5.67127800	1.47221400
H	-2.89314900	4.13301100	-2.50568300	C	-6.14675400	-5.76374600	0.67443000
H	-2.77733700	5.11161100	-3.97194200	O	-6.12514600	-5.89121500	-0.54634700
H	-1.34912700	4.88452700	-2.95230300	H	-7.26369500	-5.70548200	2.55224100
C	-8.71207200	5.75472700	0.03719600	N	-5.01289600	-5.71281700	1.42866200
C	-7.70903500	4.60579300	-0.12018400	C	-3.71121800	-5.63923300	0.81989000
C	-7.76964700	4.03102700	-1.54607600	C	-2.88133400	-6.90248700	0.95644300
C	-7.94499000	3.52505600	0.94104100	O	-1.67962100	-6.85522900	1.21897500
C	-6.65309200	3.04211500	-1.88570100	H	-3.12947500	-4.81712100	1.23985800
H	-9.73273700	5.40643400	-0.16857100	H	-3.85580600	-5.43741700	-0.24498600
H	-6.69809700	5.01048100	0.03007900	H	-5.08749200	-5.55814700	2.42230500
H	-7.73455600	4.86047100	-2.26436000	N	-3.52529900	-8.06917300	0.73679800
H	-8.74585800	3.54644500	-1.68802100	C	-2.81446900	-9.33383700	0.79663000
H	-8.93959100	3.07859400	0.82047900	H	-2.26355200	-9.41164500	1.73694000
H	-7.20654400	2.72149100	0.88258600	H	-2.09832200	-9.42949600	-0.02640100
H	-7.88713200	3.94226400	1.95054200	H	-4.49571800	-8.04233600	0.46240500

C	-4.88354200	-2.87705200	5.95578400	H	-1.31998500	6.58689300	5.48797400
C	-4.31363900	-2.25459500	4.66820900	H	0.23827900	6.49473200	4.68097000
C	-3.64653300	-0.91989000	4.91654000	H	0.17284100	9.04578800	4.42270600
C	-2.31065400	-0.85921400	5.33625900	H	-1.44998400	9.02150600	5.10574600
C	-4.35242900	0.27961700	4.76414800	H	-0.32894000	7.47392500	0.92465600
C	-1.69405400	0.37049400	5.57156900	H	0.79995600	8.28179300	2.03917500
C	-3.74331300	1.51131900	5.00436000	H	0.29014300	6.58883700	2.31744700
C	-2.40615700	1.56127600	5.40381600	C	5.74294900	1.54822100	4.63722300
H	-4.07238600	-3.10699500	6.65147000	C	6.23157900	0.67042700	3.50782800
H	-5.11741700	-2.12064800	3.93486100	O	6.54949500	1.16984400	2.41733700
H	-3.58342500	-2.94711500	4.23838700	H	5.48348800	0.98161000	5.53390000
H	-1.76533800	-1.78805700	5.47464700	N	6.31719200	-0.64776900	3.78391700
H	-5.39046700	0.24944900	4.44889200	C	6.63459600	-1.64422700	2.78243500
H	-0.65470200	0.39893800	5.88866800	C	5.40312400	-2.44327800	2.36535000
H	-4.31206500	2.42743500	4.87363000	O	4.32823600	-2.36057100	2.97409700
H	-1.92721100	2.51820700	5.59171700	H	7.05084100	-1.14388900	1.90981600
C	-0.92079600	-5.51166800	7.32176500	H	5.89981000	-0.96395500	4.65240800
C	-0.85786900	-4.99184000	5.87573600	N	5.63380600	-3.23589400	1.30630300
O	-1.64337800	-4.14594700	5.44102500	C	4.61549200	-4.10895000	0.76433100
H	0.02798200	-5.93742400	7.65623500	C	4.72539100	-4.30598900	-0.74619800
N	0.11418000	-5.56198000	5.12146100	C	4.62160200	-2.98719400	-1.53231100
C	0.14858600	-5.59041800	3.66463700	C	3.50286400	-2.15177900	-0.93195400
C	0.65711900	-7.01351900	3.31856900	O	2.30873200	-2.53671100	-1.06201900
O	0.84686500	-7.83153000	4.22296000	O	3.82939500	-1.13573300	-0.22748200
H	0.82003900	-4.83083700	3.24676400	H	3.64559800	-3.68445100	1.02831900
H	-0.84787300	-5.43850600	3.23885300	H	3.91203200	-4.97541500	-1.04269200
H	0.56633000	-6.39304700	5.49668400	H	5.67042100	-4.80347200	-0.98631800
N	0.88340300	-7.28939500	2.01777700	H	4.40475900	-3.19512200	-2.58365100
C	0.94740200	-8.68194700	1.58481900	H	5.55887500	-2.43120800	-1.47843900
H	-0.03863100	-9.02014000	1.25700600	H	6.56487400	-3.28826200	0.89605900
H	0.50683900	-6.63436100	1.34418100	C	9.02897500	-5.46705800	-1.72478800
C	3.71059400	-1.02058200	8.50773800	C	8.68289900	-4.09820600	-1.17418900
C	3.33115000	-1.03436100	7.02455300	O	8.10628200	-3.98375300	-0.08474600
O	4.42302700	-1.52491700	6.23209600	H	8.10155700	-6.00882100	-1.93193900
H	4.58692700	-0.38691000	8.67627000	H	9.63040000	-5.42693600	-2.63592100
H	3.05274400	-0.01844300	6.70322700	N	9.04212600	-3.02978100	-1.92401900
H	2.44881600	-1.67311900	6.88003300	C	8.68799900	-1.68839100	-1.52576900
H	4.07373300	-1.82826800	5.37786500	C	8.34619800	-0.84401800	-2.76277600
C	0.32055600	7.42684100	6.63032500	O	8.81045600	-1.12770900	-3.86896100
C	-0.39217700	7.13997700	5.30403100	C	9.80849500	-1.00956600	-0.68310800
C	-0.72309100	8.42558500	4.54446300	C	11.09705500	-0.78916400	-1.45794200
S	-1.47839000	8.18416500	2.89096000	O	9.37818900	0.26440500	-0.20257200
C	-0.02848900	7.57146700	1.96756400	H	7.81182600	-1.75445800	-0.87740100
H	0.54066000	6.50069900	7.17024900	H	9.99644300	-1.68660600	0.16356700

H	9.37921300	-3.12525900	-2.87485600
H	11.83919400	-0.31350700	-0.81168200
H	11.49995100	-1.74323700	-1.80762600
H	10.92489700	-0.14776400	-2.32647200
H	8.48457800	0.16701500	0.16418000
N	7.56564300	0.22457400	-2.48488600
C	7.21594000	1.26817300	-3.43148100
C	8.23322200	2.41801300	-3.54460000
O	8.05219900	3.28123100	-4.40043800
C	5.84061700	1.88719000	-3.07913800
C	4.62799500	1.26509600	-3.79275200
C	4.60858600	1.59215500	-5.28937100
C	4.52279500	-0.23942400	-3.54335000
H	7.19273100	0.82376500	-4.43103800
H	5.87830700	2.94900500	-3.32741700
H	5.70392700	1.82270800	-1.99420500
H	3.74083000	1.73367700	-3.34379600
H	3.68328200	1.22886100	-5.74948000
H	4.67830900	2.67087800	-5.46181500
H	5.44871500	1.12346600	-5.81344200
H	7.14006600	0.27675400	-1.55677900
H	3.59621100	-0.64472300	-3.96043400
H	5.35996000	-0.78007100	-3.99802400
H	4.53111700	-0.44056300	-2.47286500
N	9.26412700	2.44368300	-2.65740400
C	10.00045300	3.68812100	-2.46255600
C	9.64920000	4.31466200	-1.09677000
C	8.15744000	4.36469700	-0.84064000
C	7.31700800	5.17078400	-1.62065900
C	7.58042300	3.57156500	0.15824200
C	5.94057400	5.18978900	-1.39987400
C	6.20418200	3.59354700	0.39190500
C	5.38296500	4.40448600	-0.38894400
H	9.71268100	4.34066200	-3.28777900
H	10.08482100	5.32088400	-1.05237000
H	10.12462200	3.72913300	-0.30195700
H	7.74072700	5.76920300	-2.42152700
H	8.20915100	2.92549900	0.76450200
H	5.30461600	5.81217900	-2.02330800
H	5.79718300	2.97115400	1.18063300
H	4.31154500	4.42106700	-0.21070100
H	9.26001500	1.76097700	-1.90396500
C	1.24120400	4.22797200	-1.14386200
O	1.00321500	5.54119500	-0.79016300

C	0.17742700	3.30954100	-0.54507400
C	0.48770900	1.84725500	-0.97499100
C	0.77625900	1.81416800	-2.45449500
C	1.08767600	2.93102300	-3.13979200
O	1.20594300	4.15659300	-2.59246900
C	-0.00972800	3.41219300	0.94371600
C	0.78370400	4.01285500	1.83586800
C	1.64713000	1.21651800	-0.17431500
C	1.19387700	0.52814500	1.12910700
C	0.66323600	0.50821000	-3.12181200
O	0.21511600	-0.48856300	-2.57568000
O	1.10369000	0.52092400	-4.40114400
C	0.99061600	-0.71821400	-5.11755100
O	1.88729500	0.94758200	2.27959500
C	2.00430000	6.46530500	-1.22020500
H	-0.77200600	3.59137700	-1.01443300
H	-0.41122100	1.24540800	-0.80274000
H	2.25825400	3.91755000	-0.85684500
H	1.24995300	2.94124800	-4.21058300
H	-0.89484700	2.88721000	1.29718800
H	0.52846200	4.00686100	2.89258300
H	1.66932800	4.57003100	1.55030600
H	2.39710500	1.96758200	0.08958100
H	2.17515800	0.49411700	-0.79805500
H	0.11383600	0.62820600	1.26077100
H	1.40504000	-1.54012700	-4.53122400
H	-0.05673400	-0.92505100	-5.34792300
H	1.55866500	-0.57751900	-6.03631200
H	2.06381900	6.50346900	-2.31108700
C	1.12149300	-1.80539300	2.16158600
N	1.48849100	-0.92332400	1.01036300
C	-0.34049900	-1.64180200	2.59365400
N	-3.62019500	0.52271100	-0.17485500
C	-1.31400400	-1.71187800	1.43455700
C	-1.36388600	-2.83212500	0.60578200
C	-2.20533900	-2.88668300	-0.52241800
C	-3.00462800	-1.81309300	-0.88257900
C	-2.95819200	-0.68378100	-0.06301000
C	-3.29134600	1.33181700	0.89549800
C	-2.39833500	0.67186700	1.70670800
C	-2.15231800	-0.61904900	1.11146900
H	1.32317800	-2.81784500	1.80533500
H	1.05117300	-1.29200500	0.15241000
H	2.51899900	-1.00949500	0.76154800

H	-0.45806000	-0.69754000	3.12877100
H	-0.55423800	-2.42849200	3.32703800
H	-4.26571700	0.75624300	-0.91288700
H	-0.72997900	-3.69006000	0.81286400
H	-2.21163500	-3.77914900	-1.13100700
H	-3.62748600	-1.84500000	-1.76671200
H	-3.74151000	2.30915100	0.99991400
H	-2.02206000	1.03416200	2.65170500
H	1.81806300	-1.58612900	2.96908800
H	-8.34956800	-1.48210300	-8.66661200
H	-7.50943700	-2.79908000	-7.83491000
H	-9.24689100	-2.05230800	-2.91643500
H	-8.53623300	-3.36876000	-3.86555800
H	-3.19635300	-6.80442600	-5.59122200
H	-4.46287200	-5.65572500	-6.06369500
H	-4.63023600	6.73979100	-3.23619700
H	-4.58782900	7.43760000	-1.60697500
H	-7.55698600	7.23463700	4.17620500
H	-7.61970100	7.08374400	2.41582700
H	-8.49468500	6.57184000	-0.65888400
H	-8.70257000	6.16030800	1.04987500
H	-0.29610800	8.05470900	7.28283100
H	1.26815200	7.95127500	6.46530700
H	3.94586600	-2.03100900	8.85322800
H	2.87850900	-0.62801300	9.09997100
H	-5.41284600	-3.81132900	5.73708200
H	-5.58478600	-2.19489800	6.44795800
H	9.56998900	-6.02431700	-0.95673200
H	11.08005400	3.51168200	-2.52433900
H	-3.53562100	-10.15043600	0.73397700
H	4.86215800	2.09327600	4.28688100
H	6.51488300	2.28509000	4.87348300
H	4.66241200	-5.09219600	1.25359400
H	7.39239500	-2.33453000	3.17125500
H	1.70554900	7.43972600	-0.83267500
H	2.98645800	6.19803500	-0.81055600
H	-9.09099500	-2.30107000	2.71492200
H	-7.60036700	-2.83806000	1.92175000
H	-7.93065000	-4.73997600	1.22583800
H	-8.08174800	-6.49164500	1.17966600
H	-1.21224100	-4.69257900	7.98107000
H	-1.69154000	-6.28681500	7.37907100
H	1.65304500	-8.78157400	0.75572400
H	1.28595300	-9.28605500	2.42513200
H	1.65155000	1.88077300	2.39813700
O	6.33834500	-0.05855400	0.01943100
H	6.30036700	0.49111500	0.83034200
H	5.45220000	-0.48644900	-0.07460900

Int3 (11.5)

C	17.59399000	43.88500000	-6.44712600
C	18.90908200	44.59277200	-6.08030600
C	20.05319200	43.64772600	-5.82962500
C	19.98339300	42.29831100	-5.58379600
C	21.45921700	43.98630700	-5.81544600
C	22.18625600	42.78632600	-5.56756600
C	22.17684800	45.17779200	-6.01644100
N	21.25723500	41.77817700	-5.40996700
C	23.58295100	42.74918400	-5.54491200
C	23.56764800	45.14457400	-5.99673800
C	24.26285900	43.93996700	-5.76902000
H	17.72652700	43.31841400	-7.37609200
H	18.74785500	45.21936600	-5.19167600
H	19.19189500	45.28655000	-6.88269200
H	19.12432600	41.64825600	-5.51545600
H	21.65079100	46.11021000	-6.19895400
H	21.46821200	40.80234700	-5.28638100
H	24.12673400	41.83180500	-5.35447900
H	24.13097700	46.05726800	-6.16260700
H	25.34720100	43.94044900	-5.74643200
C	19.01581200	39.17826500	-2.73328000
C	19.81916000	40.19454100	-1.90145300
C	21.16963200	39.65361200	-1.47527100
C	22.27905800	39.73495100	-2.32415600
C	21.36806200	39.03934200	-0.23014400
C	23.53118700	39.23938700	-1.96154600
C	22.61243800	38.54159800	0.15813000
C	23.70671900	38.64607300	-0.70734000
O	24.91789100	38.18198900	-0.26693700
H	19.56899800	38.96593700	-3.65919600
H	19.24941500	40.49232500	-1.01404000
H	19.97070000	41.09905700	-2.49125100
H	22.17035300	40.23383400	-3.28395400
H	20.53159000	38.96067300	0.46101700
H	24.38714500	39.37452400	-2.60940200
H	22.75160600	38.11386300	1.14339000
H	25.61198000	38.29498600	-0.94118800
C	18.62694000	45.31684200	-1.30406300

C	19.93007100	45.06186700	-2.07524700	H	29.95167000	51.58125100	-0.02195100
C	21.07864100	45.86208000	-1.45068600	H	29.62431700	51.86006000	-1.72479000
C	20.25842500	43.57359100	-2.13509900	H	30.12859700	49.54935200	-1.50606900
H	18.74964700	45.03671200	-0.25051900	H	28.38276900	49.64056100	-1.80367000
H	19.79355600	45.41715900	-3.10714100	H	29.04836400	46.90186400	-0.94384000
H	22.00858900	45.72999400	-2.00992500	H	30.66526900	47.46692900	-0.44438400
H	20.84847300	46.93361200	-1.41089900	H	29.60446800	46.62130800	0.71019500
H	21.26572300	45.52138700	-0.42647400	C	21.29404100	46.95199600	2.73893200
H	20.37641200	43.15691000	-1.12802400	C	22.80332800	47.18715300	2.60661100
H	19.44787000	43.03553800	-2.63328600	C	23.44783500	46.12231500	1.71072300
H	21.17958700	43.38455900	-2.69325800	C	23.09840200	48.59251800	2.07051100
C	27.28117100	48.52787500	-5.54692500	H	20.82288800	46.98106900	1.74788000
C	28.17097000	47.29027200	-5.38390500	H	23.24552300	47.11065500	3.61086800
C	28.75589200	47.23165000	-3.96681600	H	24.53570500	46.22266500	1.68027800
C	27.40598600	46.00191600	-5.70678600	H	23.21398900	45.10759400	2.05028500
H	26.36385400	48.40803600	-4.95566500	H	23.09050600	46.21267000	0.68004600
H	29.00854500	47.37799000	-6.09083400	H	24.17588900	48.75879100	1.96748600
H	27.95782800	47.10424300	-3.22466100	H	22.64828500	48.72665700	1.08033600
H	29.45370700	46.39805200	-3.85959000	H	22.69561900	49.36908100	2.72987500
H	29.29285800	48.15371900	-3.71844000	C	19.41399300	43.82106300	3.87600000
H	26.60399700	45.84225900	-4.97503300	C	19.92575800	42.70656700	3.00752900
H	26.94788700	46.04173500	-6.70144100	O	19.46120800	42.44930000	1.90283000
H	28.06915300	45.13347100	-5.67146900	H	19.76724400	43.80046400	4.90833700
C	24.77607600	51.75296700	-2.65202700	N	20.95946100	42.00396300	3.56142500
C	24.51465600	50.23663900	-2.69141800	C	21.66305000	40.99188400	2.82102600
C	23.55909500	49.87474400	-3.84212400	C	21.51890500	39.58502900	3.39252000
C	23.99955000	49.75230300	-1.32969400	O	22.44242300	38.77167400	3.33644400
C	23.34109700	48.37494700	-4.06104900	H	22.73419700	41.20506500	2.76914200
H	23.83616800	52.30421200	-2.53082000	H	21.27061700	40.99808000	1.79976400
H	25.47800400	49.73804300	-2.88746000	H	21.34472200	42.30594600	4.44374100
H	23.94516100	50.31646300	-4.76982100	N	20.30995900	39.28451500	3.91955300
H	22.58987600	50.35743700	-3.65473500	C	20.04492600	37.96005300	4.44700000
H	23.03555100	50.21936900	-1.09440600	H	20.81147800	37.69364200	5.18019800
H	23.86039800	48.66884500	-1.29007700	H	20.06434900	37.20908300	3.64938200
H	24.69905300	50.01587900	-0.53006200	H	19.59323100	39.99353700	3.91919000
H	22.66953200	48.18928000	-4.90436600	C	24.72113600	44.88201000	6.12206000
H	24.28464900	47.86783400	-4.29873400	C	25.02109100	44.65057600	4.62944200
H	22.89658700	47.88745400	-3.18792600	C	26.48657500	44.56491300	4.27060800
C	27.87197600	52.02405900	-0.48625700	C	27.19674300	43.36852000	4.45002600
C	29.22603400	51.38369500	-0.81935500	C	27.15825600	45.66250100	3.71986500
C	29.17642600	49.88079600	-1.08614900	C	28.53480600	43.27076000	4.06986100
S	28.91927800	48.90078600	0.45239700	C	28.50180600	45.57188000	3.34939200
C	29.63232000	47.32229700	-0.12548300	C	29.19347100	44.37074000	3.51410700
H	27.44588500	51.61317200	0.43510500	H	25.12644300	44.05319000	6.71070800

H	24.56244100	45.44910500	4.04218400	O	33.87739100	37.98848700	-0.14765100
H	24.53266000	43.71590300	4.32885200	H	33.82616000	39.81033100	2.58394300
H	26.67673800	42.51248800	4.86843300	N	32.65939100	37.69163700	1.76476700
H	26.62066600	46.59416300	3.56518300	C	31.89695200	36.54102900	1.30797600
H	29.06206600	42.32862200	4.19939500	C	30.41047500	36.75878300	1.56294200
H	29.00372700	46.44139700	2.93489700	O	29.98735200	37.81607100	2.05364400
H	30.23566300	44.29427700	3.21716300	H	32.02784600	36.45977200	0.22833800
C	26.40688400	40.65000800	8.06102000	H	32.39984100	38.11174800	2.65217600
C	25.82954700	40.52022100	6.65383100	N	29.62592400	35.75882200	1.12585600
O	25.09111900	41.37495000	6.17102200	C	28.19124000	35.92301200	0.98321900
H	27.12552200	39.86944300	8.31970700	C	27.66711100	35.42825600	-0.37825000
N	26.23044100	39.40756900	5.95807400	C	28.42628400	35.95505600	-1.60797500
C	25.52003000	38.92399400	4.78093200	C	28.49380400	37.45731200	-1.53200400
C	24.55807000	37.78656800	5.19450100	O	27.51317700	38.17556900	-1.63382200
O	23.98964300	37.79752400	6.27806600	O	29.71974200	37.92100600	-1.26338600
H	26.22747300	38.60586200	4.00846700	H	27.95627700	36.97565800	1.14881900
H	24.91758100	39.74729700	4.39021000	H	26.61648000	35.72903700	-0.44568000
H	26.67882700	38.68897600	6.50879800	H	27.70244900	34.33441200	-0.40028600
N	24.44418600	36.77054600	4.29278900	H	27.89646800	35.66332200	-2.51776100
C	23.31797300	35.84497800	4.34301400	H	29.43039500	35.53847100	-1.64298600
H	22.38415500	36.38040700	4.15320400	H	30.04404700	34.91937200	0.72431300
H	24.75459900	36.99300200	3.35564100	C	29.29900400	31.23695200	-1.09703900
C	32.73997700	40.47096600	6.36296700	C	30.12052900	32.50958000	-1.18738300
C	32.24932000	40.69470100	4.93484800	O	30.33573400	33.21442300	-0.19224900
O	31.91818100	39.42439000	4.34269800	H	28.23653200	31.49336500	-1.03105300
H	33.53697100	39.72083800	6.36327000	H	29.44926700	30.57156900	-1.94959200
H	33.02931800	41.18411300	4.33435700	N	30.59945600	32.80660600	-2.41467700
H	31.37412000	41.35659800	4.93186800	C	31.41903100	33.96301900	-2.68095700
H	31.09614600	39.49878000	3.83960100	C	31.03959600	34.52013600	-4.06096300
C	34.70897100	47.89975300	1.13608400	O	30.44046100	33.81317100	-4.87676100
C	33.63071700	47.84887800	0.04401300	C	32.93622000	33.58747900	-2.63474600
C	33.94353700	48.81392900	-1.09927000	C	33.33352100	32.56741500	-3.68991300
S	32.64613800	48.96882900	-2.38444200	O	33.75899000	34.73337700	-2.82503500
C	32.51034300	47.24663700	-2.96944300	H	31.24983400	34.70318000	-1.89978000
H	34.57332100	47.07780500	1.84718600	H	33.10345300	33.16495200	-1.63233300
H	32.65327100	48.09693500	0.47174100	H	30.26977000	32.32531200	-3.24372500
H	33.55244600	46.83012300	-0.35292300	H	34.40040900	32.34866000	-3.60044500
H	34.88735100	48.53783800	-1.58395300	H	32.77605100	31.63659700	-3.56000100
H	34.06429300	49.83286300	-0.71645300	H	33.14121400	32.95170900	-4.69551300
H	31.85556000	47.26707200	-3.84133700	H	33.40802800	35.44948000	-2.26791200
H	33.49018200	46.86169900	-3.26614300	N	31.46151100	35.78044500	-4.28467700
H	32.05767400	46.58559400	-2.22889100	C	31.38095600	36.46295500	-5.56002700
C	34.00199600	39.69600500	1.51297100	C	32.72205900	36.55455700	-6.30598300
C	33.52279500	38.36747800	0.98193600	O	32.76999900	37.18498700	-7.36023700

C	30.75871200	37.87577300	-5.39801900	C	26.79109500	40.89010400	-3.89915100
C	29.32713300	38.00330900	-5.95102000	O	26.24538800	40.43592200	-2.90687200
C	29.29584900	37.81204300	-7.47248300	O	26.46599200	40.48433900	-5.15468500
C	28.34544600	37.05680800	-5.24799500	C	25.52172900	39.40715300	-5.22680100
H	30.74725800	35.84286400	-6.19953700	O	29.82774900	40.01890000	0.13162600
H	31.39680700	38.60049200	-5.90666300	C	32.01997300	44.30531700	-4.94268800
H	30.76655800	38.13440300	-4.33270900	H	28.47503600	44.42368100	-3.01076200
H	29.00113000	39.03182000	-5.73935200	H	27.43835600	42.59064100	-1.90646600
H	28.29821400	38.02891600	-7.86956800	H	30.78916500	42.51957800	-3.59008000
H	30.02010800	38.46395200	-7.96852900	H	28.10040900	42.03300400	-5.97553500
H	29.54515600	36.78363300	-7.75444000	H	28.96884000	44.36550100	-0.62387500
H	31.90465100	36.28447400	-3.51768200	H	31.29327200	44.44595800	0.02601900
H	27.35649700	37.10521400	-5.71492700	H	31.82935400	43.89763900	-1.65511400
H	28.67804300	36.01422600	-5.29961900	H	30.17776100	41.33090500	-1.90121900
H	28.22707000	37.32832800	-4.19533300	H	28.82552800	40.25865800	-2.22141600
N	33.78995000	35.91242800	-5.75838800	H	29.12460200	41.95576700	0.29314100
C	35.13006800	36.23804100	-6.22475000	H	25.83474500	38.58215000	-4.58537800
C	35.85815800	37.15643100	-5.21900300	H	24.52705600	39.73921500	-4.91788800
C	35.02238400	38.34111400	-4.77513100	H	25.50711300	39.10024200	-6.27195600
C	34.51783200	39.26575300	-5.70125700	H	31.60752600	44.10860700	-5.93585100
C	34.70452700	38.51621800	-3.42399900	C	26.78255400	41.23601700	1.16113200
C	33.71359300	40.32594900	-5.28589100	N	27.51186300	40.67085900	0.03484400
C	33.90736200	39.58141600	-3.00090600	C	26.46305900	42.73871600	1.09638500
C	33.40530700	40.48654100	-3.93298900	N	25.38626100	46.33610000	-1.85456000
H	35.00184600	36.73609800	-7.18691800	C	25.67987400	43.20965600	-0.10607000
H	36.79671400	37.49432100	-5.67777000	C	24.79854200	42.39488200	-0.81110600
H	36.13382500	36.56988300	-4.33551400	C	24.08518000	42.86225100	-1.93229300
H	34.72129900	39.13386300	-6.75858200	C	24.21466300	44.16637300	-2.38622100
H	35.07935000	37.81301100	-2.68595500	C	25.07773400	45.00039900	-1.67056700
H	33.31888100	41.02052500	-6.02207400	C	26.27601800	46.74272800	-0.87666500
H	33.68246000	39.68120600	-1.94486400	C	26.57085900	45.67714800	-0.06165500
H	32.77618300	41.31028200	-3.60728200	C	25.81560800	44.54858700	-0.54141400
H	33.69746500	35.51593400	-4.82621000	H	25.86276000	40.65086600	1.25883000
C	30.17807300	43.41647700	-3.78046700	H	26.95280200	40.43442400	-0.77438400
O	30.98094200	44.52114400	-3.98606000	H	29.71386300	38.84903800	-0.80352700
C	29.16407800	43.64492900	-2.66214300	H	27.40065100	43.30354200	1.15753700
C	28.33010700	42.34178300	-2.48902300	H	25.92589200	43.00493500	2.01524600
C	27.86913900	41.87818300	-3.85266900	H	24.98227100	46.93125400	-2.55893800
C	28.44529900	42.31808800	-4.98892600	H	24.65244600	41.36313600	-0.51357200
O	29.49115300	43.16017700	-5.03600800	H	23.42430700	42.17937200	-2.45102400
C	29.73615900	44.08908700	-1.34416700	H	23.67547800	44.51685900	-3.25719000
C	31.01734000	44.13832800	-0.97800900	H	26.61572100	47.76621000	-0.81465400
C	29.09871500	41.21131700	-1.76519800	H	27.20654200	45.71022700	0.80941500
C	28.83294900	41.06026000	-0.26552300	H	27.34939400	41.05846400	2.08446500

H	16.79249900	44.60999200	-6.62309600
H	17.36385900	43.15234800	-5.66643600
H	18.42045600	46.39116500	-1.35430800
H	17.78085900	44.74150900	-1.69372000
H	19.01192700	38.23043000	-2.18425600
H	18.03104100	39.53857200	-3.04743200
H	26.97417800	48.70098500	-6.58380400
H	27.79495500	49.40637300	-5.14285100
H	28.01754500	53.10050500	-0.34823800
H	27.17598000	51.81299600	-1.30457300
H	25.25399600	52.09521500	-3.57581500
H	25.41478900	51.98486500	-1.79338800
H	34.62255500	48.82959500	1.70819500
H	35.70662000	47.78961300	0.69828600
H	31.92265900	40.09424900	6.98672800
H	33.13793400	41.38126600	6.82353100
H	23.63383000	44.86024900	6.25089500
H	25.16669400	45.81304700	6.48742500
H	29.57163900	30.72200200	-0.16992400
H	35.72474600	35.33478400	-6.39676200
H	19.06477000	37.92149900	4.93351000
H	33.39885000	40.43939000	0.98139300
H	35.06275200	39.83934500	1.28217100
H	27.65381600	35.32029200	1.72273000
H	32.33089600	35.61953100	1.71006000
H	32.61456200	45.21786700	-4.96493700
H	32.65836600	43.46388800	-4.64217000
H	20.80036800	47.71469000	3.35027500
H	21.08470800	45.96231100	3.15821400
H	19.67889500	44.77707800	3.41256900
H	18.31969800	43.84015900	3.84190500
H	26.86453600	41.63368200	8.20950500
H	25.59498100	40.65071600	8.79561100
H	23.46501200	35.10792300	3.54667700
H	23.21917600	35.29454500	5.28441200
H	29.53616800	39.59103200	0.95723700
O	32.22260500	36.90624300	-1.87025200
H	32.87147700	37.40538000	-1.31754900
H	31.34257600	37.28823100	-1.68555800

TS2 (14.9)

C	-7.39226600	-1.60457200	-8.31100200
C	-6.92734100	-0.47423100	-7.38519000
C	-5.45500800	-0.46543800	-7.08501300
C	-4.53860300	-1.46541500	-7.29308200
C	-4.72840100	0.62854000	-6.48238600
C	-3.36543200	0.23029400	-6.37156500
C	-5.09882100	1.90546800	-6.02792500
N	-3.28388400	-1.05909800	-6.85714000
C	-2.37885400	1.07890900	-5.86061500
C	-4.12282300	2.74889000	-5.50788200
C	-2.77536700	2.34076200	-5.43299100
H	-6.86871400	-1.56628900	-9.27157200
H	-7.48736500	-0.52848000	-6.44108700
H	-7.19862400	0.49145800	-7.83216200
H	-4.67329300	-2.45125700	-7.71218700
H	-6.13376600	2.22962800	-6.08654700
H	-2.44276100	-1.60719500	-6.92418200
H	-1.34228700	0.77172900	-5.78804800
H	-4.39790800	3.73673000	-5.15275100
H	-2.03519100	3.01500300	-5.01538700
C	-3.53532700	-5.62004000	-5.67144500
C	-3.37125600	-4.60682800	-4.52771000
C	-2.02510300	-4.71469800	-3.85052500
C	-0.97083200	-3.85608200	-4.17571900
C	-1.78082800	-5.69739500	-2.87973200
C	0.27702400	-3.95824500	-3.56263100
C	-0.54120700	-5.82131900	-2.25969000
C	0.49820800	-4.94259200	-2.59131400
O	1.68485600	-5.06629100	-1.94053500
H	-2.76583600	-5.47063600	-6.43659800
H	-4.16961400	-4.76015800	-3.79192600
H	-3.50920600	-3.59118300	-4.91629100
H	-1.12924700	-3.05887600	-4.89752600
H	-2.58343000	-6.37353700	-2.59315900
H	1.05923900	-3.24969800	-3.80344300
H	-0.38101600	-6.56209900	-1.48777200
H	2.21160600	-4.24801800	-2.09224700
C	-8.43065400	-2.59884900	-3.06678800
C	-7.44735200	-1.47165100	-3.39177600
C	-7.13214000	-0.63464300	-2.14582500
C	-6.16859100	-2.03768100	-4.01608400
H	-7.98553600	-3.30333400	-2.35253300
H	-7.92352600	-0.80724400	-4.12895000
H	-6.46866700	0.20001700	-2.38594800
H	-8.04601700	-0.22898700	-1.69569700
H	-6.62198300	-1.24110800	-1.38805600
H	-5.63690400	-2.66855600	-3.29532500

H	-6.39504100	-2.64729000	-4.89765300	C	-6.99344100	0.15642200	2.00084600
H	-5.48777500	-1.24381100	-4.32513300	C	-9.49852900	0.27142300	1.70740600
C	-4.30792200	6.63336000	-2.07977800	H	-8.22231500	-2.09237800	0.92422600
C	-2.99764500	5.91506100	-1.73670100	H	-8.46470500	-0.39589300	3.46426800
C	-3.03414900	5.35270200	-0.31080200	H	-6.94295800	1.20666200	2.30513100
C	-2.67651800	4.81737500	-2.75732400	H	-6.15493400	-0.36865800	2.46839300
H	-5.15508100	5.93986500	-2.04237200	H	-6.82982700	0.11798400	0.91905300
H	-2.18606200	6.65514400	-1.78199400	H	-9.50764700	1.32762600	1.99393600
H	-3.87312600	4.65822500	-0.18395300	H	-9.40736000	0.22488900	0.61549500
H	-2.11115200	4.81423400	-0.08357100	H	-10.46601700	-0.16334400	1.98137700
H	-3.14989100	6.15367200	0.42619800	C	-7.52490800	-5.63990600	1.36275100
H	-3.39660000	3.99320100	-2.69874800	C	-6.24963400	-5.58050800	0.56809800
H	-2.70459000	5.20684000	-3.78116000	O	-6.21219500	-5.36835200	-0.63436200
H	-1.68002200	4.40412300	-2.58378000	H	-7.39134000	-6.03382000	2.37341400
C	-8.73182700	5.81734100	0.11577200	N	-5.11591000	-5.80526800	1.31712200
C	-7.76112200	4.75883700	-0.43229300	C	-3.81226900	-5.62192000	0.73720200
C	-7.94329000	4.61245800	-1.95505300	C	-2.97137100	-6.87500000	0.61878800
C	-7.94501600	3.42859400	0.30683000	O	-1.76368500	-6.77391300	0.38038300
C	-6.97779600	3.64296600	-2.64343500	H	-3.22183300	-4.89870100	1.30700000
H	-9.76881400	5.53650900	-0.10497200	H	-3.94648800	-5.21473900	-0.27041900
H	-6.73416900	5.10851900	-0.24511800	H	-5.19331200	-5.79874700	2.32396300
H	-7.84121100	5.60346900	-2.41610200	N	-3.61681700	-8.04575800	0.75443000
H	-8.97517700	4.29027500	-2.15261100	C	-2.93974100	-9.32139200	0.65669400
H	-8.95667200	3.03485700	0.15233500	H	-2.70341600	-9.73158300	1.64541500
H	-7.23984000	2.66298000	-0.02675500	H	-2.00892600	-9.17016600	0.11002600
H	-7.79207000	3.55833700	1.38256700	H	-4.60323300	-8.00195200	0.96667000
H	-7.13988700	3.62007200	-3.72544800	C	-5.00338700	-2.94061100	5.91154300
H	-5.93707900	3.95306200	-2.48831100	C	-4.81305300	-2.22249200	4.56151900
H	-7.09566700	2.61870700	-2.27781500	C	-3.42027200	-1.65702000	4.37391600
C	-7.12655300	6.74848000	3.41795700	C	-2.33193200	-2.51494400	4.15875400
C	-5.67692300	6.84025800	3.89804400	C	-3.17808400	-0.27917500	4.43930100
C	-4.69998300	6.02299600	3.05388900	C	-1.03954300	-2.01049000	4.01865600
S	-5.01133600	4.20945500	3.19618900	C	-1.88516100	0.23038700	4.28991600
C	-3.29570400	3.59516100	3.08764100	C	-0.81077500	-0.63372600	4.07756700
H	-7.48990200	5.71570500	3.44673500	H	-4.30603500	-3.77948300	6.00379300
H	-5.60422700	6.51940000	4.94354400	H	-5.54824900	-1.41619800	4.46684400
H	-5.34896100	7.88746000	3.86404800	H	-5.02474500	-2.92909600	3.74772900
H	-3.66973400	6.20005600	3.37345600	H	-2.49510200	-3.58717600	4.13124200
H	-4.77112200	6.30956700	2.00038800	H	-4.00891800	0.40136100	4.60877800
H	-2.83563000	3.85523500	2.13355200	H	-0.20996400	-2.69395900	3.86623200
H	-2.69560400	3.99270100	3.90970800	H	-1.71975400	1.30194100	4.34563000
H	-3.33762800	2.50828900	3.16166500	H	0.19527900	-0.23948500	3.96673400
C	-8.35847600	-1.96256000	2.00497800	C	-1.06797900	-5.62495900	7.26006200
C	-8.33749500	-0.47459100	2.37453000	C	-1.19885700	-5.56437100	5.74469200

O	-2.30132300	-5.50015300	5.19806100	C	4.52489100	-4.14694900	0.76711800
H	-0.05440900	-5.85122800	7.59932500	C	4.90732200	-4.59631000	-0.64642200
N	-0.02125300	-5.51945400	5.05431600	C	5.12654300	-3.43908900	-1.64999000
C	0.02721700	-5.64798600	3.60974800	C	3.98689000	-2.45941900	-1.49548200
C	0.69522000	-6.98217000	3.23770300	O	2.87908900	-2.66726200	-2.00747200
O	1.50725200	-7.50251800	4.00408700	O	4.20884800	-1.45587700	-0.68648000
H	0.61215000	-4.83072700	3.16886800	H	3.58380000	-3.59435700	0.76816800
H	-0.99184100	-5.58424000	3.22529500	H	4.10002300	-5.23989800	-1.00808500
H	0.80802800	-5.88156600	5.51067900	H	5.81915000	-5.20005200	-0.60227300
N	0.32487800	-7.46674500	2.03828600	H	5.14579200	-3.83234300	-2.66935300
C	0.82050700	-8.70859200	1.48265500	H	6.07211900	-2.93702800	-1.44876500
H	0.06643700	-9.50262200	1.54052400	H	6.50236900	-3.43632700	1.04627100
H	-0.39990900	-6.99552700	1.50241400	C	8.94687600	-5.49183900	-1.71361500
C	3.58431000	-1.18637800	8.55591500	C	8.64248600	-4.13137700	-1.11652900
C	3.33637600	-1.81523200	7.18484400	O	8.11995300	-4.02817800	0.00230000
O	4.21760900	-1.24328400	6.20678300	H	8.00264400	-5.99140800	-1.95142100
H	3.39842400	-0.10896200	8.52524700	H	9.55648200	-5.43767100	-2.61839000
H	2.29023800	-1.64977600	6.88910600	N	8.96580600	-3.05554500	-1.87085000
H	3.49124500	-2.90298900	7.24343000	C	8.62941100	-1.71492900	-1.45163400
H	3.95033600	-1.56184500	5.32901200	C	8.27764200	-0.86772100	-2.68478800
C	0.26412100	7.31558500	6.80049600	O	8.67916200	-1.18724100	-3.80589500
C	-0.50445500	6.82088800	5.57280400	C	9.77155600	-1.05698200	-0.62191900
C	-0.08625600	7.55959400	4.30102000	C	11.03978500	-0.81691500	-1.42408600
S	-0.87784100	6.94389700	2.76619500	O	9.34812300	0.19878100	-0.09281000
C	-0.12018700	5.28996700	2.68238000	H	7.76862800	-1.78089200	-0.78619100
H	-0.04872900	6.78330400	7.70396500	H	9.98115500	-1.75876800	0.19932400
H	-1.58333100	6.94486000	5.72012000	H	9.25658900	-3.14165300	-2.83829800
H	-0.32970800	5.74633100	5.44884300	H	11.79781100	-0.35582300	-0.78564100
H	1.00104000	7.51441700	4.16737300	H	11.43482200	-1.76144400	-1.80723000
H	-0.35922100	8.61782200	4.36476700	H	10.84660900	-0.15366200	-2.27177800
H	-0.41581000	4.83702500	1.73839500	H	8.43279600	0.09339000	0.22147700
H	0.97068900	5.36927200	2.70318200	N	7.55849500	0.23555300	-2.38707700
H	-0.45489000	4.64792400	3.49864500	C	7.19084000	1.28377700	-3.31670000
C	5.66257500	1.43526800	4.74509300	C	8.16359300	2.47272000	-3.36510700
C	5.91963400	0.53471400	3.56616100	O	7.93866100	3.38927500	-4.15127100
O	5.85686000	0.97916700	2.41216100	C	5.75944100	1.79596100	-3.01854800
H	5.14304900	0.90731700	5.54970000	C	4.66272300	1.21406900	-3.93003200
N	6.28308700	-0.73589600	3.86083600	C	4.71194100	1.82179900	-5.33550800
C	6.54462500	-1.73145800	2.84235100	C	4.70704200	-0.31612400	-3.97775200
C	5.28609600	-2.43983200	2.35567300	H	7.23938800	0.85824500	-4.32370100
O	4.16979300	-2.20603500	2.84383200	H	5.75404600	2.88367000	-3.10870800
H	7.02972800	-1.25588400	1.98997900	H	5.52708700	1.56486200	-1.97352800
H	6.10809200	-1.05328900	4.80661900	H	3.70273000	1.50224500	-3.47872800
N	5.54605100	-3.31998000	1.37972400	H	3.89411700	1.43538200	-5.95319700

H	4.62808400	2.91240200	-5.29815400	H	1.05479000	2.85394600	-4.32657200
H	5.65638300	1.58735700	-5.83866600	H	-1.38830400	1.37966100	0.91130400
H	7.18919600	0.31666200	-1.43969400	H	0.11273800	1.93674300	2.71603400
H	3.81880500	-0.72470500	-4.46869400	H	1.28233100	2.76832200	1.55870800
H	5.58730400	-0.67715700	-4.52093000	H	2.00713800	0.83156400	-0.28547600
H	4.74831800	-0.71948400	-2.96735400	H	1.96240300	-0.35404000	-1.58378900
N	9.23536100	2.44139000	-2.52627000	H	0.38479600	-0.57428700	1.03121700
C	9.98420300	3.66802800	-2.28640800	H	1.95359200	-1.20350200	-5.75578700
C	9.60979700	4.27629900	-0.91617700	H	0.28556500	-0.93831500	-6.30486200
C	8.11194300	4.35490100	-0.71288800	H	1.61800500	0.02668800	-7.01705600
C	7.33273000	5.25189400	-1.45488400	H	1.27611700	5.92404800	-1.58524600
C	7.46452100	3.48671400	0.17392600	C	-0.74725700	-2.80537800	0.23593500
C	5.94701100	5.28234100	-1.31045300	N	0.18925400	-1.94287400	-0.47075000
C	6.07708000	3.51624800	0.33049200	C	-2.05312800	-2.06913400	0.58933200
C	5.31660800	4.41539600	-0.41575800	N	-4.44525600	1.77928500	-0.94603400
H	9.72655900	4.34573700	-3.10127600	C	-2.66483700	-1.31414500	-0.56835300
H	10.06682600	5.27060000	-0.83784700	C	-2.51041200	-1.72682000	-1.88862400
H	10.04711900	3.66126000	-0.12162200	C	-2.97779200	-0.95860900	-2.96990700
H	7.81352500	5.91425900	-2.16845600	C	-3.63150200	0.24994000	-2.77379500
H	8.05182500	2.77400700	0.74690100	C	-3.82342400	0.65266000	-1.45146600
H	5.35954600	5.97835100	-1.90202700	C	-4.40405800	1.75070900	0.43440000
H	5.60868700	2.83788700	1.03487500	C	-3.74320200	0.61846800	0.84482900
H	4.23541500	4.43501000	-0.30960500	C	-3.36107300	-0.10718200	-0.33995400
H	9.25311800	1.72188000	-1.80864300	H	-0.94093300	-3.66973500	-0.40206500
C	0.70646100	3.34400900	-1.05577300	H	0.44444700	-2.17758100	-1.42161700
O	0.25122900	4.50136900	-0.45318000	H	3.21680800	-1.38744300	0.05707700
C	-0.25839800	2.19463300	-0.77156600	H	-1.85636400	-1.36642400	1.40505600
C	0.20338900	0.91650700	-1.53084600	H	-2.76104300	-2.79450900	1.01166800
C	0.59373800	1.29052800	-2.94078200	H	-4.92266800	2.47396700	-1.49617100
C	0.82658800	2.56565800	-3.30751600	H	-1.98227500	-2.64411700	-2.11128600
O	0.78676300	3.62129800	-2.47690600	H	-2.80985900	-1.31446300	-3.97908200
C	-0.46978900	1.91871000	0.69461100	H	-3.98273000	0.84766500	-3.60441900
C	0.35606300	2.21967200	1.69724300	H	-4.86599200	2.53873300	1.01113000
C	1.35295300	0.14561200	-0.82988500	H	-3.57556800	0.32018400	1.86829700
C	0.85207500	-0.93007900	0.11677800	H	-0.28723300	-3.19408200	1.15335700
C	0.68422300	0.20219000	-3.91730700	H	-8.46629000	-1.53232900	-8.50332500
O	0.42403300	-0.96134900	-3.66529500	H	-7.20115100	-2.58559300	-7.86282100
O	1.09915500	0.60115700	-5.15125800	H	-9.35663400	-2.21176300	-2.62602100
C	1.24609500	-0.45233100	-6.11233600	H	-8.69673400	-3.16489800	-3.96625600
O	2.29752300	-1.54192500	0.78099500	H	-3.43301400	-6.64512200	-5.30131500
C	1.13673400	5.61712200	-0.54533000	H	-4.51758200	-5.52715100	-6.14790700
H	-1.22288200	2.48834000	-1.20178900	H	-4.27700100	7.06623100	-3.08541500
H	-0.65694500	0.24622900	-1.60013000	H	-4.51545200	7.44207400	-1.37046400
H	1.73637100	3.10834300	-0.74040900	H	-7.79212900	7.35274800	4.04191500

H	-7.21402300	7.10568000	2.38740600
H	-8.54998000	6.79894800	-0.33519200
H	-8.63998600	5.91672000	1.19934800
H	0.09802000	8.38521000	6.96938300
H	1.34213500	7.16327100	6.67901600
H	4.61786700	-1.34976600	8.87469100
H	2.91617800	-1.63640800	9.29580800
H	-6.02375900	-3.32609900	6.00485900
H	-4.82154600	-2.25252900	6.74309300
H	9.45972300	-6.09674000	-0.96308700
H	11.06079300	3.47016600	-2.32718500
H	-3.56343400	-10.03727300	0.11598300
H	5.07396300	2.28934100	4.41094900
H	6.61850900	1.80474700	5.13132600
H	4.35765900	-5.04311900	1.38012900
H	7.24045400	-2.47279400	3.24757100
H	0.67384400	6.42407000	0.02396200
H	2.11323400	5.37518400	-0.10406700
H	-9.30725900	-2.43378600	2.28516100
H	-7.54819600	-2.50086000	2.51037500
H	-7.94784200	-4.63375500	1.43135600
H	-8.24887000	-6.25872000	0.82785100
H	-1.37115100	-4.65721500	7.67070800
H	-1.75966500	-6.37864100	7.64152300
H	1.10022200	-8.56654700	0.43502000
H	1.69606200	-9.01988400	2.05395900
H	2.55602900	-1.31592500	1.69081300
O	6.46634100	-0.14811100	0.12593200
H	6.16398000	0.33129700	0.93224300
H	5.70031700	-0.68538300	-0.18057600

Int4 (0.6)

C	17.59398200	43.88500200	-6.44712500
C	18.91512600	44.58035800	-6.07550300
C	20.03850200	43.61983400	-5.78449200
C	19.93947800	42.27860400	-5.50413300
C	21.45082900	43.93118300	-5.75536400
C	22.15056300	42.72377500	-5.46540100
C	22.19542600	45.10299400	-5.97246300
N	21.19973700	41.73853100	-5.29672800
C	23.54612400	42.65891200	-5.42293000
C	23.58498000	45.04360000	-5.92919900
C	24.25319300	43.83086100	-5.66488100
H	17.72491700	43.31684000	-7.37532900
H	18.75110900	45.22776700	-5.20244200
H	19.22314800	45.25219400	-6.88707500
H	19.06771700	41.64618100	-5.43247400
H	21.69113900	46.04060400	-6.18710000
H	21.38769700	40.76431600	-5.12938400
H	24.06695200	41.73262100	-5.21032900
H	24.16816400	45.94106900	-6.10936400
H	25.33777800	43.80865300	-5.63594100
C	19.01581100	39.17826000	-2.73327200
C	19.85794700	40.18190400	-1.92158600
C	21.15614100	39.57676700	-1.41640000
C	22.33250000	39.60684700	-2.17525300
C	21.22990200	38.95040800	-0.16256400
C	23.53596400	39.06397500	-1.71532900
C	22.41601400	38.39752000	0.31713900
C	23.58495700	38.46260200	-0.45174900
O	24.73083200	37.96066400	0.09937400
H	19.55490200	38.94752400	-3.66422600
H	19.28209800	40.55499000	-1.06728200
H	20.08673200	41.04854200	-2.54358100
H	22.32298400	40.10184000	-3.14394700
H	20.33956900	38.90851600	0.46127000
H	24.44252200	39.16836800	-2.30060200
H	22.46330400	37.95212500	1.30319300
H	25.50291200	38.02124800	-0.50664500
C	18.62693900	45.31684100	-1.30405500
C	19.92505600	45.04239700	-2.07518000
C	21.08534100	45.84316600	-1.47387900
C	20.23912600	43.54992100	-2.10685200
H	18.74694400	45.03939300	-0.24935300
H	19.78587100	45.37803900	-3.11300000
H	22.00825300	45.69248400	-2.04065300
H	20.86582600	46.91729400	-1.45255600
H	21.27849600	45.51975800	-0.44505000
H	20.37526700	43.15512400	-1.09310200
H	19.41507600	43.00495700	-2.57402800
H	21.14660800	43.34147700	-2.67991200
C	27.28114400	48.52787500	-5.54691600
C	28.19211900	47.30370400	-5.39939100
C	28.73034200	47.20749100	-3.96546100
C	27.47567600	46.01292700	-5.81318400
H	26.36672500	48.39464400	-4.95532000
H	29.05233100	47.44179800	-6.06985000
H	27.91474600	47.02052300	-3.25430900

H	29.45526700	46.39779400	-3.86555300	H	22.70949200	49.36130600	2.71129300
H	29.22262300	48.13841100	-3.66352200	C	19.41399200	43.82106500	3.87600200
H	26.65825700	45.78666900	-5.11599000	C	19.91456200	42.68834500	3.02550400
H	27.03989700	46.09908900	-6.81461200	O	19.40056400	42.37215100	1.95698800
H	28.16259900	45.16172500	-5.82009700	H	19.76690000	43.80631300	4.90879600
C	24.77606000	51.75297300	-2.65202600	N	20.99588700	42.03845000	3.54598600
C	24.57327300	50.22435000	-2.66725700	C	21.66305000	40.99188500	2.82102600
C	23.63066000	49.78318800	-3.79931800	C	21.53019900	39.60836300	3.44621900
C	24.09854800	49.74027300	-1.29047400	O	22.48907700	38.83817200	3.50468500
C	23.46150900	48.26682200	-3.93952800	H	22.73414700	41.19190500	2.73128100
H	23.82339500	52.28004000	-2.53105600	H	21.23845000	40.96871300	1.81306400
H	25.55751700	49.76744500	-2.86132100	H	21.39003200	42.34523800	4.42253500
H	24.00584400	50.19086800	-4.74683200	N	20.29643800	39.27798700	3.89443500
H	22.64583300	50.24289000	-3.63717000	C	20.04492500	37.96005200	4.44700000
H	23.10993600	50.15215100	-1.05436900	H	20.81433600	37.72522300	5.18622800
H	24.02354600	48.65094600	-1.22833800	H	20.07538900	37.18771700	3.66943800
H	24.78796100	50.06067400	-0.50289100	H	19.53615200	39.92301600	3.74013300
H	22.82878700	48.01183500	-4.79439100	C	24.72115400	44.88201500	6.12203800
H	24.42790400	47.77413800	-4.10783100	C	25.04482900	44.66180800	4.62911100
H	22.99833300	47.81870000	-3.05548400	C	26.48015200	44.28915700	4.32672400
C	27.87196300	52.02405700	-0.48624200	C	26.91161700	42.96880500	4.51968100
C	29.21885900	51.36316300	-0.80318600	C	27.39412600	45.21715300	3.81478100
C	29.14225800	49.84968700	-0.99121000	C	28.21078700	42.58028100	4.19674900
S	28.76977800	48.98873800	0.59510700	C	28.69770400	44.83168900	3.48703500
C	29.59304300	47.39603500	0.25558300	C	29.10971800	43.51020000	3.66768000
H	27.43752900	51.62350700	0.43617500	H	25.11673600	44.05000300	6.71301000
H	29.95000000	51.58687800	-0.01803300	H	24.77753100	45.55723200	4.06246500
H	29.61634400	51.79135200	-1.73257300	H	24.40100100	43.85617600	4.25519100
H	30.10285700	49.46554000	-1.34210300	H	26.20362200	42.24572300	4.90609700
H	28.37794400	49.58853500	-1.73245400	H	27.07967000	46.24496400	3.65274000
H	29.09958900	46.85550200	-0.55186700	H	28.51220800	41.54715800	4.34993100
H	30.64121200	47.55514800	-0.00159200	H	29.39298200	45.56518600	3.08947500
H	29.52851400	46.80450800	1.17115000	H	30.11870900	43.21012600	3.39971500
C	21.29405300	46.95201300	2.73895100	C	26.40688700	40.65001000	8.06103300
C	22.80436800	47.17798300	2.61734700	C	25.76499400	40.48528900	6.68709200
C	23.44454200	46.09542900	1.74177400	O	24.88101700	41.24257200	6.29373800
C	23.11439600	48.57401200	2.06625900	H	27.12846000	39.87052100	8.31378700
H	20.82779500	46.98207500	1.74573000	N	26.26178700	39.46442700	5.91328400
H	23.23797300	47.10858300	3.62584400	C	25.52002800	38.92399200	4.78093500
H	24.53250800	46.18821700	1.71518300	C	24.64477700	37.72855100	5.23387100
H	23.20301200	45.08823900	2.09799600	O	24.21004300	37.65674800	6.37482100
H	23.09051100	46.17047800	0.70839400	H	26.20637600	38.64344300	3.97570700
H	24.19383100	48.73330400	1.97268100	H	24.84088900	39.69830500	4.41687800
H	22.67483500	48.69825000	1.07008900	H	26.83797800	38.79262600	6.40098100

N	24.43789800	36.77978100	4.27652700	H	27.69836700	35.98140900	-2.48065800
C	23.31797300	35.84497900	4.34301100	H	29.29048700	35.86531700	-1.74271700
H	22.37935800	36.37480100	4.15728700	H	30.04614700	34.93698300	0.66823400
H	24.65215700	37.07620300	3.33170000	C	29.29898100	31.23699100	-1.09699600
C	32.73989800	40.47099900	6.36295900	C	30.13327200	32.49886800	-1.20678400
C	32.24010600	40.68475200	4.93639300	O	30.32004600	33.22875100	-0.22457700
O	31.91115500	39.40434800	4.36966800	H	28.24213500	31.51242900	-1.02181100
H	33.53684900	39.72086100	6.36244700	H	29.42845700	30.56236400	-1.94618100
H	33.01271700	41.17425700	4.32582900	N	30.65371200	32.76661900	-2.42612000
H	31.35948100	41.34046100	4.93673800	C	31.41901900	33.96292000	-2.68101700
H	31.14239000	39.48603400	3.78874500	C	31.10399500	34.47821700	-4.09336200
C	34.70898500	47.89981500	1.13604400	O	30.63553600	33.72237000	-4.94902700
C	33.62403800	47.82212100	0.05267800	C	32.94919800	33.71500800	-2.50644300
C	33.90327100	48.76413800	-1.11811400	C	33.51237300	32.71829100	-3.50643700
S	32.58397400	48.85338400	-2.38998700	O	33.68663500	34.92611000	-2.65763800
C	32.51460600	47.12229000	-2.96442300	H	31.13700500	34.71270800	-1.93944800
H	34.58316100	47.08151300	1.85349300	H	33.07195700	33.32421200	-1.48530600
H	32.64983200	48.06762400	0.48842300	H	30.36570100	32.25991300	-3.25557500
H	33.55225400	46.79413100	-0.32104600	H	34.58236300	32.58426600	-3.32887600
H	34.84648800	48.49696000	-1.60845700	H	33.01768100	31.74926800	-3.40270100
H	34.00482800	49.79562400	-0.76460300	H	33.37018500	33.07202300	-4.53139700
H	31.83906500	47.10349900	-3.82065200	H	33.26593000	35.61411900	-2.11654700
H	33.50312600	46.77708400	-3.28018600	N	31.43444200	35.77289900	-4.28523100
H	32.10801900	46.45361300	-2.20498100	C	31.38097200	36.46296900	-5.56001200
C	34.00188700	39.69593300	1.51293400	C	32.71725300	36.51163700	-6.31993400
C	33.47689600	38.39194900	0.95324300	O	32.75714200	37.08789100	-7.40509000
O	33.72625300	38.05441700	-0.21472700	C	30.83381700	37.90345600	-5.38455700
H	33.82871000	39.79597300	2.58557800	C	29.38986700	38.09401400	-5.87895500
N	32.67467800	37.67694200	1.77321000	C	29.28252800	37.93066500	-7.39923200
C	31.89733900	36.54146400	1.30841600	C	28.41096000	37.17711600	-5.13974500
C	30.41286000	36.74972900	1.56220800	H	30.72047900	35.87944500	-6.20743900
O	29.98317400	37.79232300	2.08456900	H	31.48341500	38.59247000	-5.92657700
H	32.02227800	36.47880300	0.22601900	H	30.89619500	38.17221800	-4.32478900
H	32.47809400	38.05210400	2.69506500	H	29.11341500	39.12965100	-5.63634200
N	29.62853900	35.76544000	1.09259600	H	28.27193200	38.17608300	-7.74448400
C	28.19116700	35.92286200	0.98310600	H	29.99622900	38.57704500	-7.91811800
C	27.63549800	35.49642600	-0.38075300	H	29.49777000	36.90311300	-7.71039000
C	28.26996300	36.21053900	-1.57513600	H	31.72303200	36.32059400	-3.47214000
C	28.29478000	37.72171000	-1.39431000	H	27.39474700	37.29361300	-5.52702600
O	27.21252400	38.29166300	-1.04366700	H	28.68326900	36.12174900	-5.25017700
O	29.38522800	38.33173500	-1.58983900	H	28.40505800	37.41508500	-4.07400400
H	27.96315000	36.97102700	1.18063400	N	33.79340700	35.90866300	-5.74403800
H	26.55972800	35.69832700	-0.35791100	C	35.13002700	36.23802700	-6.22479900
H	27.76290900	34.41519700	-0.50291200	C	35.84822400	37.17179500	-5.22637200

C	34.99037300	38.34872300	-4.80345500	H	25.26452600	38.76869200	-6.20853400
C	34.54196700	39.28986600	-5.74128900	H	31.62045000	43.78761800	-6.25767800
C	34.58963700	38.49647000	-3.47035500	C	26.57760300	40.89857400	1.16068600
C	33.71519700	40.34402000	-5.35575500	N	27.41310500	40.70568300	-0.03478200
C	33.76954500	39.55586300	-3.07603000	C	26.08567900	42.34423600	1.32600800
C	33.32741500	40.47919800	-4.02095800	N	25.53488400	46.11548100	-1.59509800
H	34.98898100	36.72895300	-7.18897900	C	25.44976400	42.94273500	0.08839600
H	36.78386300	37.51802300	-5.68423600	C	24.55752500	42.21893600	-0.69677800
H	36.12574900	36.59828300	-4.33502200	C	23.97194800	42.76236400	-1.85360900
H	34.81379900	39.17836400	-6.78610000	C	24.23842700	44.06224300	-2.26026700
H	34.91647900	37.77849500	-2.72383300	C	25.11174300	44.80565100	-1.46464300
H	33.36555100	41.05273700	-6.10142700	C	26.39336100	46.42845900	-0.55854900
H	33.48545600	39.64252400	-2.03318600	C	26.56021700	45.32393300	0.24262400
H	32.68063800	41.29789100	-3.71736300	C	25.74084100	44.27028500	-0.30204300
H	33.70018000	35.59051400	-4.78254500	H	25.75061100	40.19363700	1.05114700
C	30.03431700	43.32151900	-4.13681600	H	27.31702200	39.76661600	-0.54525700
O	30.79477500	44.42864700	-4.44868000	H	30.26521700	39.50453100	-0.43042300
C	28.94311700	43.67573700	-3.13120600	H	26.90923500	42.97502200	1.66786100
C	28.12040100	42.39667100	-2.82656200	H	25.37622800	42.33728300	2.16179200
C	27.76985300	41.70250700	-4.11763600	H	25.20737100	46.76216300	-2.29485000
C	28.40660200	41.99334400	-5.27000400	H	24.30604800	41.19830700	-0.43331900
O	29.43455200	42.85210900	-5.37806700	H	23.29947200	42.14745600	-2.43735000
C	29.41307100	44.32007600	-1.85614500	H	23.79217700	44.47475800	-3.15669000
C	30.65540200	44.34947500	-1.37298400	H	26.79557000	47.42603100	-0.44775200
C	28.85441800	41.42487900	-1.83821500	H	27.15529700	45.28555100	1.14184800
C	28.22792200	41.57181200	-0.50376300	H	27.15190200	40.59394600	2.04231600
C	26.69135800	40.71269000	-4.05153600	H	16.79250000	44.60999300	-6.62309700
O	26.15200600	40.38830000	-3.00679500	H	17.36386400	43.15234500	-5.66643700
O	26.34824700	40.18108500	-5.25078700	H	18.42045700	46.39116600	-1.35431000
C	25.34292700	39.15796600	-5.19440500	H	17.78086100	44.74151100	-1.69372500
O	30.65901300	39.99431100	0.31559700	H	19.01192800	38.23042900	-2.18425700
C	31.92794500	44.15087100	-5.27317400	H	18.03104400	39.53857700	-3.04743700
H	28.26824700	44.37364300	-3.63669400	H	26.97419100	48.70098900	-6.58380700
H	27.17762000	42.71430300	-2.37031100	H	27.79496600	49.40637100	-5.14285900
H	30.66476500	42.49023100	-3.78137100	H	28.01754600	53.10050500	-0.34824000
H	28.12903200	41.55725300	-6.22245200	H	27.17599100	51.81298900	-1.30458000
H	28.60438100	44.74644600	-1.26479100	H	25.25400400	52.09521100	-3.57581200
H	30.86423500	44.79755500	-0.40665900	H	25.41479600	51.98486800	-1.79339400
H	31.50537700	43.94683800	-1.91368100	H	34.62254000	48.82957600	1.70822300
H	29.91187100	41.68661700	-1.75539500	H	35.70662000	47.78957500	0.69829600
H	28.78532100	40.39160400	-2.17107300	H	31.92267400	40.09421800	6.98673000
H	28.37122800	42.50170900	0.04192200	H	33.13798100	41.38125500	6.82351400
H	25.63613300	38.37055200	-4.49877600	H	23.63383200	44.86023000	6.25090400
H	24.38473900	39.57396800	-4.87178500	H	25.16667200	45.81305700	6.48742600

H	29.57167100	30.72196100	-0.16995600
H	35.72477600	35.33479600	-6.39672100
H	19.06477000	37.92150000	4.93351000
H	33.39888600	40.43941200	0.98138400
H	35.06275600	39.83934000	1.28218300
H	27.65380500	35.32038200	1.72279400
H	32.33068200	35.61930100	1.70976500
H	32.46816300	45.09182200	-5.37930100
H	32.58318900	43.40800600	-4.80018700
H	20.80035800	47.71468800	3.35026900
H	21.08470800	45.96231100	3.15821400
H	19.67889800	44.77707300	3.41255900
H	18.31969800	43.84015900	3.84190800
H	26.86453500	41.63368400	8.20950200
H	25.59498100	40.65071000	8.79561100
H	23.46501100	35.10792300	3.54667800
H	23.21917700	35.29454600	5.28441200
H	30.47683600	39.39077600	1.05715900
O	31.77837500	37.07640900	-1.84366500
H	32.44478200	37.61550400	-1.36943000
H	30.90363600	37.53278400	-1.75913200

Int5 (2.3)

C	17.59397100	43.88498400	-6.44711100
C	18.92206500	44.55360900	-6.06611700
C	20.00370700	43.55269600	-5.76061900
C	19.87155000	42.18731700	-5.67035500
C	21.39799600	43.83686700	-5.51315700
C	22.05492800	42.59178800	-5.29449700
C	22.15732700	45.01742900	-5.45675000
N	21.09718500	41.60600400	-5.38916600
C	23.42850200	42.50340400	-5.05330800
C	23.52206600	44.93475300	-5.20509700
C	24.15746900	43.69042500	-5.01114400
H	17.71946500	43.31754100	-7.37686900
H	18.76826500	45.20187400	-5.19213000
H	19.25877600	45.21877400	-6.87235700
H	18.99858100	41.56479000	-5.79214000
H	21.68377400	45.98255000	-5.60836100
H	21.25631500	40.61980400	-5.26643600
H	23.90697400	41.54065400	-4.90341800
H	24.11624200	45.84065000	-5.16898600
H	25.22523300	43.66064500	-4.81543200
C	19.01581600	39.17825700	-2.73328500
C	19.86957900	40.18799600	-1.93914100
C	21.19470500	39.61391800	-1.46156200
C	22.34407800	39.63739500	-2.26190400
C	21.32601000	39.03183900	-0.19116800
C	23.56373700	39.10810500	-1.83221000
C	22.53937100	38.52035900	0.26595600
C	23.67967900	38.56408800	-0.54715600
O	24.83675100	38.06345100	-0.02683400
H	19.54815100	38.94183300	-3.66713200
H	19.30851600	40.55656500	-1.07294300
H	20.06838000	41.05363500	-2.57230300
H	22.29619800	40.10291200	-3.24327700
H	20.45925900	38.99494000	0.46572400
H	24.43598300	39.15584100	-2.47330100
H	22.62989600	38.12065900	1.26848400
H	25.62817700	38.22963600	-0.57993800
C	18.62694000	45.31684600	-1.30403000
C	19.92861200	45.02282100	-2.06499700
C	21.09881000	45.82243200	-1.48198400
C	20.23265800	43.52774400	-2.06159300
H	18.73960900	45.04188800	-0.24762300
H	19.79764000	45.33764400	-3.10981500
H	22.01829700	45.64557600	-2.05154800
H	20.90041900	46.90053700	-1.49102500
H	21.28213300	45.52983800	-0.44132200
H	20.32542900	43.14493400	-1.03818700
H	19.42394100	42.98070600	-2.55141900
H	21.15782600	43.30039300	-2.60135800
C	27.28121900	48.52804600	-5.54662200
C	28.24112000	47.33073100	-5.49337200
C	28.67030100	47.02223000	-4.05446000
C	27.63851500	46.09872500	-6.17460700
H	26.38123600	48.34561900	-4.94865600
H	29.14201200	47.61462800	-6.05687500
H	27.81486500	46.67576700	-3.46306400
H	29.42956800	46.23457200	-4.02785800
H	29.08806300	47.90641400	-3.56186900
H	26.77507100	45.72395100	-5.61383900
H	27.30651100	46.33183000	-7.19198100
H	28.37149600	45.28997400	-6.23909600
C	24.77589600	51.75297000	-2.65199200
C	24.53880600	50.23387400	-2.68314200
C	23.59634300	49.84705800	-3.83399500
C	24.04387400	49.73504800	-1.31875200

C	23.36959900	48.34182700	-3.98989700	H	22.73153400	41.17959900	2.74319900
H	23.83151500	52.29596500	-2.53067300	H	21.25999200	40.97819800	1.80479200
H	25.50950500	49.75061600	-2.87873800	H	21.36472800	42.32869300	4.43178700
H	24.01000200	50.24768500	-4.76873900	N	20.28692500	39.28124000	3.90058000
H	22.63058900	50.35110800	-3.68762100	C	20.04492200	37.96005500	4.44700000
H	23.07387800	50.18254700	-1.06830300	H	20.81678400	37.72638700	5.18413100
H	23.92321300	48.64793800	-1.29775100	H	20.07633200	37.19087100	3.66636000
H	24.75137900	49.99930600	-0.52612400	H	19.52330700	39.92253400	3.74856600
H	22.81899600	48.11881100	-4.90903000	C	24.72112600	44.88200000	6.12205100
H	24.32410500	47.80566700	-4.03630300	C	25.02029200	44.65737500	4.62805200
H	22.80036100	47.91999300	-3.15696400	C	26.47899700	44.78602700	4.26054000
C	27.87194400	52.02407800	-0.48617000	C	27.41509700	43.84032300	4.70518100
C	29.15632900	51.22187000	-0.70790600	C	26.93598200	45.86793400	3.49702400
C	28.87250100	49.74620500	-1.01502100	C	28.77099100	43.98991600	4.41002600
S	28.09530100	48.87171900	0.40998000	C	28.29106900	46.01949500	3.19440100
C	27.72982100	47.27934200	-0.40278000	C	29.21455300	45.08129700	3.65801600
H	27.36899100	51.66851100	0.42050500	H	25.13226600	44.05437400	6.70698500
H	29.81997900	51.29867000	0.16188200	H	24.44282000	45.36368800	4.02514000
H	29.71475500	51.62552900	-1.56147500	H	24.66849500	43.65490500	4.36995200
H	29.79300100	49.21719200	-1.27379000	H	27.06427000	42.99129600	5.28508200
H	28.18533400	49.67096600	-1.86623100	H	26.22245500	46.60967900	3.14922200
H	26.97498500	47.39966500	-1.18378100	H	29.48593100	43.25664300	4.77564900
H	28.63721300	46.85258500	-0.83905900	H	28.62577600	46.87930200	2.62023700
H	27.34493100	46.60293200	0.36376500	H	30.27232700	45.21691800	3.45549300
C	21.29408300	46.95193400	2.73896300	C	26.40689300	40.65000700	8.06104700
C	22.78232900	47.32950800	2.62761500	C	25.88666100	40.54740100	6.62680800
C	23.50482800	46.54540400	1.53126000	O	25.27093500	41.46443800	6.08528000
C	22.92562100	48.83638300	2.37111500	H	27.12276200	39.86813700	8.32324500
H	20.82288900	46.98754400	1.74839200	N	26.21429600	39.38794100	5.97675100
H	23.26313100	47.10865700	3.59116100	C	25.52001300	38.92401600	4.78091100
H	24.56580600	46.81331500	1.48232600	C	24.55937100	37.78223300	5.19136100
H	23.44790800	45.46974000	1.69038200	O	24.01476500	37.77809500	6.28771200
H	23.06584000	46.76445800	0.55158600	H	26.23551500	38.58589600	4.02619000
H	23.97891900	49.12422100	2.28880100	H	24.92357400	39.74398000	4.37653000
H	22.43444900	49.11568400	1.43126700	H	26.54269400	38.64340800	6.57629800
H	22.47388300	49.42807600	3.17441000	N	24.43713900	36.77738600	4.28178300
C	19.41399900	43.82107300	3.87601200	C	23.31797500	35.84497700	4.34300300
C	19.92197400	42.70006600	3.01399200	H	22.38057100	36.37582000	4.15627400
O	19.42476600	42.41238400	1.92940900	H	24.72711600	37.01093900	3.33983400
H	19.76745000	43.80357900	4.90850700	C	32.73997900	40.47096600	6.36297000
N	20.99074900	42.03829000	3.54117100	C	32.25384400	40.67464300	4.93027100
C	21.66305900	40.99188100	2.82102100	O	31.95170600	39.39229600	4.35035000
C	21.51590000	39.60821300	3.43711700	H	33.53776000	39.72166000	6.36988400
O	22.46769100	38.82765500	3.48587000	H	33.02746900	41.17163300	4.32812100

H	31.36529700	41.31900500	4.91323600	C	31.41900900	33.96300800	-2.68095700
H	31.11894800	39.43263700	3.86159700	C	31.09229900	34.47575100	-4.09254100
C	34.70898100	47.89997400	1.13594000	O	30.61071600	33.72040900	-4.94073700
C	33.58335600	47.67539600	0.11771800	C	32.94806800	33.68796100	-2.53178200
C	33.52023100	48.72325900	-0.99235800	C	33.47697900	32.67638100	-3.53580300
S	32.23751100	48.37922600	-2.26541400	O	33.70349700	34.88528200	-2.69765900
C	33.10089700	47.12643100	-3.27695800	H	31.15855600	34.71415000	-1.93470900
H	34.64450400	47.10238300	1.88817700	H	33.07999800	33.29826800	-1.51099600
H	32.61689300	47.65973100	0.63438200	H	30.34640400	32.27262200	-3.24987600
H	33.69960300	46.68009200	-0.32559900	H	34.54793600	32.52753900	-3.37618600
H	34.48376800	48.82590900	-1.50413200	H	32.97034500	31.71519300	-3.41764300
H	33.26688800	49.70712000	-0.58474200	H	33.32232900	33.02672300	-4.56003500
H	32.40125600	46.81394200	-4.05509600	H	33.27037000	35.58870000	-2.18400500
H	33.98566800	47.55931600	-3.75087800	N	31.43002700	35.76674100	-4.28980000
H	33.37589000	46.25146800	-2.68709300	C	31.38098500	36.46294800	-5.56000700
C	34.00199400	39.69600200	1.51295900	C	32.70854900	36.46898800	-6.34087400
C	33.49973000	38.38195100	0.95920800	O	32.74343300	37.01285900	-7.44289300
O	33.79059500	38.03361900	-0.19365300	C	30.94217500	37.94103600	-5.34977900
H	33.82840600	39.80088100	2.58501800	C	29.53448200	38.28451000	-5.86590200
N	32.67047400	37.68281400	1.76716300	C	29.42215400	38.10849600	-7.38477900
C	31.89697100	36.54103600	1.30792700	C	28.44665100	37.50741100	-5.11777300
C	30.41418700	36.75333900	1.54804300	H	30.67151500	35.92934800	-6.19737600
O	29.99335200	37.83123300	2.01577100	H	31.66024200	38.59268700	-5.85007700
H	32.02742000	36.47196700	0.22475000	H	30.99695300	38.17072700	-4.28095600
H	32.47386500	38.06320500	2.68698800	H	29.38946200	39.35302800	-5.64400800
N	29.62888900	35.75643600	1.12281800	H	28.45568200	38.47529700	-7.74875400
C	28.19131400	35.92294200	0.98317300	H	30.22181200	38.64230600	-7.90584100
C	27.67068700	35.55327600	-0.41018200	H	29.50282000	37.05538800	-7.67325000
C	28.40804800	36.25970200	-1.55144500	H	31.76099400	36.30061900	-3.48516100
C	28.51876100	37.76892000	-1.33827500	H	27.44699600	37.77635300	-5.47726400
O	27.52699400	38.39245800	-0.89796700	H	28.56545700	36.42611100	-5.25302900
O	29.64684300	38.31115500	-1.62437400	H	28.49757200	37.72344200	-4.04837000
H	27.97202600	36.96978900	1.19243900	N	33.79400200	35.90478000	-5.74455400
H	26.60988600	35.82238900	-0.42848600	C	35.13002700	36.23803000	-6.22477100
H	27.74028800	34.46970500	-0.55723000	C	35.85742100	37.14600000	-5.21113000
H	27.87387600	36.08782400	-2.49248300	C	35.03389500	38.34710300	-4.79550800
H	29.40921200	35.84768400	-1.68011500	C	34.65399500	39.31683300	-5.73396300
H	30.04656400	34.91802300	0.71608100	C	34.60455800	38.49710900	-3.47202100
C	29.29900100	31.23698700	-1.09701200	C	33.87234800	40.40657900	-5.35629800
C	30.13147800	32.50165800	-1.19979500	C	33.82156800	39.58822200	-3.08809400
O	30.32613200	33.22396200	-0.21291400	C	33.45286800	40.54585300	-4.03099700
H	28.24095000	31.50846300	-1.02445700	H	34.99223800	36.73925500	-7.18395100
H	29.43424300	30.56535400	-1.94750600	H	36.81027700	37.46511800	-5.65304900
N	30.64297100	32.77489400	-2.42055100	H	36.10343400	36.55784400	-4.31997200

H	34.95460400	39.20577300	-6.77136600	C	23.73705800	43.17060500	0.26276300
H	34.88148400	37.75688200	-2.72731300	C	24.56352000	42.48947700	-0.63123200
H	33.58400000	41.14480800	-6.09926000	C	25.59122600	41.66854100	-2.45223700
H	33.50440700	39.67039800	-2.05351000	C	26.21879600	41.07911200	-1.38511400
H	32.84536500	41.39615400	-3.74321000	C	25.58924900	41.59338900	-0.19882700
H	33.70155300	35.58030400	-4.78484100	H	28.84735200	40.70257300	2.64397700
C	32.14757600	43.68406300	-1.90198200	H	29.58436900	40.84304300	0.48423200
O	33.33213300	44.19201300	-1.41143700	H	30.30042800	39.32845800	-0.58544200
C	30.94951600	44.48431000	-1.39729500	H	26.55597400	40.06239700	2.65944300
C	29.65221000	43.89015200	-2.01951500	H	27.12010200	39.69259200	1.02885200
C	29.85472300	43.63449000	-3.49630900	H	23.99708400	43.05091400	-2.61377800
C	31.08774100	43.59798400	-4.03853400	H	25.13207900	41.96835400	3.13744400
O	32.23177500	43.75451100	-3.35350500	H	23.34604300	43.47309700	2.34623200
C	30.83553500	44.54994100	0.10373200	H	22.94864000	43.82832800	-0.08539100
C	31.54571800	43.86058600	0.99640100	H	25.78804800	41.56813400	-3.50646800
C	29.18857600	42.56689200	-1.37837400	H	26.99002500	40.32333900	-1.42418800
C	28.66212100	42.58403000	0.00877100	H	27.94010200	42.23194700	2.55592100
C	28.66242900	43.35972200	-4.31162000	H	16.79251000	44.60999800	-6.62311400
O	27.52001400	43.52561000	-3.91174100	H	17.36386200	43.15234900	-5.66643500
O	28.95218500	42.86409200	-5.53608000	H	18.42045400	46.39116400	-1.35432500
C	27.82528900	42.44555500	-6.32540500	H	17.78086200	44.74151100	-1.69372900
O	30.72103100	39.82996700	0.17987500	H	19.01192600	38.23043000	-2.18425500
C	34.46773600	43.35896900	-1.67249900	H	18.03104200	39.53857600	-3.04742800
H	31.06332500	45.50731000	-1.78046600	H	26.97413300	48.70083000	-6.58381600
H	28.84635000	44.62373500	-1.90707000	H	27.79501600	49.40642600	-5.14304300
H	32.04941700	42.61423000	-1.65800900	H	28.01753900	53.10051300	-0.34829600
H	31.25831100	43.44470200	-5.09756300	H	27.17599800	51.81290300	-1.30456600
H	30.04785900	45.21141900	0.46419300	H	25.25400100	52.09521800	-3.57581000
H	31.33020600	43.94435100	2.05538900	H	25.41494500	51.98486100	-1.79350200
H	32.35395600	43.19581700	0.71456600	H	34.62251500	48.82954200	1.70827300
H	29.94906100	41.78733700	-1.47279800	H	35.70661800	47.78944600	0.69832300
H	28.31597100	42.22723000	-1.95531400	H	31.92265700	40.09424700	6.98672600
H	27.97960100	43.36617500	0.33824400	H	33.13793100	41.38126900	6.82353000
H	27.29877600	41.63011500	-5.82321600	H	23.63383000	44.86026500	6.25089600
H	27.13456200	43.27286800	-6.49375800	H	25.16669800	45.81304400	6.48742800
H	28.24612700	42.09578300	-7.26683900	H	29.57165400	30.72197400	-0.16994400
H	34.66062100	43.27542800	-2.74508100	H	35.72477200	35.33479700	-6.39674000
C	28.16914300	41.30266600	2.03117500	H	19.06477300	37.92150000	4.93351300
N	28.87602400	41.58976100	0.78863100	H	33.39884500	40.43939100	0.98140100
C	26.88260200	40.50623700	1.71933000	H	35.06275200	39.83934700	1.28217200
N	24.59698800	42.51279900	-2.00639800	H	27.65375600	35.32036900	1.72274800
C	25.79239300	41.39539100	1.18310800	H	32.33087300	35.61952900	1.71008600
C	24.98448600	42.10511700	2.06924000	H	35.31471700	43.83204900	-1.17551800
C	23.97226300	42.97048900	1.61751200	H	34.31289000	42.35295900	-1.26165400

H	20.80035700	47.71471300	3.35023900
H	21.08469800	45.96232400	3.15824000
H	19.67890100	44.77706800	3.41255200
H	18.31969800	43.84015700	3.84190800
H	26.86452100	41.63369100	8.20950000
H	25.59498400	40.65069600	8.79561400
H	23.46500600	35.10792500	3.54667400
H	23.21917500	35.29455200	5.28441500
H	30.64475500	39.18523400	0.91501600
O	31.92404700	36.99292600	-1.84376200
H	32.58506500	37.54399900	-1.37768400
H	31.04420700	37.46444800	-1.78870300

TS3 (16.0)

C	-7.38683900	-1.21036100	-8.55908100
C	-6.93169200	-0.46126700	-7.29993400
C	-5.47817900	-0.63593600	-6.95977200
C	-4.56493200	-1.44848800	-7.58581500
C	-4.76462200	0.02276900	-5.88931500
C	-3.42011900	-0.44713600	-5.91985100
C	-5.12968800	0.95763800	-4.90514800
N	-3.32705100	-1.33508900	-6.97081000
C	-2.45437000	-0.02163000	-5.00244100
C	-4.17909700	1.37631600	-3.98178500
C	-2.85455600	0.89622100	-4.03427200
H	-6.82275600	-0.88144300	-9.43768200
H	-7.54260200	-0.78569600	-6.44557200
H	-7.14513500	0.60976100	-7.41898700
H	-4.69386100	-2.10875300	-8.43002200
H	-6.14315600	1.34299800	-4.85839900
H	-2.49623300	-1.83433500	-7.24042000
H	-1.42733700	-0.36600100	-5.03480100
H	-4.45143300	2.09134300	-3.21422600
H	-2.11585900	1.26183300	-3.32955100
C	-3.85330300	-5.41661100	-5.76832800
C	-3.73288800	-4.20664200	-4.82091800
C	-2.46387500	-4.15308700	-3.99438000
C	-1.33028300	-3.43510700	-4.39514500
C	-2.38592100	-4.83017300	-2.76707200
C	-0.16670100	-3.38731000	-3.61920800
C	-1.24300300	-4.79479200	-1.97989100
C	-0.11322400	-4.07452500	-2.39528500
O	0.96660300	-4.10068000	-1.58189600
H	-3.03769800	-5.42789700	-6.49968000
H	-4.58759700	-4.22302600	-4.13811000
H	-3.82784800	-3.28628900	-5.40445200
H	-1.35168400	-2.89375300	-5.33746400
H	-3.24743900	-5.39688500	-2.42091100
H	0.69370600	-2.81200700	-3.94949400
H	-1.21610100	-5.31030300	-1.02962700
H	1.56743200	-3.30778400	-1.66656800
C	-8.60783200	-2.05300900	-3.32800400
C	-7.33760100	-1.20388600	-3.21333200
C	-7.26959800	-0.47964300	-1.86554700
C	-6.09408800	-2.06931900	-3.42910500
H	-8.60247000	-2.84899800	-2.57349700
H	-7.36356600	-0.44404500	-4.00467200
H	-6.37073000	0.14342700	-1.79695000
H	-8.13993100	0.16685400	-1.70624500
H	-7.24117000	-1.20558500	-1.04505000
H	-6.04450100	-2.86758200	-2.67962100
H	-6.10821200	-2.53127400	-4.41950400
H	-5.17944100	-1.47074300	-3.37022900
C	-3.93587500	6.91725300	-2.37575600
C	-2.65036900	6.19694200	-1.95531300
C	-2.72499100	5.80757600	-0.47579600
C	-2.38934500	4.97620400	-2.84851600
H	-4.80165100	6.25786600	-2.24828100
H	-1.81114000	6.89535400	-2.08325000
H	-3.50852700	5.05845900	-0.31590800
H	-1.78618800	5.38966400	-0.10542000
H	-2.97209400	6.67238800	0.14800500
H	-3.18133200	4.22784800	-2.72739300
H	-2.36315900	5.25643700	-3.90639500
H	-1.43413000	4.49380500	-2.61533800
C	-8.45196300	6.41576300	-0.27662300
C	-7.63759700	5.12629900	-0.42781600
C	-7.40730300	4.81521100	-1.91599100
C	-8.31600700	3.96709300	0.30892100
C	-6.45173600	3.65158200	-2.18605500
H	-9.42612300	6.31682700	-0.77112200
H	-6.65376600	5.28687600	0.03794100
H	-7.01921800	5.71657300	-2.40771400
H	-8.37770100	4.60917200	-2.38927900
H	-9.30895300	3.76722300	-0.11335600
H	-7.73310000	3.04547200	0.24544900
H	-8.43453700	4.19780400	1.37101800
H	-6.26749500	3.53584600	-3.25821900

H	-5.48254000	3.82172600	-1.70181300	C	-5.41297100	-1.15383400	4.94527600
H	-6.84536200	2.70026000	-1.81575500	C	-4.19772700	-0.26940800	5.14713700
C	-6.86918700	7.29734300	3.04924800	C	-2.98690200	-0.76226500	5.65266500
C	-5.42064600	6.96461300	3.41347700	C	-4.27445500	1.09057600	4.81518800
C	-4.79552700	5.87612500	2.54176200	C	-1.89410400	0.08883300	5.83158100
S	-5.46776900	4.20994500	2.93849700	C	-3.18148200	1.93973700	4.98033700
C	-4.47167400	3.22486800	1.76966500	C	-1.98368500	1.44012100	5.49690600
H	-7.51534000	6.41836900	3.13804900	H	-4.58487500	-3.12123100	5.47503500
H	-5.34939200	6.66996800	4.46764600	H	-6.30697900	-0.57146300	5.19946100
H	-4.79889800	7.86051800	3.30284200	H	-5.50911500	-1.37513000	3.87427200
H	-3.71361100	5.84851000	2.69689200	H	-2.88716600	-1.81433200	5.89881000
H	-4.98457400	6.08617000	1.48405700	H	-5.20866300	1.49783800	4.43737100
H	-4.83538800	3.33537700	0.74453300	H	-0.96688700	-0.31023900	6.23440300
H	-3.42684100	3.53700300	1.82523800	H	-3.28462600	2.98911000	4.72006900
H	-4.54148300	2.17779600	2.06607200	H	-1.13202100	2.09840700	5.64167600
C	-8.61389500	-1.34196100	1.73388400	C	-1.69369800	-5.36798200	7.21805400
C	-8.35020700	0.02043800	2.38769900	C	-1.62234500	-4.95160100	5.74897800
C	-7.06467200	0.65893300	1.85142600	O	-2.44867800	-4.18514300	5.24107500
C	-9.54738800	0.95855700	2.19508800	H	-0.79546200	-5.88711200	7.55950100
H	-8.79462300	-1.22898400	0.65959900	N	-0.60611600	-5.50203200	5.04736800
H	-8.21590500	-0.14374800	3.46629100	C	-0.51710800	-5.51822100	3.59604300
H	-6.89746600	1.63696300	2.31144500	C	0.01533400	-6.91278300	3.23116400
H	-6.18742700	0.03483900	2.04928500	O	0.67207400	-7.55423000	4.05675400
H	-7.12538100	0.80875100	0.76773900	H	0.18746600	-4.76435700	3.22656800
H	-9.37941300	1.92112600	2.68797900	H	-1.49475500	-5.30230900	3.15824200
H	-9.71327400	1.15764400	1.12979800	H	-0.01925300	-6.21760700	5.46679100
H	-10.46649800	0.52303300	2.60163800	N	-0.28324400	-7.34809900	1.99874400
C	-7.99933500	-5.07196200	1.16670000	C	0.13057800	-8.65659500	1.53399800
C	-6.69108100	-4.95450600	0.43428900	H	-0.63906400	-9.05988700	0.87477500
O	-6.60325100	-4.58203200	-0.72757800	H	-0.90674100	-6.81636100	1.39860400
H	-7.88635600	-5.17092500	2.24941500	C	3.19760600	-1.20934500	8.56165600
N	-5.60997700	-5.37898700	1.16848600	C	2.85764700	-1.15658700	7.07001300
C	-4.27941700	-5.29785100	0.63131600	O	3.89318000	-1.78545500	6.30157200
C	-3.55052600	-6.62207100	0.52510000	H	4.14877300	-0.70537700	8.75725100
O	-2.34068500	-6.65124600	0.27453600	H	2.73021100	-0.10965700	6.75419600
H	-3.64581600	-4.62962900	1.21823100	H	1.89966300	-1.66729600	6.89680100
H	-4.34884400	-4.86102700	-0.36843500	H	3.52577600	-2.05649400	5.44536400
H	-5.71379800	-5.43915500	2.17089700	C	0.46180700	7.45456700	6.60201700
N	-4.27041400	-7.74245300	0.70445700	C	-0.26665900	7.08910900	5.30371400
C	-3.63971800	-9.04571500	0.62550500	C	-0.81956000	8.32728600	4.59794900
H	-3.04741100	-9.25747900	1.52211500	S	-1.73202400	8.00499400	3.04205700
H	-2.97561400	-9.07914300	-0.24020800	C	-0.37998500	7.37760300	1.98931900
H	-5.25751400	-7.64712800	0.89116100	H	0.85759000	6.56322600	7.09854700
C	-5.41949000	-2.46478500	5.73477900	H	-1.09104900	6.39737200	5.51051200

H	0.42064200	6.56053700	4.63261500	H	11.76181200	-1.17922000	-0.89476600
H	-0.01503200	9.04091200	4.38364000	H	11.19202900	-2.58647000	-1.81470500
H	-1.54051900	8.84148400	5.24215000	H	10.75054700	-0.94833000	-2.33572200
H	-0.74000300	7.39081200	0.96081800	H	8.55286900	-0.30981100	0.29094000
H	0.49099600	8.03545100	2.05965100	N	7.51151800	-0.19144700	-2.27446000
H	-0.10213700	6.35318700	2.23604400	C	7.22886200	0.83843900	-3.25449700
C	5.52532000	1.21509700	4.76463900	C	8.24920500	1.98645600	-3.27818000
C	6.11122900	0.30961000	3.71013700	O	8.06901900	2.92747800	-4.04908000
O	6.67935600	0.79056700	2.72067300	C	5.79388300	1.39605000	-3.08574500
H	5.30130000	0.69608000	5.69944600	C	4.82738000	0.99852100	-4.21463700
N	5.95411100	-1.01766100	3.92220700	C	5.29720900	1.52664900	-5.57595100
C	6.25035200	-2.03095000	2.93006000	C	4.56717700	-0.51283200	-4.25079800
C	5.00011600	-2.77937600	2.49807800	H	7.33476500	0.36172100	-4.23333600
O	3.88821100	-2.55899200	3.01609100	H	5.84558800	2.48503300	-3.03732800
H	6.69715900	-1.55323000	2.05871600	H	5.39199600	1.04933500	-2.12765500
H	5.43385700	-1.29581300	4.74704700	H	3.87607000	1.49194400	-3.98417700
N	5.21706000	-3.66720500	1.52171800	H	4.53526900	1.35492800	-6.34430400
C	4.13141200	-4.34827500	0.84214500	H	5.51910500	2.59633200	-5.53051300
C	4.29069000	-4.32943200	-0.68399500	H	6.21166600	1.02562600	-5.90990000
C	4.60647700	-2.94591800	-1.28067500	H	7.21321600	-0.02017000	-1.31120100
C	3.61691600	-1.84630300	-0.88495400	H	3.92770100	-0.78240500	-5.09978800
O	2.40761900	-1.97369100	-1.22135700	H	5.49972900	-1.07917400	-4.35531400
O	4.07244300	-0.84437900	-0.24655500	H	4.06377500	-0.84432200	-3.33888900
H	3.20915700	-3.83911700	1.13146500	N	9.32164800	1.88395200	-2.44770400
H	3.36451800	-4.72312900	-1.11321500	C	10.14222200	3.05905600	-2.19160600
H	5.09805000	-5.01223100	-0.97184900	C	9.79930400	3.68084100	-0.81680800
H	4.59724400	-3.02914200	-2.37262900	C	8.30532300	3.82879300	-0.62258800
H	5.60907000	-2.63557300	-0.98800900	C	7.56581700	4.71585400	-1.41624600
H	6.15955300	-3.80272900	1.15373200	C	7.61945800	3.01616600	0.28755400
C	8.51634700	-6.00725700	-1.51048600	C	6.17721400	4.76738900	-1.32027800
C	8.23805900	-4.62613100	-0.94732500	C	6.22799800	3.06698900	0.39171800
O	7.71076600	-4.48324600	0.16392600	C	5.50434100	3.93411000	-0.42417900
H	7.58586200	-6.58051600	-1.52294800	H	9.93386600	3.75977600	-3.00109100
H	8.94321200	-5.98546000	-2.51564000	H	10.30703000	4.65026300	-0.73482000
N	8.63296400	-3.58537100	-1.71358500	H	10.20204600	3.04047400	-0.02433900
C	8.43174700	-2.21416800	-1.31173200	H	8.07793200	5.33773700	-2.14397000
C	8.09375900	-1.37546900	-2.55376900	H	8.17274100	2.32401000	0.91550000
O	8.40124700	-1.77531400	-3.68141300	H	5.61641400	5.44228800	-1.96086500
C	9.68627300	-1.64256800	-0.58176800	H	5.72381500	2.42169100	1.10282900
C	10.92275200	-1.58659700	-1.46461400	H	4.42285800	3.95067800	-0.38804900
O	9.44182300	-0.32229900	-0.10542000	H	9.31953400	1.14387300	-1.74997100
H	7.61051300	-2.18145900	-0.59674100	C	1.53511200	3.85771800	0.04042400
H	9.86938000	-2.32085100	0.26568800	O	1.41931100	5.03324700	0.75219500
H	8.90136000	-3.70659600	-2.68349900	C	0.16043900	3.27199700	-0.25878300

C	0.32947800	1.99229300	-1.13032100	H	-0.00658000	-2.70203400	2.48915200
C	1.15286400	2.37147000	-2.33824300	H	-2.90773200	-1.10706200	-2.45570900
C	2.00807000	3.41544300	-2.28397500	H	-3.49640900	-2.54245800	3.31289500
O	2.22033800	4.18565900	-1.20788700	H	-5.40564000	-2.30097400	1.75645800
C	-0.72103200	3.04240400	0.93870000	H	-5.04048500	-1.72612400	-0.63806300
C	-0.40793500	3.11726000	2.23124600	H	-0.46305400	-0.92498000	-2.43934300
C	0.94481900	0.79467300	-0.37795700	H	0.39244400	-2.00372400	-0.15183200
C	-0.03380700	0.11750200	0.57628500	H	-1.17789300	0.05744500	3.06572200
C	0.99440600	1.59534900	-3.56758100	H	-8.44841500	-1.03453600	-8.75258300
O	0.35087100	0.55430900	-3.64473200	H	-7.24111500	-2.29011100	-8.44916700
O	1.60753200	2.13724800	-4.64076700	H	-9.51027000	-1.44945000	-3.18124000
C	1.49946700	1.38471500	-5.85882700	H	-8.67633200	-2.52942500	-4.31210800
O	3.25602000	0.04698300	2.17719400	H	-3.80715000	-6.35740000	-5.21072500
C	2.65936000	5.55378800	1.23389900	H	-4.80243300	-5.38692600	-6.31327400
H	-0.33933500	4.01079000	-0.89695000	H	-3.90178500	7.22251900	-3.42693200
H	-0.66820800	1.69808100	-1.47993300	H	-4.10671600	7.81220000	-1.76865600
H	2.18930900	3.14467100	0.56280600	H	-7.27065600	8.07932000	3.70096200
H	2.60574100	3.72943200	-3.13146400	H	-6.93476900	7.65438000	2.01668800
H	-1.73669700	2.75752600	0.66751700	H	-7.93589300	7.27006300	-0.72853800
H	-1.14423500	2.88078300	2.99285300	H	-8.63733900	6.65010400	0.77506700
H	0.58006200	3.40052200	2.57516400	H	-0.21162400	7.95707800	7.30486800
H	1.81026400	1.12486700	0.20088100	H	1.30263100	8.12963900	6.40818700
H	1.34795000	0.06822000	-1.08459100	H	3.28224500	-2.24572500	8.89937200
H	-1.03063500	0.55309000	0.61729900	H	2.41451800	-0.70985100	9.13983400
H	1.89765700	0.37756300	-5.72219000	H	-6.35143500	-3.00979200	5.54986000
H	0.45467900	1.32281400	-6.17355400	H	-5.35664100	-2.26925700	6.81073500
H	2.09084600	1.93229400	-6.59063000	H	9.20891600	-6.52217900	-0.83915800
H	3.27239900	5.94334400	0.41658800	H	11.20442400	2.79397000	-2.22825600
C	-0.32517000	-0.61603900	2.92539100	H	-4.40916800	-9.81224400	0.51973500
N	0.47388300	-0.16871100	1.80073100	H	4.59310300	1.61455700	4.35165100
C	-0.84094200	-2.06888000	2.81310000	H	6.20723500	2.04748900	4.94398900
N	-2.36676200	-1.32031100	-1.62419600	H	4.05215400	-5.38560500	1.19009000
C	-1.99234500	-2.15235800	1.84179500	H	6.97798200	-2.75265300	3.32276600
C	-3.31413600	-2.31707400	2.26920000	H	2.40768500	6.36091400	1.92243700
C	-4.39576200	-2.17028800	1.38330000	H	3.22884600	4.78163300	1.76657600
C	-4.20632600	-1.85657300	0.03901400	H	-9.48847100	-1.83374900	2.17364900
C	-2.88413700	-1.72275100	-0.38145200	H	-7.75333700	-2.00926800	1.85562300
C	-1.03222600	-1.24717800	-1.58150800	H	-8.61285500	-4.19631400	0.95024800
C	-0.56786500	-1.51234200	-0.25922500	H	-8.53599100	-5.94989300	0.79317100
C	-1.78309100	-1.89487600	0.48188800	H	-1.85937700	-4.48029400	7.83222400
H	0.30597200	-0.52562200	3.81365100	H	-2.55703400	-6.02691700	7.34956700
H	1.49794500	-0.21412200	1.90464100	H	1.07219700	-8.60919000	0.97446500
H	3.51860900	-0.17606100	1.25236700	H	0.27415900	-9.31196700	2.39396900
H	-1.16760400	-2.41567200	3.79656100	H	3.47153900	-0.77792500	2.65423900

O	6.58933000	-0.31584600	0.31725400
H	6.54856000	0.21751900	1.14148100
H	5.64908400	-0.56226400	0.08301900

Int6 (14.7)

C	-7.38682800	-1.21035400	-8.55906300
C	-6.97722500	-0.38673900	-7.33028400
C	-5.53606400	-0.53627000	-6.93384100
C	-4.58377300	-1.33849900	-7.51231800
C	-4.88044200	0.14410000	-5.84064200
C	-3.53034200	-0.30615800	-5.80489500
C	-5.30405200	1.08739700	-4.88920600
N	-3.37558400	-1.19795500	-6.84537600
C	-2.61695000	0.13853100	-4.84458600
C	-4.40333000	1.53040300	-3.92702400
C	-3.07441200	1.06018100	-3.90593800
H	-6.79915500	-0.92450600	-9.43697100
H	-7.61351100	-0.66432900	-6.47770300
H	-7.18998700	0.67481300	-7.51746300
H	-4.66359200	-2.00707100	-8.35613400
H	-6.32209800	1.46421100	-4.90487200
H	-2.52747300	-1.68963400	-7.07099100
H	-1.58527400	-0.19059000	-4.82598800
H	-4.71813400	2.26009100	-3.18989900
H	-2.37613600	1.44224300	-3.16890000
C	-3.85330800	-5.41660200	-5.76832200
C	-3.77308800	-4.17421000	-4.85663100
C	-2.53598100	-4.08647700	-3.98415400
C	-1.42739100	-3.29780800	-4.31508000
C	-2.46411200	-4.80552700	-2.77989600
C	-0.29126400	-3.22482600	-3.49911300
C	-1.34929700	-4.74857200	-1.95545700
C	-0.24032600	-3.96243100	-2.30517500
O	0.81254800	-3.98153600	-1.45854700
H	-3.01237700	-5.44703300	-6.46934700
H	-4.65470400	-4.17191600	-4.20772900
H	-3.84936600	-3.27347500	-5.47197300
H	-1.44297400	-2.72034700	-5.23582600
H	-3.30773400	-5.42615100	-2.48468200
H	0.55058800	-2.59851700	-3.78106600
H	-1.32330700	-5.30225200	-1.02702600
H	1.43103000	-3.20177500	-1.53989600
C	-8.60786900	-2.05302200	-3.32798400
C	-7.37086600	-1.18962300	-3.07139800
C	-7.36118800	-0.67051200	-1.63156100
C	-6.08794800	-1.96907700	-3.37166000
H	-8.59233000	-2.93971500	-2.68181600
H	-7.40784900	-0.32525600	-3.74747500
H	-6.48409900	-0.04429600	-1.43870900
H	-8.25058200	-0.07041300	-1.41218200
H	-7.34576100	-1.51132600	-0.92884600
H	-5.99761800	-2.83364400	-2.70439900
H	-6.07450600	-2.32594800	-4.40501200
H	-5.20644000	-1.33145500	-3.24539500
C	-3.93587000	6.91727500	-2.37573000
C	-3.16996000	5.96803800	-1.44257000
C	-3.66303300	6.10781200	0.00031500
C	-3.29457800	4.51249000	-1.91236100
H	-5.00380700	6.66702100	-2.38014000
H	-2.10615300	6.24703700	-1.46869900
H	-4.71246100	5.79982800	0.08269100
H	-3.07476500	5.48488700	0.67829200
H	-3.59651100	7.14172900	0.35437000
H	-4.34510900	4.20151600	-1.90519900
H	-2.91380800	4.37742800	-2.92978600
H	-2.74558400	3.82779600	-1.25302400
C	-8.45204400	6.41578800	-0.27666100
C	-7.87778500	5.03100200	0.06332100
C	-7.49547300	4.28866300	-1.22585800
C	-8.85926000	4.23344000	0.93225400
C	-6.84581100	2.92259200	-1.00222900
H	-9.37297300	6.31937900	-0.86347100
H	-6.96169000	5.17538900	0.64975000
H	-6.81055300	4.92549500	-1.80246100
H	-8.39236900	4.16461700	-1.84921800
H	-9.78142900	4.01839700	0.37717900
H	-8.43380900	3.28378100	1.27451500
H	-9.13371900	4.79979300	1.82792300
H	-6.54080300	2.46490300	-1.94824100
H	-5.95982600	3.00190300	-0.36250100
H	-7.53591300	2.22484200	-0.52235900
C	-6.86912500	7.29729800	3.04924300
C	-5.82237600	6.77744200	4.04272100
C	-4.90022100	5.68638200	3.49572500
S	-5.77463300	4.08875900	3.22008600
C	-4.44391700	3.19117500	2.35125700
H	-7.56540300	6.50736400	2.75801500
H	-6.31569400	6.41609100	4.95373900

H	-5.17379700	7.60685800	4.34984200	H	-5.33933100	-1.17899500	4.00293900
H	-4.07009400	5.51210700	4.18616500	H	-2.82923200	-2.03024700	6.02131000
H	-4.46816000	6.00438000	2.54698500	H	-4.86218900	1.58826400	4.88877600
H	-4.26566700	3.62241400	1.36448800	H	-0.80214600	-0.72562300	6.52600400
H	-3.52035400	3.21033600	2.93301000	H	-2.82691900	2.88555800	5.36467200
H	-4.76144700	2.15461900	2.23771500	H	-0.77128500	1.73758400	6.18724900
C	-8.61380800	-1.34195500	1.73382000	C	-1.69369400	-5.36798100	7.21803700
C	-8.21607400	-0.30374700	2.78962300	C	-1.64439600	-4.99228000	5.73973100
C	-7.03607800	0.54842600	2.31389300	O	-2.50686500	-4.27360300	5.22083000
C	-9.40427000	0.58788500	3.16890200	H	-0.87150700	-6.01686300	7.52706000
H	-8.97222200	-0.85276300	0.82022400	N	-0.59896700	-5.49732800	5.04669200
H	-7.90030400	-0.84815100	3.69086300	C	-0.51711900	-5.51824000	3.59605200
H	-6.74435500	1.27151800	3.08145100	C	0.01518100	-6.91185100	3.22517500
H	-6.16468100	-0.06825000	2.06799500	O	0.65735600	-7.56874000	4.04935300
H	-7.30261700	1.11567600	1.41433500	H	0.18038100	-4.75906300	3.22087900
H	-9.12777200	1.30607700	3.94759100	H	-1.49935900	-5.30724800	3.16522700
H	-9.74982100	1.16007900	2.29955600	H	-0.00117000	-6.20358800	5.46668700
H	-10.24921500	-0.00398400	3.53711300	N	-0.26237000	-7.33469800	1.98396000
C	-7.99933400	-5.07196200	1.16670000	C	0.13058100	-8.65659200	1.53399700
C	-6.68926500	-4.90742200	0.44498500	H	-0.31252900	-8.82402500	0.55203800
O	-6.59359100	-4.46673400	-0.69238500	H	-0.90450100	-6.81598000	1.39211200
H	-7.90230900	-4.96521900	2.25101000	C	3.19760600	-1.20934500	8.56165600
N	-5.61454000	-5.38056800	1.15786200	C	2.83562500	-1.18840800	7.07438900
C	-4.27941700	-5.29785300	0.63131700	O	3.88952600	-1.77515500	6.29844300
C	-3.55678700	-6.62545300	0.52951900	H	4.12483100	-0.65669100	8.73972200
O	-2.34315100	-6.66161400	0.30002500	H	2.65290600	-0.15226500	6.75007900
H	-3.64644600	-4.63578900	1.22616800	H	1.90109300	-1.74697400	6.91972500
H	-4.34056200	-4.85528300	-0.36623700	H	3.52962600	-2.05024900	5.44014500
H	-5.72409400	-5.49926500	2.15459700	C	0.46180300	7.45457000	6.60200800
N	-4.27931400	-7.74545000	0.70038200	C	-0.39132700	7.05074700	5.39397500
C	-3.63971800	-9.04571500	0.62550700	C	-0.92602800	8.27040800	4.64249600
H	-2.88469100	-9.14888000	1.40961800	S	-1.98779500	7.89699600	3.19604400
H	-3.14573500	-9.17774900	-0.34108900	C	-0.75686600	7.12999800	2.08703400
H	-5.27514400	-7.65561700	0.83439500	H	0.84065300	6.57457700	7.13105900
C	-5.41951400	-2.46478200	5.73483100	H	-1.23659400	6.43114500	5.71499000
C	-5.27306100	-1.08025900	5.09360400	H	0.20465300	6.43237700	4.71247300
C	-4.00205800	-0.31950700	5.42008100	H	-0.10038300	8.91013800	4.30867600
C	-2.84280900	-0.95407600	5.88539900	H	-1.55473700	8.87747000	5.30242800
C	-3.97098300	1.07070000	5.23430200	H	-1.21176100	7.06295700	1.09819600
C	-1.68994400	-0.21481200	6.16203100	H	0.13289400	7.76100800	2.01266900
C	-2.81819900	1.80764800	5.50209400	H	-0.47644200	6.12560500	2.40275300
C	-1.66916500	1.16630200	5.97102600	C	5.52532000	1.21509700	4.76464100
H	-4.62762700	-3.15600300	5.43537700	C	6.10813100	0.31117200	3.70773500
H	-6.12816900	-0.45530100	5.37763900	O	6.67066100	0.79402800	2.71565700

H	5.31206400	0.69680600	5.70243000	C	4.80604100	1.05572800	-4.16240200
N	5.95463900	-1.01609700	3.92083100	C	5.25399000	1.58296000	-5.53133700
C	6.25034400	-2.03094900	2.93005700	C	4.51505100	-0.44965200	-4.20076500
C	4.99810100	-2.77440700	2.49470400	H	7.30922600	0.36443400	-4.23678100
O	3.88958400	-2.56285900	3.02331300	H	5.88079100	2.51131700	-2.99821400
H	6.70123900	-1.55442100	2.06007600	H	5.41897700	1.07947100	-2.08906800
H	5.43555000	-1.29426000	4.74624200	H	3.86973700	1.56640600	-3.90494700
N	5.21437400	-3.65252900	1.50882300	H	4.47075500	1.43155900	-6.28260800
C	4.13141400	-4.34827800	0.84214700	H	5.49908900	2.64742600	-5.48480500
C	4.22615200	-4.27378900	-0.68881400	H	6.14997000	1.06572700	-5.88968400
C	4.51121800	-2.87212400	-1.25728600	H	7.20184500	-0.03030300	-1.31669900
C	3.53724100	-1.77906200	-0.80327300	H	3.84541100	-0.70208000	-5.03150300
O	2.31361700	-1.89972200	-1.08580900	H	5.43304200	-1.03306400	-4.33591100
O	4.02425900	-0.78442600	-0.17688000	H	4.03475500	-0.77640500	-3.27473600
H	3.20414400	-3.88116400	1.18226700	N	9.32631000	1.87984600	-2.44845000
H	3.28333200	-4.65760000	-1.09097800	C	10.14222300	3.05905700	-2.19160600
H	5.02242700	-4.94236300	-1.03550600	C	9.79121100	3.67972500	-0.81923300
H	4.46074600	-2.92958900	-2.34980800	C	8.29724000	3.83555300	-0.62735000
H	5.52428200	-2.56436200	-0.99760200	C	7.56288700	4.73040700	-1.41701700
H	6.15606400	-3.78312200	1.13707200	C	7.60702500	3.03372700	0.28923200
C	8.51634800	-6.00725700	-1.51048600	C	6.17659100	4.80783700	-1.30309400
C	8.22966200	-4.62544100	-0.95388000	C	6.21779600	3.10926200	0.41003800
O	7.68392400	-4.48223300	0.14835300	C	5.49886700	3.99144100	-0.39453800
H	7.58964900	-6.58642400	-1.51828500	H	9.93431500	3.75700700	-3.00362700
H	8.94192000	-5.98771100	-2.51629900	H	10.30192700	4.64727300	-0.73297700
N	8.63963700	-3.58478000	-1.71238700	H	10.18846500	3.03733400	-0.02553000
C	8.43174400	-2.21416900	-1.31173100	H	8.07729900	5.34387000	-2.15041900
C	8.10713700	-1.37276400	-2.55577900	H	8.15566500	2.33511500	0.91397800
O	8.43375700	-1.76489000	-3.68035200	H	5.62117200	5.49407400	-1.93631300
C	9.67450800	-1.64112600	-0.56355200	H	5.71249100	2.47193600	1.12780800
C	10.92258300	-1.57959600	-1.42948900	H	4.41794500	4.03538500	-0.33667200
O	9.41966300	-0.32242600	-0.08811800	H	9.31621700	1.14600600	-1.74416400
H	7.59941800	-2.18451100	-0.60872800	C	1.45687500	3.99703700	0.21120800
H	9.84809700	-2.32052100	0.28503700	O	1.37412600	5.16249000	0.94094500
H	8.92462600	-3.70520300	-2.67764300	C	0.06531700	3.43467600	-0.06290100
H	11.75244900	-1.17029500	-0.84767700	C	0.20464900	2.15844000	-0.95120000
H	11.19994700	-2.57805200	-1.77730800	C	1.02712600	2.52898300	-2.16366200
H	10.75985800	-0.94073200	-2.30193800	C	1.89692100	3.56058600	-2.11674800
H	8.52754700	-0.31426900	0.30082200	O	2.12406600	4.33011300	-1.04430400
N	7.50810500	-0.19636400	-2.27864000	C	-0.77696100	3.19871800	1.16372400
C	7.22886000	0.83844000	-3.25449800	C	-0.38092100	3.15091500	2.43480100
C	8.26144700	1.97591400	-3.28966800	C	0.81783900	0.94361700	-0.21998100
O	8.09055700	2.90971600	-4.07124200	C	-0.15026400	0.23661000	0.72660200
C	5.80709300	1.42398600	-3.05346900	C	0.86290900	1.75095000	-3.39070800

O	0.17266200	0.74076600	-3.47798000
O	1.53156500	2.25022300	-4.45090000
C	1.41554400	1.49500200	-5.66690400
O	3.18549200	0.05965400	2.24911800
C	2.62907400	5.65313700	1.41193300
H	-0.44645300	4.19095700	-0.67276300
H	-0.79953800	1.88100300	-1.29518000
H	2.11072800	3.26596900	0.70864900
H	2.49479700	3.86387300	-2.96793200
H	-1.82619600	3.01531300	0.93683200
H	-1.08200800	2.91852400	3.23020300
H	0.64827600	3.31958500	2.73086800
H	1.68406800	1.26370600	0.36350900
H	1.22106300	0.23348100	-0.94349300
H	-1.14342700	0.67881600	0.79851400
H	1.78136500	0.47707400	-5.51937600
H	0.37386900	1.46292900	-5.99543200
H	2.03410100	2.01976700	-6.39282800
H	3.24273700	6.03460200	0.59107400
C	-0.37983100	-0.57559800	3.06360200
N	0.38471000	-0.07304200	1.93824000
C	-0.84968300	-2.04145500	2.92020300
N	-2.51960800	-1.20083200	-1.45034700
C	-2.02122400	-2.13352700	1.97495700
C	-3.32321700	-2.37573200	2.42769100
C	-4.43326500	-2.23869400	1.57585700
C	-4.29231200	-1.86520200	0.24016500
C	-2.98875500	-1.65922300	-0.20636400
C	-1.18945900	-1.07662700	-1.43977700
C	-0.59698157	-1.11517759	-0.00332521
C	-1.85987700	-1.81120200	0.62310000
H	0.26348400	-0.48852700	3.94300600
H	1.41083900	-0.14250700	2.00975200
H	3.44612400	-0.13323500	1.31585100
H	-1.14243100	-2.42554700	3.90019500
H	-0.00234200	-2.63736300	2.55866200
H	-3.09130700	-0.99636000	-2.26359000
H	-3.46291600	-2.66370900	3.46229600
H	-5.42825800	-2.43172500	1.96352900
H	-5.14964300	-1.74868900	-0.40934600
H	-0.65397300	-0.71400700	-2.30361500
H	0.37490243	-1.59426359	0.05703179
H	-1.25128400	0.06572900	3.23908500
H	-8.44299500	-1.05700300	-8.79477000
H	-7.23303600	-2.27965600	-8.38310900
H	-9.53254400	-1.50090700	-3.12362900
H	-8.64449000	-2.39844900	-4.36719600
H	-3.82293000	-6.33832500	-5.17892800
H	-4.78317200	-5.41043300	-6.34638800
H	-3.57257300	6.83806100	-3.40754700
H	-3.83495800	7.95760300	-2.05771500
H	-7.44973000	8.11474400	3.49275900
H	-6.39939900	7.67724200	2.13914100
H	-7.74237200	7.00721100	-0.86108000
H	-8.69140300	6.97485100	0.63079500
H	-0.11972800	8.04879100	7.31549300
H	1.32395300	8.05631900	6.29479100
H	3.33829200	-2.23690000	8.90756500
H	2.39796800	-0.74398600	9.14566200
H	-6.38195100	-2.90548000	5.45150300
H	-5.39443100	-2.39429600	6.82673900
H	9.21371700	-6.51427200	-0.83794700
H	11.20566200	2.79816100	-2.22339400
H	-4.39489400	-9.82238600	0.75265300
H	4.58630400	1.60549200	4.35828100
H	6.20282300	2.05272600	4.93586900
H	4.10146400	-5.39863600	1.15609800
H	6.97332700	-2.75524700	3.32606300
H	2.40121900	6.46115500	2.10790200
H	3.18717900	4.86538800	1.93464700
H	-9.41168900	-1.99536600	2.10093000
H	-7.76102200	-1.96931600	1.45455900
H	-8.71094500	-4.33392400	0.79664300
H	-8.40383800	-6.06828300	0.95788800
H	-1.67705800	-4.45428500	7.81857000
H	-2.64448200	-5.87004600	7.41387000
H	1.21885700	-8.74087900	1.45556400
H	-0.21303000	-9.42702200	2.23211300
H	3.41775100	-0.77653300	2.69730200
O	6.56587200	-0.30605900	0.31176800
H	6.54352400	0.22358700	1.13943300
H	5.61598200	-0.53550200	0.09784500

TS4 (21.2)

C	7.77667000	-1.43704300	8.40026100
C	7.23842300	-0.78080100	7.12109800
C	5.78202300	-1.03829700	6.84505600
C	5.01773200	-2.10632900	7.24886000

C	4.90376400	-0.18407100	6.08199100	H	5.44033300	-1.47096000	3.64751100
C	3.61231100	-0.78593300	6.08013700	C	3.76428100	6.87954900	2.83552300
C	5.08416900	1.02992300	5.39806200	C	2.43941300	6.10766000	2.74746500
N	3.71658400	-1.96248000	6.78986100	C	1.81685900	6.22061100	1.35002600
C	2.50636900	-0.17801700	5.47436600	C	2.63325500	4.63786000	3.13668300
C	3.99575200	1.62794900	4.77657300	H	4.49286800	6.47428100	2.12258400
C	2.71105400	1.04489500	4.83428400	H	1.73907700	6.55786500	3.46599500
H	7.21157300	-1.10643500	9.27701800	H	2.46746700	5.76472000	0.59661300
H	7.84067800	-1.11074100	6.26326500	H	0.84906000	5.70733600	1.30583300
H	7.39085300	0.30374100	7.18494400	H	1.66368900	7.26491900	1.05851500
H	5.29034800	-2.96926900	7.83772600	H	3.35683300	4.15645100	2.46722700
H	6.06320400	1.49764900	5.35836100	H	3.01679600	4.54905000	4.15863700
H	2.97258200	-2.62391400	6.93877000	H	1.69782200	4.07353100	3.07406600
H	1.51889100	-0.62783400	5.52636700	C	8.21680800	6.69732300	0.55623100
H	4.12532200	2.56708000	4.25248500	C	7.55909900	5.31265900	0.51733200
H	1.86557600	1.57639100	4.40334100	C	7.25170400	4.81963400	1.94239500
C	4.32003300	-5.62404100	5.48694900	C	8.42729800	4.32321800	-0.26692600
C	4.33292900	-4.61560800	4.33058700	C	6.45845800	3.51226000	2.00741800
C	3.08564800	-4.64343100	3.47737700	H	9.15458500	6.66616700	1.12491300
C	2.22760000	-3.54358700	3.39490600	H	6.60601200	5.39960800	-0.02077900
C	2.75162800	-5.78167900	2.72780600	H	6.69359800	5.60280600	2.47188600
C	1.09132900	-3.55915600	2.58290900	H	8.19845100	4.70072900	2.48860100
C	1.62319400	-5.81763100	1.92006400	H	9.38796400	4.15867100	0.23782900
C	0.79600400	-4.69224000	1.81703900	H	7.93443600	3.35635600	-0.38494800
O	-0.25869200	-4.75929600	0.96062600	H	8.63603700	4.70237500	-1.27225500
H	3.47671300	-5.43533000	6.16128400	H	6.21508000	3.24554400	3.04111600
H	5.21059300	-4.80957100	3.70183600	H	5.51489200	3.59614900	1.45692300
H	4.46788700	-3.60979900	4.73535200	H	7.01247400	2.67415200	1.57687600
H	2.46153400	-2.64573900	3.96199000	C	6.47640000	7.70101300	-2.65464700
H	3.39648100	-6.65663800	2.77102500	C	4.95614600	7.51173900	-2.65257100
H	0.44140700	-2.69251700	2.52966000	C	4.44888000	6.43167800	-1.69438000
H	1.39161900	-6.68899500	1.32250500	S	4.90288900	4.73934500	-2.26024200
H	-0.71505800	-3.89188200	0.92021200	C	3.75077000	3.76763200	-1.23060800
C	8.83723900	-1.91969000	3.08890400	H	6.99460100	6.77429300	-2.92197600
C	7.53649300	-1.11399400	3.15956800	H	4.60240600	7.28151900	-3.66463100
C	7.28249600	-0.37126300	1.84507000	H	4.47325800	8.45357600	-2.36336700
C	6.36545100	-2.03593300	3.50134400	H	3.35943800	6.47582200	-1.63631100
H	8.75971500	-2.68984100	2.31203300	H	4.85097400	6.59255000	-0.68709600
H	7.63225500	-0.36700800	3.96081300	H	3.98714300	3.86828900	-0.16843300
H	6.36911700	0.23063600	1.89289600	H	2.71917400	4.08399800	-1.40883300
H	8.11255600	0.30041600	1.59953600	H	3.85769100	2.72047400	-1.52026500
H	7.17408300	-1.08331600	1.01936700	C	8.62671700	-0.91438800	-1.91946000
H	6.20766800	-2.76713900	2.70104600	C	7.91781600	0.32259300	-2.49139200
H	6.55364600	-2.58359900	4.42890900	C	6.83056200	0.83364500	-1.53750600

C	8.92641600	1.43160200	-2.81258200	H	0.82133100	-5.49893200	-7.72521500
H	9.14823200	-0.66632600	-0.98779000	N	0.67269000	-5.21852600	-5.19431000
H	7.43121300	0.02629400	-3.43246600	C	0.64677500	-5.32602600	-3.74603700
H	6.35166000	1.73671800	-1.93098300	C	0.17686900	-6.76471300	-3.44578400
H	6.05171200	0.08377800	-1.36454800	O	-0.36506400	-7.44089000	-4.32493300
H	7.26176600	1.09133200	-0.56495800	H	-0.05211700	-4.61097900	-3.29727900
H	8.42942700	2.30595400	-3.24488700	H	1.63884100	-5.12439400	-3.33430000
H	9.44125500	1.76114900	-1.90240300	H	0.11453500	-5.94561500	-5.63342100
H	9.68743900	1.08765700	-3.52143200	N	0.40880300	-7.20870800	-2.20162600
C	8.18865100	-4.69659700	-1.55348900	C	0.20631100	-8.60438800	-1.85395600
C	6.89770300	-4.58962300	-0.78927200	H	0.75270300	-8.80550400	-0.93194900
O	6.82003500	-4.15973500	0.35490500	H	0.99841500	-6.66262700	-1.58256200
H	8.05257400	-4.58043400	-2.63243500	C	-3.42522500	-0.90188800	-8.31447600
N	5.82335800	-5.09577800	-1.47473600	C	-3.18827100	-0.15117200	-7.00777500
C	4.50387800	-5.11443600	-0.90502900	O	-3.68439100	-0.90686300	-5.91039100
C	3.83212700	-6.47080700	-0.91333400	H	-4.49419500	-1.08510800	-8.46206800
O	2.61453600	-6.55992100	-0.75625800	H	-3.67519200	0.83777100	-7.05804800
H	3.82003000	-4.42807500	-1.40477100	H	-2.10951800	0.03541900	-6.88470100
H	4.58112000	-4.77380900	0.13177300	H	-3.31208000	-0.51373400	-5.08479200
H	5.91572500	-5.23540300	-2.47032900	C	-0.98108900	7.74449800	-5.93653300
N	4.61167200	-7.55587100	-1.09076700	C	-0.81487100	7.11904300	-4.54486300
C	4.02135300	-8.88064900	-1.10147200	C	0.43627700	7.64697600	-3.84180500
H	3.20673600	-8.92095700	-1.82801700	S	0.75152700	6.98169100	-2.16252500
H	3.61345600	-9.14347300	-0.11983900	C	-0.71216500	7.60241400	-1.26549200
H	5.61072400	-7.42754600	-1.14179100	H	-1.88027600	7.36708700	-6.43337500
C	5.33495200	-1.94202500	-5.86618700	H	-0.76108600	6.02734500	-4.60879500
C	4.62892300	-1.59075800	-4.54997800	H	-1.69690500	7.34213600	-3.93523100
C	3.72208100	-0.38993800	-4.69275300	H	0.40813300	8.74107100	-3.77411500
C	2.48495300	-0.51235500	-5.34161200	H	1.33465200	7.38367300	-4.41048100
C	4.11975000	0.87501200	-4.24417800	H	-0.53612600	7.40582400	-0.20620600
C	1.67629300	0.60593600	-5.54538700	H	-0.82228500	8.68055300	-1.41005500
C	3.31356000	1.99699500	-4.44496000	H	-1.61481600	7.07566700	-1.57530200
C	2.08901100	1.86474400	-5.10098000	C	-5.70955300	1.19360100	-4.30115700
H	4.59805400	-2.22683600	-6.62153900	C	-6.13729300	0.18957100	-3.26083200
H	5.37064400	-1.39623100	-3.76611800	O	-6.61300100	0.55454700	-2.17287000
H	4.03942300	-2.46156100	-4.25331100	H	-5.35749300	0.72212700	-5.21802000
H	2.16873200	-1.49436100	-5.68060200	N	-5.99124700	-1.11600400	-3.59009000
H	5.07676500	0.98330700	-3.74286700	C	-6.23054800	-2.18205200	-2.64004400
H	0.72052800	0.49392200	-6.05090200	C	-4.93304800	-2.72288700	-2.05284200
H	3.64842100	2.97345900	-4.10737400	O	-3.83124400	-2.25323300	-2.36557600
H	1.46493900	2.73771800	-5.26533800	H	-6.85671600	-1.79574000	-1.83656200
C	1.68094900	-4.91405500	-7.39115200	H	-5.46896000	-1.32637500	-4.43598900
C	1.66362000	-4.61722100	-5.89232400	N	-5.08889200	-3.77381300	-1.23271800
O	2.50643800	-3.88433400	-5.36528100	C	-3.94223100	-4.52300800	-0.76831300

C	-3.71249600	-4.49009200	0.74835600	H	-5.41896100	0.59200300	6.07459200
C	-3.73602300	-3.08772900	1.37364300	H	-6.90743500	-0.59163700	1.80309100
C	-2.80353100	-2.04187700	0.77864300	H	-3.08471700	-0.60492700	4.37131100
O	-1.52349100	-2.34091800	0.75948800	H	-4.73571700	-1.22342400	4.23379900
O	-3.24116800	-0.95499100	0.38676700	H	-3.88849900	-0.64200100	2.79105800
H	-3.07243800	-4.10832600	-1.28046500	N	-9.24182100	1.27649200	2.99914800
H	-2.75787300	-4.98531300	0.95239900	C	-10.13828000	2.42610500	2.90767200
H	-4.49114000	-5.07826900	1.24814200	C	-10.06547900	3.04837900	1.50502500
H	-3.47458900	-3.17376400	2.43482300	C	-8.65347900	3.42333700	1.11881000
H	-4.74454500	-2.67532700	1.31958600	C	-7.98797400	4.45776900	1.78985200
H	-6.01745900	-4.07906100	-0.94364500	C	-7.96917900	2.72253400	0.12028200
C	-8.16327100	-6.50541100	1.63075400	C	-6.67704200	4.79250900	1.45895200
C	-7.98680900	-5.07820900	1.15339900	C	-6.65713300	3.05777800	-0.21863700
O	-7.46748300	-4.83866800	0.05590400	C	-6.01012800	4.09635800	0.44840500
H	-7.18276300	-6.98751700	1.66511900	H	-9.81741900	3.12879300	3.67919800
H	-8.63645900	-6.57923300	2.61259400	H	-10.71927500	3.92910700	1.48410400
N	-8.44003600	-4.10107000	1.97381100	H	-10.46738000	2.33757500	0.77402000
C	-8.24397300	-2.70701700	1.65611400	H	-8.50242300	5.00242800	2.57719300
C	-7.98151200	-1.90680400	2.94050400	H	-8.46471900	1.91121700	-0.40543200
O	-8.41913700	-2.29460300	4.02534600	H	-6.17751800	5.60177300	1.98453200
C	-9.44332400	-2.10235900	0.86608700	H	-6.16328700	2.50035400	-1.00670200
C	-10.73600500	-2.06648300	1.66363200	H	-5.00081600	4.37482300	0.17497900
O	-9.16029600	-0.76011100	0.46432300	H	-9.14344800	0.67107800	2.18893400
H	-7.37307300	-2.63625600	0.99787500	C	-2.44376800	4.20605100	-1.41547000
H	-9.57052800	-2.74014700	-0.02085100	O	-2.59402200	5.00457900	-2.52840800
H	-8.74497900	-4.28917700	2.92175300	C	-0.99838900	3.75208300	-1.22963500
H	-11.53606300	-1.64455400	1.05019000	C	-0.92543800	2.86086100	0.05371000
H	-11.02449800	-3.07610500	1.96716700	C	-1.60943100	3.60338200	1.18354300
H	-10.62146300	-1.45438500	2.56197200	C	-2.47972200	4.60691700	0.94094500
H	-8.29052900	-0.73495600	0.03698000	O	-2.86176000	5.01661500	-0.27447600
N	-7.30203500	-0.75643500	2.73144000	C	-0.40682000	3.09303800	-2.44879600
C	-7.09775600	0.27688800	3.73519900	C	-1.06861600	2.59293100	-3.49097300
C	-8.29226400	1.22071600	3.96808400	C	-1.51112300	1.43643700	-0.15572300
O	-8.27983400	1.95268400	4.95478900	C	-0.48163000	0.44552100	-0.74088600
C	-5.90414300	1.18796600	3.32149300	C	-1.26645200	3.29004100	2.57340300
C	-4.58952900	0.94896800	4.08300400	O	-0.30967200	2.60287600	2.91832000
C	-4.72706100	1.27700000	5.57425300	O	-2.11023600	3.83667300	3.47750700
C	-4.04243900	-0.46312700	3.85690000	C	-1.78655300	3.59091600	4.85524900
H	-6.91739200	-0.20484900	4.69870000	O	-3.11088300	0.15742200	-3.47896300
H	-6.19555100	2.22986000	3.47134500	C	-3.95414900	5.20737800	-2.91104900
H	-5.73286300	1.07026900	2.24634600	H	-0.41387500	4.65892700	-1.03526800
H	-3.86850100	1.65910100	3.65277000	H	0.13141500	2.76045800	0.32885500
H	-3.75989400	1.19644100	6.08349300	H	-3.13839100	3.35324100	-1.44430600
H	-5.11645500	2.28871900	5.72242900	H	-2.94174900	5.18484400	1.73222700

H	0.67980300	3.01771700	-2.43722300
H	-0.53696100	2.10122600	-4.29677000
H	-2.14774300	2.62269400	-3.57681400
H	-2.36236400	1.47453600	-0.83924900
H	-1.91978900	1.05757300	0.78202300
H	0.31188400	1.04034100	-1.20228900
H	-1.76292900	2.51800800	5.05469100
H	-0.81535200	4.02447000	5.10605900
H	-2.58183400	4.06419300	5.42883600
H	-4.51495300	5.73659500	-2.13431100
C	-0.03826100	-0.88020500	-2.79473300
N	-1.01798700	-0.35982200	-1.83514900
C	0.88895900	-2.01679400	-2.33481900
N	1.74264900	-0.25764600	2.02406300
C	1.88940800	-1.67907300	-1.25904100
C	3.23285500	-2.04527200	-1.35686500
C	4.14037300	-1.88308200	-0.29442600
C	3.73594000	-1.34654300	0.92202000
C	2.41151300	-0.92559900	0.99286900
C	0.50844400	0.09165400	1.63557900
C	0.23322800	-0.40910600	0.33463000
C	1.49644100	-1.05715900	-0.06654300
H	-0.60863900	-1.24010600	-3.65800000
H	-1.62316400	-1.09829700	-1.48192900
H	-2.27353600	0.23076600	-2.95429000
H	1.41559200	-2.40560300	-3.21241900
H	0.25999000	-2.83493400	-1.95349600
H	2.14600800	-0.04762200	2.93335700
H	3.58066000	-2.48244900	-2.28454300
H	5.16670300	-2.21454300	-0.40499500
H	4.41614600	-1.25192500	1.75705600
H	-0.11352700	0.69371800	2.27907700
H	-0.72999500	-1.39631900	0.59010700
H	0.56231700	-0.03630500	-3.14376900
H	8.82930800	-1.18283600	8.55271200
H	7.70612600	-2.52873100	8.34769000
H	9.69511800	-1.28121400	2.85009900
H	9.04583800	-2.42343900	4.03947000
H	4.21822700	-6.65134900	5.12347700
H	5.24498100	-5.56415400	6.07072100
H	4.20238400	6.80538200	3.83640900
H	3.62618000	7.94062600	2.60274200
H	6.77524600	8.47201900	-3.37125800
H	6.82910100	8.00467900	-1.66493500
H	7.56502000	7.43747900	1.03407400
H	8.45415600	7.05305000	-0.45027500
H	-0.12429900	7.51534000	-6.57974900
H	-1.06655900	8.83499900	-5.87382900
H	-2.91428300	-1.86903400	-8.29674400
H	-3.05242000	-0.32098500	-9.16396100
H	6.01763900	-2.78848500	-5.73087200
H	5.91295200	-1.09365700	-6.24687500
H	-8.76822800	-7.04603000	0.89841700
H	-11.17167200	2.13399200	3.13288700
H	4.78432700	-9.61012300	-1.37715400
H	-4.88718300	1.76988000	-3.87696500
H	-6.55077800	1.86137000	-4.50662300
H	-4.04426300	-5.56749300	-1.08571500
H	-6.76846000	-3.00242400	-3.12648600
H	-3.93155900	5.80528700	-3.82275600
H	-4.44680400	4.24766400	-3.11669700
H	9.36424600	-1.31367600	-2.62411600
H	7.90765400	-1.70817700	-1.68936800
H	8.88526700	-3.93783100	-1.19734700
H	8.63614000	-5.67973500	-1.37058700
H	1.71164100	-3.96895700	-7.93844300
H	2.59971400	-5.45962200	-7.62426300
H	-0.85327900	-8.83095000	-1.69709400
H	0.57112100	-9.25696700	-2.65348300
H	-3.52500200	-0.62572600	-3.05950400
O	-6.02353800	-0.63267400	0.18488400
H	-6.16660400	-0.08855800	-0.61967000
H	-5.04954800	-0.72040200	0.27981700

Int7 (8.9)

C	17.59405700	43.88492800	-6.44702400
C	18.92421800	44.55088600	-6.06158200
C	20.00506100	43.55304000	-5.73442000
C	19.86548100	42.19863900	-5.54448600
C	21.41161400	43.83560600	-5.56571600
C	22.06857000	42.60078300	-5.29381100
C	22.18253600	45.00806600	-5.63624700
N	21.09685600	41.62375000	-5.27073400
C	23.45736900	42.50860100	-5.14915400
C	23.56007700	44.92597500	-5.47736200
C	24.19438100	43.68652600	-5.25350800
H	17.72140600	43.31619100	-7.37560100
H	18.76526200	45.21012700	-5.19689700

H	19.27125200	45.20461700	-6.87231000	H	29.33978300	47.98378200	-3.71811400
H	18.98329200	41.57835700	-5.58497000	H	26.47670700	45.90140100	-5.07275500
H	21.70812000	45.96616500	-5.82462000	H	26.91991100	46.06685100	-6.77893500
H	21.25319200	40.64421900	-5.09927500	H	27.92462000	45.09761800	-5.67629700
H	23.94196800	41.54822000	-4.99484600	C	24.77592400	51.75304900	-2.65202300
H	24.16364800	45.82332400	-5.55000100	C	24.53983700	50.23268800	-2.68522100
H	25.27914700	43.65458000	-5.19153300	C	23.62342600	49.83926900	-3.85576400
C	19.01585900	39.17822800	-2.73323000	C	24.01287800	49.74053100	-1.33113200
C	19.81518200	40.21018100	-1.91449600	C	23.38811200	48.33358800	-4.00744100
C	21.11446400	39.65575200	-1.36362000	H	23.83084100	52.29520200	-2.53179200
C	22.34995100	39.92933100	-1.95895800	H	25.51505000	49.74957700	-2.85554400
C	21.11972100	38.83524200	-0.22388700	H	24.06089400	50.23018700	-4.78392100
C	23.54429000	39.41053400	-1.45056700	H	22.65709400	50.34960700	-3.73647300
C	22.29679400	38.30755300	0.29682700	H	23.03371200	50.18382900	-1.11016700
C	23.52188000	38.59967300	-0.31286600	H	23.89993000	48.65290500	-1.30390000
O	24.65289400	38.07064000	0.25356100	H	24.69689500	50.01617700	-0.52226400
H	19.56749400	38.95785000	-3.65888500	H	22.83890500	48.11278800	-4.92831800
H	19.20330600	40.57991300	-1.08332900	H	24.33678500	47.78659300	-4.04870700
H	20.03416200	41.07151600	-2.54471000	H	22.81048600	47.91989300	-3.17610300
H	22.39057200	40.59030300	-2.82111000	C	27.87193300	52.02401100	-0.48636000
H	20.17870700	38.61571000	0.27554900	C	29.16154700	51.22924300	-0.70550000
H	24.49009500	39.66295600	-1.92171800	C	28.89763900	49.74046800	-0.96052200
H	22.29247100	37.70988600	1.19932500	S	28.17152100	48.89981300	0.51070700
H	25.43514000	38.34147600	-0.25337100	C	28.02659700	47.21197100	-0.16981000
C	18.62705800	45.31686000	-1.30402200	H	27.37096800	51.66829500	0.42174200
C	19.90773700	45.01976000	-2.09644400	H	29.83659700	51.34089600	0.15150800
C	21.11580800	45.75690600	-1.50903100	H	29.70034700	51.61639000	-1.57936400
C	20.15669400	43.51693600	-2.15622700	H	29.82475100	49.22126800	-1.21520000
H	18.75553100	45.03443600	-0.25187200	H	28.20059000	49.62255800	-1.79873800
H	19.76445400	45.37809900	-3.12576800	H	27.28181900	47.17159100	-0.96829300
H	22.01384800	45.58769200	-2.11303800	H	28.99161000	46.86700300	-0.55192900
H	20.94328600	46.83812700	-1.45583900	H	27.70777200	46.55869200	0.64514200
H	21.32586100	45.40214800	-0.49372400	C	21.29409600	46.95195300	2.73890600
H	20.29233900	43.10199100	-1.15068300	C	22.79715000	47.24662300	2.62387500
H	19.30303900	43.01191500	-2.61510900	C	23.48105000	46.27784900	1.65604600
H	21.03925600	43.27922300	-2.75719200	C	23.03382100	48.69827800	2.18942200
C	27.28149700	48.52820400	-5.54592900	H	20.82772600	46.98433700	1.74579200
C	28.13607300	47.25873200	-5.40036600	H	23.24513300	47.11613700	3.61976200
C	28.70317500	47.13231300	-3.98033500	H	24.56192100	46.44683900	1.61084700
C	27.32340400	46.00838800	-5.76213400	H	23.32556100	45.23600900	1.94291100
H	26.36066300	48.41836400	-4.95838700	H	23.08637400	46.40173500	0.64181200
H	28.98333900	47.33253400	-6.09739900	H	24.10347500	48.91720200	2.10480500
H	27.89080400	47.08686000	-3.24564400	H	22.58168400	48.88544800	1.20843800
H	29.29354000	46.21595900	-3.86820300	H	22.59764200	49.40802400	2.90060500

C	19.41392900	43.82101900	3.87590100	C	23.31798700	35.84495200	4.34310100
C	19.95916600	42.74407400	2.98162700	H	22.37778400	36.37164700	4.15702300
O	19.56607900	42.56498300	1.83297400	H	24.68775400	37.03716900	3.32495200
H	19.76769700	43.79354200	4.90750700	C	32.73989600	40.47104000	6.36302100
N	20.95267800	42.00346100	3.55322100	C	32.29018900	40.81175400	4.94521400
C	21.66325500	40.99192500	2.82113400	O	31.88107400	39.65089200	4.23554000
C	21.51347400	39.59060800	3.39896300	H	33.53357400	39.71778600	6.34247200
O	22.44548800	38.78663700	3.37008800	H	33.12098900	41.31619900	4.42239800
H	22.73172700	41.18751900	2.77729100	H	31.46410600	41.54005600	4.99090700
H	21.28405400	40.99409900	1.79518700	H	31.47617500	39.97213300	3.39381200
H	21.27726400	42.24434300	4.47766200	C	34.70888900	47.89997100	1.13604800
N	20.29868500	39.28130900	3.90919300	C	33.58158700	47.70783100	0.11535400
C	20.04491900	37.96009300	4.44704600	C	33.57966200	48.72906400	-1.01969600
H	20.81380900	37.70573500	5.18182300	S	32.25832800	48.43469200	-2.26420500
H	20.06815200	37.20029600	3.65752700	C	32.95772200	47.00412300	-3.15761400
H	19.57003900	39.97738900	3.88333000	H	34.62974700	47.09736500	1.88095000
C	24.72092000	44.88169400	6.12179500	H	32.61359400	47.75404800	0.62721700
C	25.09058700	44.63231700	4.64973600	H	33.65236100	46.69617100	-0.29409200
C	26.56000100	44.86455200	4.39281900	H	34.54339200	48.75380200	-1.54114600
C	27.52165700	44.01219900	4.95421600	H	33.39322300	49.73890800	-0.64007600
C	26.99713800	45.97490400	3.65992100	H	32.18197800	46.64694400	-3.83850500
C	28.88187300	44.28023700	4.80558200	H	33.83476200	47.29909100	-3.73973500
C	28.35842400	46.24430000	3.50267100	H	33.22190700	46.19470700	-2.47623400
C	29.30494100	45.40110300	4.08581300	C	34.00198300	39.69598700	1.51288500
H	25.12218900	44.06759600	6.73328100	C	33.47560800	38.38906900	0.96940700
H	24.50488300	45.28295900	3.99606500	O	33.72924500	38.03313100	-0.19569100
H	24.82534300	43.59860600	4.40456000	H	33.81294100	39.81410200	2.57984300
H	27.18816400	43.14178500	5.51225300	N	32.65407900	37.68630700	1.78496100
H	26.26225300	46.64678600	3.22484600	C	31.89705600	36.54096800	1.30799700
H	29.61491600	43.61439200	5.25373000	C	30.40471600	36.75280800	1.44475000
H	28.67794900	47.11977300	2.94413300	O	29.95132600	37.85376800	1.78539500
H	30.36317300	45.61379200	3.97418300	H	32.07366800	36.47789600	0.23187100
C	26.40696100	40.65000600	8.06106300	H	32.45227000	38.06711800	2.70599800
C	25.87906300	40.54351900	6.62991700	N	29.62184200	35.72175400	1.07015800
O	25.23970500	41.44967500	6.09836700	C	28.19072600	35.92339100	0.98309300
H	27.12431800	39.86939500	8.32280900	C	27.58878700	35.74567300	-0.41125500
N	26.21658800	39.39015800	5.97301200	C	28.28243200	36.57844300	-1.50111800
C	25.52006200	38.92406400	4.78072400	C	28.36357900	38.07952200	-1.31626400
C	24.56492200	37.77897300	5.18871000	O	27.18282300	38.69275000	-1.07630600
O	24.02320800	37.76520800	6.28629400	O	29.39120000	38.72444600	-1.44011300
H	26.23197700	38.60929200	4.01102600	H	28.02645800	36.95669700	1.29906500
H	24.91980600	39.74852200	4.39074900	H	26.52679100	36.00786800	-0.34998900
H	26.58797300	38.65397300	6.55677300	H	27.64576500	34.69723500	-0.72357200
N	24.43479500	36.77936700	4.27175300	H	27.75858500	36.41865000	-2.44993500

H	29.30962900	36.23990500	-1.63951400	C	34.52732300	39.26496900	-5.76436800
H	30.02287300	34.84946800	0.72614600	C	34.62004500	38.49151600	-3.48608800
C	29.29887800	31.23701300	-1.09695300	C	33.64898400	40.27732900	-5.38424500
C	30.13318100	32.49725000	-1.20847800	C	33.75056700	39.51307500	-3.10019600
O	30.32377200	33.22723600	-0.22700600	C	33.24616400	40.39444200	-4.05269100
H	28.24210700	31.51230700	-1.02022300	H	34.97370600	36.71838400	-7.19093100
H	29.42623800	30.56187800	-1.94597000	H	36.79217600	37.52138000	-5.73690800
N	30.65054600	32.76843900	-2.42833000	H	36.17453800	36.61180100	-4.36181600
C	31.41910900	33.96308900	-2.68092900	H	34.80661800	39.15967100	-6.80821400
C	31.10482900	34.48614500	-4.08984300	H	34.98729900	37.79618600	-2.73640200
O	30.65751300	33.72775600	-4.95339100	H	33.26101300	40.96762300	-6.12766800
C	32.94967700	33.70748700	-2.51529000	H	33.47166700	39.60117300	-2.05612700
C	33.50359100	32.71477600	-3.52405100	H	32.52820900	41.15227400	-3.77034200
O	33.69356100	34.91593300	-2.67013500	H	33.71163500	35.62714400	-4.75053000
H	31.14093200	34.71041000	-1.93356100	C	32.57873800	43.59885600	-0.69905600
H	33.07920700	33.31368000	-1.49665400	O	33.43426300	44.29294100	0.13035500
H	30.37111100	32.25635600	-3.25737100	C	31.16481100	44.17436200	-0.62152400
H	34.57148100	32.56737300	-3.34572400	C	30.23232000	43.39772500	-1.61375000
H	32.99810400	31.75048400	-3.43026300	C	30.94917200	43.29639900	-2.94579300
H	33.36668700	33.08013200	-4.54537600	C	32.28041400	43.49906700	-3.05867200
H	33.34234800	35.58431000	-2.06324800	O	33.11714700	43.73402600	-2.04352900
N	31.41133100	35.78931700	-4.27329400	C	30.64633300	44.22460600	0.79483000
C	31.38106100	36.46306900	-5.56015700	C	31.08025400	43.48923600	1.81701400
C	32.71226500	36.45434200	-6.33255500	C	29.75419400	42.01562400	-1.08090700
O	32.73969600	36.96414800	-7.45064200	C	28.52456100	42.12902200	-0.14615500
C	30.91783900	37.93552500	-5.40612500	C	30.17527100	43.04539900	-4.16648800
C	29.49211600	38.22559500	-5.90258400	O	28.95524500	43.09691300	-4.24518200
C	29.34825000	37.97447500	-7.40814800	O	30.94749400	42.73016100	-5.23690600
C	28.43592300	37.46582900	-5.09541100	C	30.23777000	42.46860100	-6.45690900
H	30.68818000	35.90390000	-6.19411700	O	31.10207200	40.35793000	1.72998100
H	31.60666300	38.57279100	-5.96022900	C	34.71269800	43.68347800	0.29195700
H	31.00973500	38.22354900	-4.35356200	H	31.23788300	45.20531600	-0.99399600
H	29.32705100	39.29944800	-5.72775800	H	29.34047100	44.01394400	-1.77901500
H	28.37191700	38.31625200	-7.76839500	H	32.59160900	42.52020000	-0.47910400
H	30.13205200	38.48940500	-7.97043600	H	32.78995300	43.50409400	-4.01406400
H	29.43230800	36.90938100	-7.64711400	H	29.83333700	44.92856200	0.96414400
H	31.70042400	36.33791500	-3.46211800	H	30.61691500	43.57572600	2.79127800
H	27.42361600	37.69408800	-5.44576700	H	31.87864200	42.76326800	1.72728000
H	28.57895600	36.38179800	-5.17025600	H	30.56075600	41.52912100	-0.52705700
H	28.50251200	37.74487900	-4.04076900	H	29.53332600	41.35562900	-1.92330200
N	33.79954000	35.90976700	-5.72309300	H	28.53401800	43.14890800	0.24982600
C	35.12944800	36.23746200	-6.22418200	H	29.51465600	41.66288900	-6.31323000
C	35.87083800	37.17715500	-5.24992900	H	29.71142700	43.36318500	-6.79881900
C	35.01083900	38.34644600	-4.82161700	H	30.99793000	42.17202700	-7.17841800

H	35.25976000	43.64534300	-0.65525300
C	27.93199100	41.80462900	2.24603700
N	28.64972700	41.29312100	1.06595500
C	26.41855900	41.56926400	2.29450400
N	25.57411500	42.38103900	-2.36076100
C	25.55619400	42.18815700	1.21892000
C	24.26631200	42.61906800	1.51854900
C	23.32064000	42.94015200	0.52477700
C	23.64041200	42.85213300	-0.82136500
C	24.95381200	42.49407200	-1.13028400
C	26.91341900	42.11279900	-2.18389900
C	27.18662000	41.98510000	-0.83230600
C	25.92723400	42.19759000	-0.14173700
H	28.37300800	41.32564200	3.12752900
H	28.35255200	40.33807500	0.87059000
H	30.26819400	40.87179200	1.54086200
H	26.05791400	41.87738100	3.28169300
H	26.24965900	40.48142600	2.24558400
H	25.13712900	42.62855600	-3.23630400
H	23.97895900	42.69126300	2.56137000
H	22.31623700	43.23174400	0.81447300
H	22.91116800	43.06077900	-1.59351000
H	27.59010200	42.13878300	-3.02387500
H	27.30145500	39.67466900	-1.11786900
H	28.15428200	42.87099000	2.32794100
H	16.79250700	44.60997500	-6.62320200
H	17.36376100	43.15243100	-5.66638700
H	18.42039300	46.39115100	-1.35438400
H	17.78087500	44.74148600	-1.69371500
H	19.01188400	38.23043600	-2.18424600
H	18.03106300	39.53859800	-3.04746800
H	26.97394500	48.70055500	-6.58380600
H	27.79503100	49.40665400	-5.14355900
H	28.01755200	53.10049300	-0.34816100
H	27.17598400	51.81304300	-1.30458900
H	25.25397200	52.09521800	-3.57582600
H	25.41493600	51.98473100	-1.79345900
H	34.62259200	48.82957200	1.70823800
H	35.70658500	47.78937600	0.69826800
H	31.92266400	40.09420200	6.98670700
H	33.13799500	41.38125000	6.82351100
H	23.63384000	44.86043600	6.25100400
H	25.16675900	45.81304100	6.48736400
H	29.57174200	30.72193600	-0.16999100
H	35.72522400	35.33518300	-6.39719500
H	19.06474400	37.92146200	4.93345100
H	33.39884400	40.43940500	0.98142100
H	35.06275300	39.83935200	1.28218200
H	27.65418000	35.32003200	1.72278400
H	32.33088300	35.61953900	1.71009700
H	35.25883100	44.29958800	1.00715800
H	34.60997500	42.66566600	0.69083300
H	20.80035500	47.71471300	3.35023600
H	21.08469200	45.96235600	3.15831800
H	19.67894000	44.77709700	3.41262600
H	18.31969800	43.84017600	3.84191700
H	26.86447400	41.63371000	8.20951000
H	25.59498500	40.65069700	8.79561600
H	23.46499100	35.10794900	3.54664900
H	23.21918400	35.29450200	5.28438600
H	30.81438500	39.43552800	1.57724600
O	31.74710200	37.18575100	-1.83284800
H	32.49369600	37.61122700	-1.35561800
H	30.98562800	37.79044600	-1.73420200

E:P (-7.8)

C	17.59407800	43.88493800	-6.44700600
C	18.92004800	44.56614200	-6.07558800
C	20.01960100	43.58762900	-5.75661700
C	19.89755000	42.24326000	-5.49551300
C	21.43021100	43.88449100	-5.66757600
C	22.10620800	42.66723700	-5.36277600
C	22.19030000	45.05450800	-5.83375300
N	21.14310000	41.68776100	-5.25140900
C	23.49919600	42.58958700	-5.26539100
C	23.57461800	44.98570600	-5.73130700
C	24.22578300	43.76273800	-5.46437900
H	17.72034600	43.31471900	-7.37478400
H	18.76022600	45.22850400	-5.21323800
H	19.24842300	45.22058800	-6.89336600
H	19.01817100	41.61830100	-5.46422500
H	21.70199300	45.99957200	-6.05110100
H	21.31298600	40.72283000	-5.01920600
H	23.99955100	41.64791900	-5.06036600
H	24.16939600	45.88107600	-5.87456200
H	25.31126800	43.72812800	-5.43329100
C	19.01585700	39.17823600	-2.73328700
C	19.83305800	40.22238000	-1.93898700

C	21.17502600	39.72765800	-1.41714400	H	23.82825200	52.29017200	-2.53130600
C	22.39401900	40.13470300	-1.97585700	H	25.52777500	49.75585700	-2.86968300
C	21.24554600	38.86702000	-0.30880900	H	24.04333500	50.22913900	-4.77750500
C	23.62779100	39.69968500	-1.47538500	H	22.65322400	50.33166000	-3.71052600
C	22.46080200	38.42294900	0.20372800	H	23.06962600	50.16732500	-1.08619900
C	23.66992800	38.83228300	-0.37797100	H	23.93851000	48.64141900	-1.30068700
O	24.82153800	38.36236400	0.18774200	H	24.74297200	50.00462400	-0.52578300
H	19.55671300	38.94796700	-3.66348600	H	22.82756600	48.10128600	-4.90685600
H	19.23864900	40.58421500	-1.09106600	H	24.34786300	47.78237000	-4.06656000
H	20.00727400	41.08675600	-2.57952400	H	22.84193700	47.90258700	-3.15642200
H	22.39103700	40.84172600	-2.80193700	C	27.87195500	52.02406400	-0.48619300
H	20.32452500	38.54549000	0.17309200	C	29.15255300	51.21374100	-0.70046400
H	24.55385700	40.05775000	-1.91637800	C	28.86311600	49.73292400	-0.97944100
H	22.49553200	37.78755600	1.08017900	S	28.09675200	48.88587300	0.46868500
H	25.62718700	38.55516200	-0.33632900	C	27.73573900	47.27157600	-0.30258900
C	18.62702600	45.31686700	-1.30402700	H	27.36532600	51.67181500	0.41994600
C	19.89558600	45.04511500	-2.12270900	H	29.82004100	51.30312200	0.16518500
C	21.08361400	45.84422200	-1.57660300	H	29.70891700	51.60100700	-1.56305600
C	20.18150700	43.54875200	-2.17414800	H	29.78193200	49.19910600	-1.23522200
H	18.77215400	45.02976500	-0.25588300	H	28.16957100	49.64362600	-1.82422600
H	19.71494300	45.38518800	-3.15222400	H	26.96294800	47.36664600	-1.06974500
H	21.98014900	45.69308300	-2.18771700	H	28.63984000	46.84541700	-0.74657500
H	20.86749700	46.91865800	-1.56109900	H	27.37512400	46.60475900	0.48348600
H	21.32033800	45.53970200	-0.55102100	C	21.29414200	46.95201900	2.73894300
H	20.32427400	43.13753400	-1.16836600	C	22.78913700	47.29507000	2.63022100
H	19.33608200	43.02721500	-2.62862800	C	23.51987800	46.38757300	1.63930700
H	21.06668500	43.32165200	-2.77592700	C	22.96766000	48.76703200	2.23462400
C	27.28116300	48.52808700	-5.54640500	H	20.82816400	46.98460600	1.74568800
C	28.13687700	47.25694600	-5.43850100	H	23.24012000	47.15924100	3.62411600
C	28.70922400	47.07335800	-4.02723200	H	24.58566300	46.63375000	1.58343200
C	27.31549300	46.02876300	-5.84913700	H	23.44147200	45.33383100	1.90899300
H	26.36342000	48.40844000	-4.95667200	H	23.10356100	46.49994300	0.63205400
H	28.98279700	47.35524300	-6.13399900	H	24.02792700	49.03060400	2.16058600
H	27.90174500	46.95784800	-3.29496400	H	22.51243100	48.96059400	1.25617100
H	29.33643500	46.17557900	-3.97232900	H	22.50025600	49.44072100	2.96096300
H	29.31807300	47.92964100	-3.71837700	C	19.41391300	43.82105400	3.87592600
H	26.47502500	45.89126900	-5.15790200	C	19.95174000	42.73693900	2.98626700
H	26.90173000	46.13970300	-6.85743800	O	19.54281100	42.54406000	1.84575900
H	27.90639500	45.10976700	-5.82062500	H	19.76752600	43.79519400	4.90775400
C	24.77589600	51.75307500	-2.65197100	N	20.95785900	42.00827200	3.55133200
C	24.55047400	50.22998100	-2.68530700	C	21.66324300	40.99181800	2.82117600
C	23.62246500	49.83063800	-3.84487300	C	21.51105700	39.59040000	3.39841700
C	24.04708900	49.72969700	-1.32492000	O	22.44197700	38.78508200	3.36666500
C	23.39718200	48.32352100	-3.99909200	H	22.73081700	41.17924700	2.77903500

H	21.28672000	40.99353600	1.79433800	H	31.05649300	39.24674800	4.43744200
H	21.28760900	42.25509900	4.47222700	C	34.70903600	47.90002200	1.13593900
N	20.29956700	39.28429400	3.91795000	C	33.58595100	47.71479000	0.10716500
C	20.04491200	37.96008600	4.44701500	C	33.60561500	48.74779700	-1.01862600
H	20.81330700	37.70133500	5.18061300	S	32.29262400	48.50418500	-2.28091000
H	20.06747300	37.20453800	3.65335000	C	32.97537900	47.08023600	-3.19865800
H	19.56455500	39.97278400	3.87453200	H	34.63000200	47.09762700	1.88091100
C	24.72084200	44.88173000	6.12180000	H	32.61352000	47.76689700	0.60979100
C	25.01269900	44.64746300	4.62689200	H	33.64953900	46.70672500	-0.31756100
C	26.47037200	44.73877700	4.24350100	H	34.57438000	48.76149300	-1.53119400
C	27.36228900	43.70622600	4.56809300	H	33.43747500	49.75491300	-0.62346600
C	26.96734600	45.86207800	3.56989200	H	32.22866100	46.79894800	-3.94442000
C	28.71405700	43.80434200	4.23535200	H	33.89923500	47.35813400	-3.71308000
C	28.31818100	45.96388700	3.23195200	H	33.15479500	46.22539300	-2.54651600
C	29.19795400	44.93391800	3.56909600	C	34.00192700	39.69598000	1.51282600
H	25.13239600	44.05532100	6.70881200	C	33.49789800	38.37703500	0.96996500
H	24.44610000	45.36448200	4.02777100	O	33.79794300	38.00469200	-0.17501300
H	24.63775100	43.65178800	4.37025100	H	33.83002800	39.80547800	2.58357800
H	26.98069900	42.83219900	5.08820900	N	32.65963800	37.68664300	1.77665600
H	26.28748700	46.66963300	3.31224400	C	31.89704700	36.54105000	1.30780800
H	29.39441900	42.99862000	4.50141400	C	30.41136900	36.73071500	1.55274600
H	28.68333500	46.84955600	2.71922700	O	29.98405600	37.76775100	2.09109000
H	30.25263900	45.02051300	3.32642200	H	32.03076800	36.47586300	0.22608200
C	26.40697100	40.65000300	8.06105500	H	32.39712400	38.11372400	2.65986700
C	25.87785200	40.53825800	6.63119600	N	29.62903200	35.75124900	1.06743000
O	25.23662800	41.44072700	6.09698500	C	28.19112600	35.92311000	0.98314300
H	27.12329900	39.86881700	8.32463900	C	27.59213800	35.69874100	-0.40479400
N	26.22425000	39.38633000	5.97274800	C	28.24467600	36.54047400	-1.51029100
C	25.52006000	38.92406600	4.78072800	C	28.44828400	38.01253500	-1.14896400
C	24.57162500	37.77457700	5.19215200	O	27.47522400	38.67397400	-0.67924300
O	24.04358100	37.75736100	6.29706900	O	29.60504900	38.50490300	-1.35033900
H	26.22634000	38.61552700	4.00332400	H	28.00172000	36.96022500	1.26991300
H	24.91311900	39.74786300	4.39899800	H	26.53020800	35.95541800	-0.32634500
H	26.55732600	38.64143100	6.56943900	H	27.65462500	34.64138100	-0.68619600
N	24.43377100	36.78094400	4.27305700	H	27.61293200	36.50068200	-2.40359600
C	23.31798600	35.84495800	4.34310300	H	29.21422600	36.12483600	-1.79007200
H	22.37860400	36.37236300	4.15561100	H	30.04351700	34.92677400	0.63101400
H	24.69525300	37.03501300	3.32773300	C	29.29899800	31.23697500	-1.09701400
C	32.74013600	40.47105300	6.36302800	C	30.13063900	32.49992800	-1.20840400
C	32.29294800	40.71749200	4.92228100	O	30.31048500	33.23350500	-0.22745000
O	31.96035600	39.51013800	4.22323300	H	28.24199200	31.51142300	-1.02155200
H	33.53645700	39.72027300	6.37178300	H	29.42860300	30.56200200	-1.94593200
H	33.11172500	41.16704700	4.35175000	N	30.65660100	32.76475900	-2.42556900
H	31.45184600	41.42366200	4.88586800	C	31.41898000	33.96304000	-2.68096200

C	31.11207400	34.47091600	-4.09856700	H	35.04734100	37.75385000	-2.70442700
O	30.68763900	33.70134700	-4.96346500	H	33.30821600	41.00537500	-6.01304500
C	32.94953900	33.71983600	-2.50111300	H	33.60226100	39.58782300	-1.95971800
C	33.51756600	32.71392600	-3.48905700	H	32.68200600	41.20611900	-3.62171200
O	33.68392900	34.93151900	-2.66445000	H	33.71429900	35.63005800	-4.74460300
H	31.12822900	34.71408300	-1.94357000	C	32.25426100	43.50966400	-1.91216500
H	33.07215500	33.34108000	-1.47534000	O	33.22981800	44.14136100	-1.16900600
H	30.38227000	32.24865800	-3.25386300	C	30.87213900	44.09856000	-1.62912300
H	34.58722500	32.58444400	-3.30601600	C	29.79969800	43.35147900	-2.50446200
H	33.02502600	31.74476900	-3.37683000	C	30.36110200	43.16856300	-3.90394100
H	33.37770200	33.05723300	-4.51760800	C	31.65892700	43.39855800	-4.19664700
H	33.25924500	35.62197200	-2.12981200	O	32.60799400	43.70491400	-3.30387800
N	31.39958900	35.77802200	-4.28098000	C	30.56256600	44.13903800	-0.15069600
C	31.38100200	36.46293200	-5.56001500	C	31.07596200	43.34311800	0.78801000
C	32.70493800	36.41072000	-6.34828200	C	29.31155600	42.00302100	-1.91226000
O	32.72914200	36.88299600	-7.48320200	C	28.27624200	42.17720000	-0.78408700
C	31.02686700	37.96396000	-5.37043100	C	29.46323500	42.77172900	-4.99863900
C	29.59416400	38.37023400	-5.74893500	O	28.24180700	42.76362800	-4.93423600
C	29.30947400	38.14405200	-7.23808000	O	30.12154900	42.39134600	-6.11749700
C	28.55051900	37.69871100	-4.85517500	C	29.28806800	42.02325200	-7.23023800
H	30.64738000	35.96135900	-6.19562100	O	30.91099600	39.94862000	0.49008700
H	31.71213100	38.55382900	-5.97850800	C	34.49435900	43.47731200	-1.20550400
H	31.22213600	38.23912800	-4.32871600	H	30.90589300	45.13425500	-1.99028400
H	29.54118700	39.45309600	-5.56091200	H	28.93106500	44.01471000	-2.58652200
H	28.33835300	38.56705300	-7.51919900	H	32.26952000	42.42073600	-1.74385900
H	30.08503700	38.59632000	-7.86349000	H	32.04487000	43.35142000	-5.20742200
H	29.28261800	37.07714000	-7.48284000	H	29.83955900	44.89700000	0.14762200
H	31.69549400	36.32166800	-3.46889900	H	30.76596500	43.44326200	1.82202400
H	27.53627500	38.02792100	-5.10681100	H	31.79032400	42.55690600	0.57380500
H	28.58298200	36.60796900	-4.95729300	H	30.17596600	41.45152500	-1.53337700
H	28.74414400	37.94824300	-3.81037200	H	28.88323300	41.38509400	-2.70438300
N	33.80131500	35.90762300	-5.71932300	H	28.54918600	43.06714300	-0.21581300
C	35.13002400	36.23803300	-6.22479200	H	28.62067300	41.20656600	-6.95084100
C	35.87739500	37.16200300	-5.24038700	H	28.69293300	42.87684200	-7.56361600
C	35.03584000	38.33366100	-4.78198400	H	29.97473100	41.70197800	-8.01187900
C	34.54454700	39.27193500	-5.70108000	H	34.90576100	43.46022300	-2.21889700
C	34.68442000	38.47143600	-3.43467100	C	27.96671600	41.40975900	1.61358700
C	33.70836800	40.30578700	-5.28452000	N	28.41977200	41.06950700	0.22772400
C	33.85723600	39.51315100	-3.01118800	C	26.47053800	41.28256200	1.85097700
C	33.35796000	40.42348000	-3.93836200	N	25.08000500	43.08925400	-2.37214700
H	34.97297900	36.72341900	-7.18908100	C	25.53925300	42.16479800	1.05530100
H	36.79922700	37.50832000	-5.72536300	C	24.32298200	42.53554400	1.61779900
H	36.18217000	36.58021500	-4.36343900	C	23.26481200	43.05816400	0.85171400
H	34.78647600	39.16847800	-6.75454700	C	23.39994200	43.24580300	-0.51431700

C	24.64592200	42.96483500	-1.07133500
C	26.40507800	42.72514200	-2.45716300
C	26.85366500	42.34043300	-1.21219900
C	25.73545700	42.46202400	-0.30810600
H	28.49245700	40.71132100	2.27092200
H	27.96591000	40.17791600	-0.13071400
H	30.54605600	39.40317300	-0.24975800
H	26.30475600	41.45366500	2.91983800
H	26.17628300	40.24140100	1.65365300
H	24.51920800	43.39387700	-3.15630000
H	24.18659200	42.37501000	2.68111300
H	22.31594200	43.28692700	1.32343300
H	22.57941400	43.59743300	-1.12085800
H	26.94325800	42.80293500	-3.38955400
H	29.43039500	40.81890700	0.32070200
H	28.32387000	42.41782600	1.83574300
H	16.79251700	44.60998900	-6.62318900
H	17.36378600	43.15239300	-5.66641600
H	18.42040300	46.39115300	-1.35436700
H	17.78087100	44.74148600	-1.69370900
H	19.01187900	38.23044500	-2.18423000
H	18.03105300	39.53858400	-3.04745200
H	26.97414800	48.70077400	-6.58383000
H	27.79507200	49.40647300	-5.14321600
H	28.01753700	53.10051100	-0.34828400
H	27.17598600	51.81294900	-1.30456800
H	25.25399400	52.09519400	-3.57582400
H	25.41495200	51.98480600	-1.79349200
H	34.62248600	48.82952200	1.70830200
H	35.70661300	47.78942500	0.69832000
H	31.92270700	40.09410000	6.98670200
H	33.13777500	41.38132700	6.82354900
H	23.63384000	44.86048100	6.25101300
H	25.16680600	45.81300800	6.48738900
H	29.57166000	30.72196400	-0.16995100
H	35.72477100	35.33479400	-6.39672800
H	19.06475100	37.92148400	4.93346900
H	33.39886500	40.43938300	0.98136700
H	35.06277700	39.83932200	1.28227500
H	27.65394000	35.32022100	1.72276300
H	32.33076900	35.61955700	1.71026200
H	35.15583500	44.03881700	-0.54520800
H	34.40017500	42.44585000	-0.84068000
H	20.80030100	47.71468900	3.35022200
H	21.08468200	45.96237400	3.15835000
H	19.67894800	44.77706800	3.41256900
H	18.31969800	43.84018700	3.84192300
H	26.86446800	41.63371200	8.20951500
H	25.59498300	40.65070000	8.79561400
H	23.46499000	35.10795300	3.54664500
H	23.21918300	35.29450400	5.28438800
H	30.78150200	39.35706100	1.25791700
O	31.84019200	37.08230300	-1.84285800
H	32.54198700	37.58450200	-1.38089400
H	30.99075500	37.57767700	-1.71056400
